# The concluding chapter: recircumscription of *Goodenia* (Goodeniaceae) to include four allied genera with an updated infrageneric classification

**DOI:** 10.3897/phytokeys.152.49604

**Published:** 2020-07-07

**Authors:** Kelly A. Shepherd, Brendan J. Lepschi, Eden A. Johnson, Andrew G. Gardner, Emily B. Sessa, Rachel S. Jabaily

**Affiliations:** 1 Western Australian Herbarium, Department of Biodiversity, Conservation & Attractions, Kensington, WA 6151, Australia Western Australian Herbarium Kensington Australia; 2 Australian National Herbarium, Centre for Australian National Biodiversity Research, GPO Box 1700, Canberra, ACT, 2601, Australia Australian National Herbarium Canberra Australia; 3 Department of Biology, University of Mississippi, Oxford, MS 38677, USA University of Mississippi Oxford United States of America; 4 Department of Biological Sciences, California State University, Stanislaus, Turlock, CA 95382, USA California State University Turlock United States of America; 5 Department of Biology, University of Florida, Gainesville, FL 32607, USA University of Florida Gainesville United States of America; 6 Department of Organismal Biology & Ecology, Colorado College, Colorado Springs, CO 80903, USA Colorado College Colorado Springs United States of America

**Keywords:** *
Goodenia
*, Goodeniaceae, nomenclature, phylogeny, taxonomy, *
Velleia
*

## Abstract

Close scrutiny of *Goodenia* (Goodeniaceae) and allied genera in the ‘Core Goodeniaceae’ over recent years has clarified our understanding of this captivating group. While expanded sampling, sequencing of multiple regions, and a genome skimming reinforced backbone clearly supported *Goodenias.l.* as monophyletic and distinct from *Scaevola* and *Coopernookia*, there appears to be no synapomorphic characters that uniquely characterise this morphologically diverse clade. Within *Goodenias.l.*, there is strong support from nuclear, chloroplast and mitochondrial data for three major clades (Goodenia Clades A, B and C) and various subclades, which lead to earlier suggestions for the possible recognition of these as distinct genera. Through ongoing work, it has become evident that this is impractical, as conflict remains within the most recently diverged Clade C, likely due to recent radiation and incomplete lineage sorting. In light of this, it is proposed that a combination of morphological characters is used to circumscribe an expanded *Goodenia* that now includes *Velleia*, *Verreauxia*, *Selliera* and *Pentaptilon*, and an updated infrageneric classification is proposed to accommodate monophyletic subclades. A total of twenty-five new combinations, three reinstatements, and seven new names are published herein including Goodenia subg. Monochila sect. Monochila subsect. Infracta K.A.Sheph. **subsect. nov.** Also, a type is designated for Goodenia subg. Porphyranthus sect. Ebracteolatae (K.Krause) K.A.Sheph. **comb. et stat. nov.**, and lectotypes or secondstep lectotypes are designated for a further three names.

## Introduction

Representatives of the predominantly Australian family Goodeniaceae R.Br, a close relative to the cosmopolitan Asteraceae Bercht. & J.Presl (Tank and Donoghue 2010), have been the focus of various studies in recent years. The first molecular phylogeny of generic exemplars by Gustafsson et al. (1996) indicated that the monotypic and closely allied Brunoniaceae Dumort. was, in fact, embedded within Goodeniaceae. This was previously hypothesised by Carolin (1992a), due to the shared presence of a unique cup-like pollen presenter positioned at the apex of the style referred to as an indusium (Carolin 1960). Howarth et al. (2003) (and later expanded in Jabaily et al. 2014), studied the origin of Hawaiian species of *Scaevola* L., the only genus in the family with significant diversity outside of Australia (see table 1 in Jabaily et al. 2012). These studies confirmed that *Scaevola* dispersed from Australia into the islands of the Pacific at least four times, starting in the late Miocene and continuing into the Pliocene, and that homoploid hybridisation subsequently contributed to the extant diversity apparent across the islands today (Howarth and Baum 2005).

**Figure 1.**
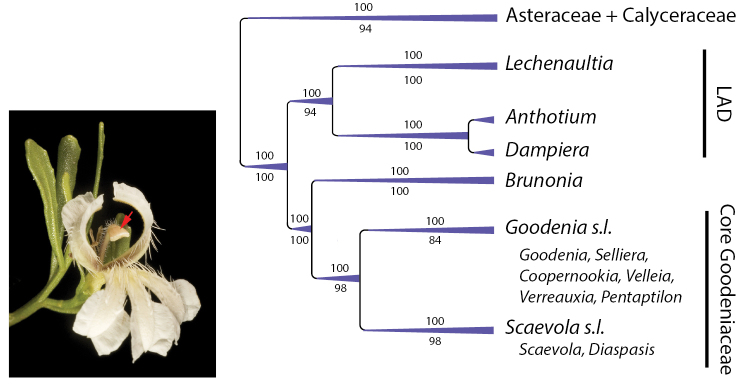
Summary of broad relationships in Goodeniaceae from Jabaily et al. (2012) based on a 50% majority-rule cladogram from a partitioned Bayesian inference analysis of *trnL-F* and *matK*, with additional bootstrap values from separate parsimony and maximum likelihood analyses (values above branches are Bayesian posterior probabilities, values below branches are maximum likelihood bootstrap). Left inset – *Coopernookia strophiolata* showing the unique indusium pollen presenter (red arrow) that is diagnostic for the family. Voucher: *K.R. Thiele* 3710. Image: K.R. Thiele.

**Figure 2.**
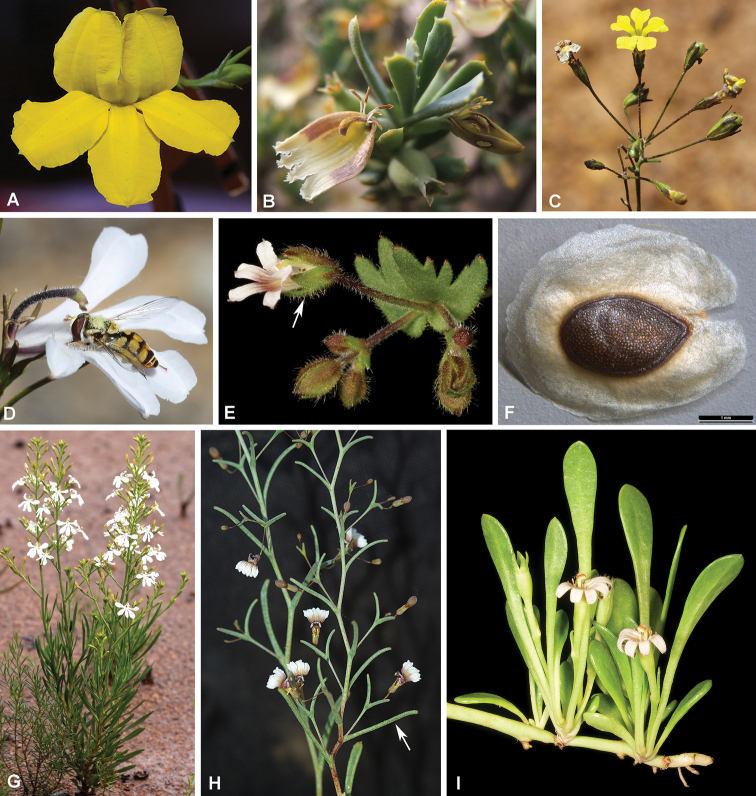
Diagnostic features of various species in *Goodenias.l.***A** the type species *Goodenia ovata***B***Scaevola collaris* fan-like flowers and immature fleshy fruit **C***G. concinna* subumbellate inflorescence **D***G. scapigera* with short, stiff hairs on the style, narrow indusium and white fan-like flowers **E***Velleia cycnopotamica* with free sepals attached below the ovary (white arrow) **F***G. vilmoriniae* seed with a broad membranous wing **G***G. scapigera* habit with cauline leaves and thyrse-like inflorescences **H***G. gypsicola* leafy bracts (white arrow) **I***Selliera radicans* with prostrate stems rooting at the nodes, and solitary, bracteolate, fan-like flowers in the leaf axils. Vouchers: *K.A. Shepherd* KS 1530 (**A**); *K.A. Shepherd* KS 1533 (**B**); *K.A. Shepherd* KS 1591 (**C**); *K.A. Shepherd* KS 1584 (**D**); *K.R. Thiele* KRT 4201 (**E**); DEM6887 (**F**); *K.A. Shepherd* KS 1468 (**G**); *R. Davis s.n.* (**H**); *J.A. Cochrane & S. Barrett* 4181 (**I**). Images: K.A. Shepherd (**A–D, G**); K.R. Thiele (**E, I**); Seeds of South Australian (**F**); R. Davis (**H**).

Our team completed the first comprehensive phylogeny of the family utilising cpDNA from 212 (out of 400+) species across 12 genera (Jabaily et al. 2012). Two major clades were identified within the family, the smaller of the two being the LAD clade composed of *Lechenaultia* R.Br., *Anthotium* R.Br., and *Dampiera* R.Br. with the remaining nine genera falling in the larger ‘Core Goodeniaceae’ clade (Fig. 1). Within the Core Goodeniaceae the monotypic *Brunonia australis* Sm. ex R.Br. placed sister to two large clades comprising *Scaevolas.l.* and *Goodenia* Sm. *s.l.*, respectively. Generic-level taxonomic problems were noted in both clades. Firstly, the monotypic *Diaspasis filifolia* R.Br. was shown to be embedded within *Scaevola* while *Goodenias.l.* (represented by a subset of 60 species), resolved into three major clades (denoted A, B, C), and was rendered paraphyletic due to the inclusion of *Coopernookia* Carolin, *Selliera* Cav. (Fig. 2I), *Velleia* Sm. (Fig. 2E), *Verreauxia* Benth., *Scaevola collaris* F.Muell. (Fig. 2B) and the monotypic *Pentaptilon* E.Pritz. This phylogeny was built using 3117 base-pairs of cpDNA including *trnL-F* and *matK*, and while phylogenetic support values were high for many smaller clades, the backbone topology of *Goodenias.l.* was weakly supported in most analyses. A few subgeneric taxonomic groupings were largely monophyletic (e.g. subg. Monochila (G.Don) Carolin, subg. Goodenia subsect. Ebracteolatae K.Krause), but many were not (e.g. subg. Goodenia subsect. Goodenia and subsect. Coeruleae (Benth.) Carolin). Furthermore, other subgeneric groupings were not included or placed (e.g. sect. Porphyranthus G.Don, sect. Amphichila DC., and ser. Calogyne (R.Br.) Carolin of subg. Goodenia).

Clarifying the relationships among *Goodenia* clades A, B, C and the smaller affiliate genera was necessary in order to identify monophyletic groups for taxonomic recognition. We could not seek to make changes to *Goodenias.l.* without, at minimum, full and consistent resolution of the backbone relationships and confidence in the species-level composition of each major clade. To try and address this issue, the power of next-generation sequencing was leveraged for a subset of taxa (Gardner et al. 2016a). Twenty-four taxa representative of almost all major clades within Core Goodeniaceae, including 19 accessions from *Goodenias.l.* (except subsect. Scaevolina and a subset of species from subsect. Goodenia placed in Clade C), were sequenced using genome-skimming technology. The majority of coding regions of the chloroplast genome were assembled and analysed, resulting in a nearly fully resolved phylogeny for all but two nodes within *Goodenias.l.* This topology was then applied as a constraint and also concatenated on an expanded matrix of 98 Core Goodeniaceae species with *trnL-F* and *matK* loci of sequence data, greatly improving phylogenetic support values. This backbone topology has been similarly utilised in the present study. The analyses of Gardner et al. (2016a) confirmed the position of *Coopernookia* as sister to the remainder of *Goodenias.l.*, followed by stepwise sisters Clade A, Clade B, and finally *Velleia* sister to Clade C. However, the composition and relationships of subclades within the most morphologically diverse Clade C were poorly resolved, except for the monophyly of subg. Monochila and subg. Goodenia subsect. Coeruleae. Exploration of the backbone phylogeny derived from additional genomic compartments (nuclear ribosomal complex, several single copy nuclear genes) in the study suggested alternative topologies compared to the plastid, though with low support.

To continue our investigation of alternative backbone topologies and delve into the poorly resolved Clade C, we expanded and further explored the next-generation sequencing data across nuclear, chloroplast and mitochondrial genomic compartments (Jabaily et al. 2018). We generated new genome skimming data for four additional taxa from within Clade C, and re-analysed the previously generated raw genomic data for the taxa in clade *Goodenias.l.*, for a total of 24 taxa. Partial mitochondrial genomes and partial chloroplast genomes expanded beyond the efforts of Gardner et al. (2016a) similarly strongly supported our original backbone relationship within *Goodenias.l.* Extensive hypothesis tests were performed to explore congruencies and determine statistical support for all possible relationships within the challenging Clade C. Still, relationships between taxa and subclades within Clade C remained poorly resolved with both mitochondrial and plastid loci, as well as an expanded nuclear data set. In conclusion, while there was strong support for the monophyly of subg. Monochila and all other subclades represented distinct lineages, their position relative to each other remained unresolved, thus precluding their recognition as well-supported genera.

## Morphology of Core Goodeniaceae

**Figure 3.**
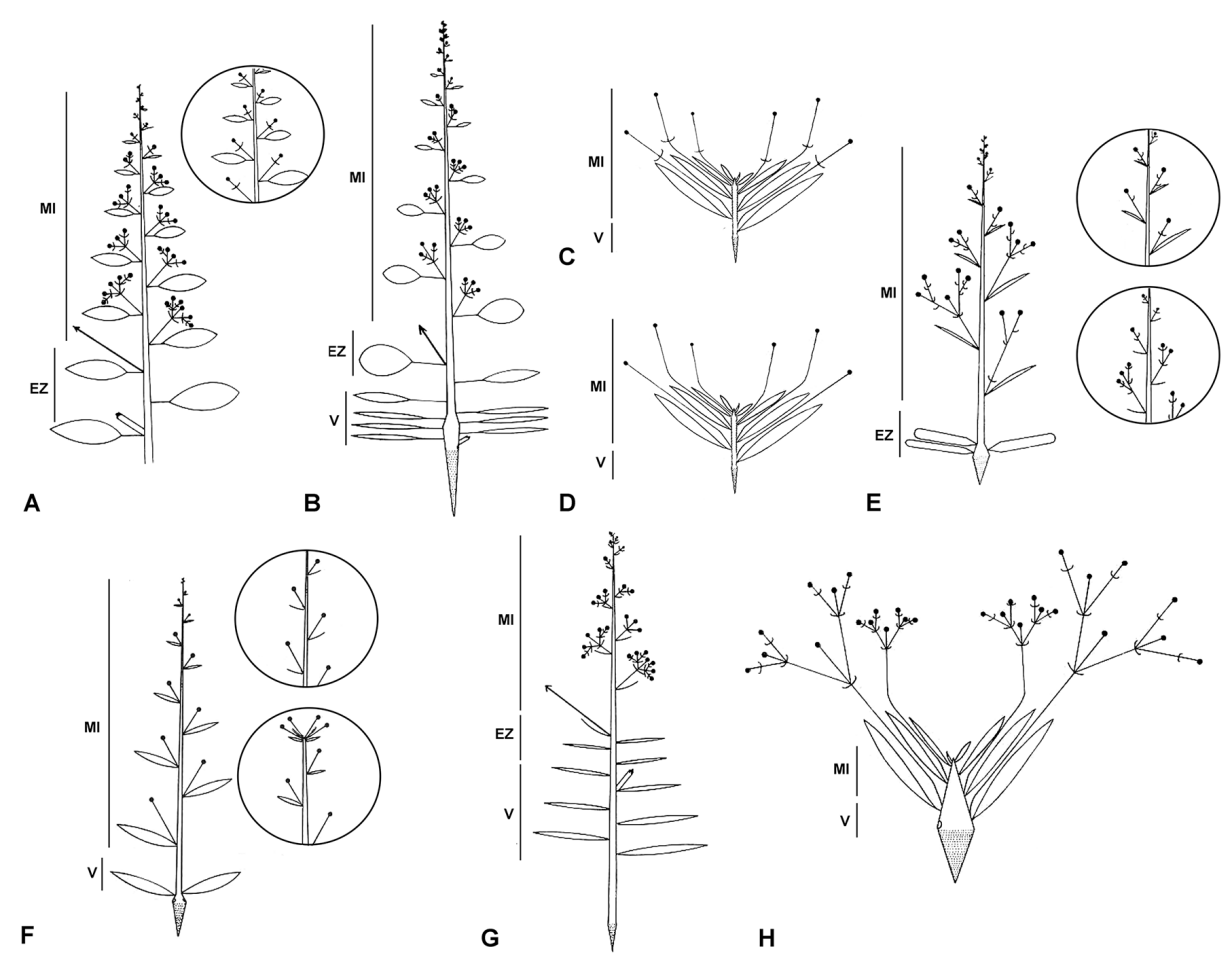
Characterisation of inflorescence structure in *Goodenias.l.* modified from Carolin (1967a) with his corresponding Bauplan ‘Type’ concepts stated were applicable and phylogenetic position for exemplar species given in brackets; MI = main inflorescence, EZ = enrichment zone, V = vegetative zone. **A** Form A (Type 1) is a thyrse with leafy bracts and bracteoles e.g. *Goodenia ovata* (Goodenia I) or (Type 2) 1(–2)-flowered raceme with leafy bracts and bracteoles e.g. *G. laevis* (Goodenia I) (inset) **B** Form B (Type 5) is a basal rosette with leafy bracts and bracteoles e.g. *G. hederacea* (Goodenia II) **C** Form C (no Type) with flowers solitary in leaf axils, leafy bracts and bracteoles e.g. *G. convexa* (Goodenia II) **D** Form D (no Type) flowers solitary in leaf axils with leafy bracts, bracteoles absent e.g. *G. pumilo* (Porphyranthus I) **E** Form E (Type 4) a basal rosette, bracteoles, with leafy bracteose bracts and either a panicle-like form e.g. *G. paniculata*, raceme e.g. *G. gracilis* (Porphyranthus II) (inset above) or a thyrse e.g. *G. pterigosperma* (Coeruleae) (inset below) **F** Form F (Type 6) with ebracteolate racemes and leafy bracts e.g. *G. hispida* (Ebracteolatae II), (Type 7) non-leafy bracts e.g. *G. cusackiana* (Ebracteolatae I) (inset above), or (Type 8) a subumbel e.g. *G . pulchella* (Ebracteolatae I) (inset below) **G** Form G (Type 3) represented by a thyrse with reduced bracts and bracteoles e.g. *G. scapigera* (Monochila) and **H** Form H (Velleia Type) is a compound dichasium with leafy bracts and bracteoles e.g *Velleia lyrata* (Velleia).

**Figure 4.**
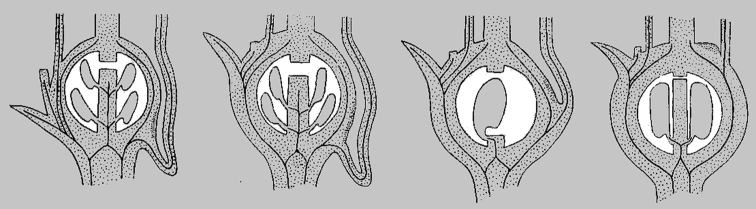
Diagramatic sections of ovaries (l.s.) modified from Carolin (1959). From left–right *Velleia*, *Goodenia*, *Verreauxia*, and *Scaevola*, showing fusion of floral parts and structure of the incomplete locules and placentation of ovules.

The *Flora of Australia* treatment of Goodeniaceae and Brunoniaceae (Carolin 1992a; Carolin et al. 1992) represented more than 30 years of research by Roger Carolin and his students. Over this period, Carolin’s team successively revised each genus through a series of detailed anatomical and morphological studies that culminated in the recognition of numerous new species and updated infrageneric classifications. Through his early cladistics work and study of inflorescence types, Carolin determined that there were two distinct assemblages within the Goodeniaceae (Carolin 1967a; Carolin 1977). The first was the *Lechenaultia*-*Anthotium*-*Dampiera* (LAD) group, united by the presence of a cymo-paniculate inflorescence, connate anthers and a base chromosome number × = 9. In contrast, the remaining genera within the ‘*Goodenia* group’ had a fundamentally different vascularisation of the ovary, a thyrse or raceme-like inflorescence (Figs 2G, 3, 4), free anthers, and a base chromosome number of × = 7 or 8. These broad relationships were borne out in subsequent molecular studies (Jabaily et al. 2012; Gardner et al. 2016a) (Fig. 1). Carolin (1992a) rightly pointed out that *Brunonia* was clearly allied to the LAD group and perhaps should not be supported as a distinct family; however, Brunoniaceae was ultimately retained for his *Flora of Australia* treatment due to the adoption of the Cronquist (1981) classification system by the *Flora* at its inception (Kanis 1981). Within his ‘*Goodenia* group’, Carolin (1977) also determined that the monotypic south-west Western Australian genus *Diaspasis* was allied to *Scaevola* despite the presence of connate anthers and almost radially symmetrical flowers compared to the free anthers and fan-shaped flowers typical of the widely distributed *Scaevola* (Carolin 1992c; 1992d); a relationship subsequently confirmed through phylogenetic analyses (Jabaily et al. 2012). Finally, Carolin (1990; 1992e) concluded that *Coopernookia*, *Velleia*, *Verreauxia*, and *Pentaptilon* were allied to *Goodenia*, along with the four genera *Calogyne* R.Br., *Symphyobasis* K.Krause, *Neogoodenia* C.A.Gardner & A.S.George, and *Catosperma* Benth. that were later subsumed into an expanded *Goodenia*.

*Coopernookia* is the only genus in the family that shows the classic SW–SE Australian disjunction from the Nullarbor uplift around 8.8 to 0.5 million years ago (Jabaily et al. 2014), with three species endemic to the central and south west of the continent and three confined to eastern Australia (Carolin 1992b). Carolin (1967b) recognised this genus as distinct from the rest of his ‘*Goodenia* group’ as all species have a base chromosome number of × = 7 (rather than 8), stellate hairs on the stems and leaves, and ovoid, strophiolate seeds. The seed testa is also unique, with thickened, straight-sided cell walls that contain no mucilage in contrast to many species of *Goodenia* that have somewhat compressed seeds, where the epidermal cells are thickened towards the centre and thinner towards the margins and may contain mucilage that swells when wet (Carolin 1966). The flowers of *Coopernookia* also have retrorse barbulae inside the corolla that act as pollinator guides, reminiscent to those present in *Scaevola*. Indeed, Carolin (1967b) insightfully postulated that *Coopernookia* would have an intermediate position between *Scaevola* and *Goodenia*, which was later supported by molecular data as it was shown to be sister to *Goodenias.l.* (Gardner et al. 2016a).

**Figure 5.**
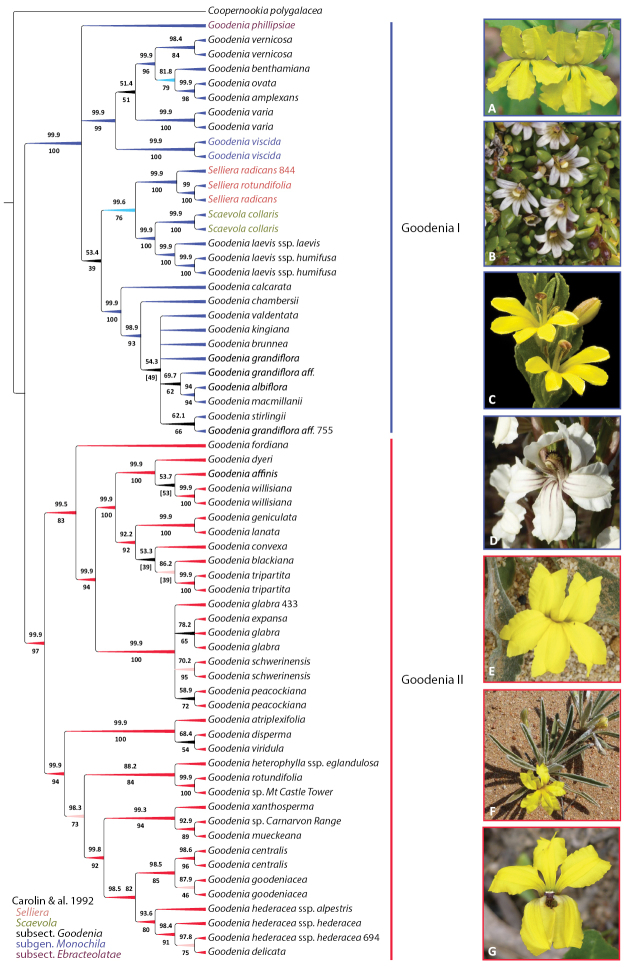
*Goodenia* Clade A phylogeny from combined nrDNA + cpDNA sequence data and exemplar taxa of major subclades. Topology is 50% majority rule cladogram from the partitioned Bayesian inference analysis. Support values above the branches are Bayesian posterior probabilities and below are maximum likelihood bootstrap values. Branch colour corresponds with support values and taxon colour corresponds to the taxonomic classification of Carolin et al. (1992). For updated taxonomy from this paper, see Tables 1, 2. Taxa represented by multiple accessions are distinguished by project code numbers as listed in Suppl. material 1. **A***G. ovata***B***Selliera radicans***C***G. viscida***D***G. calcarata*; **E***G. tripartita***F***G. willisiana***G***G. hederacea*. Images: J. Tann (**A, G**); R. Cumming (**B**); K.R. Thiele (**C**); Seeds of South Australia (**D, F**); K.A. Shepherd (**E**).

**Figure 6.**
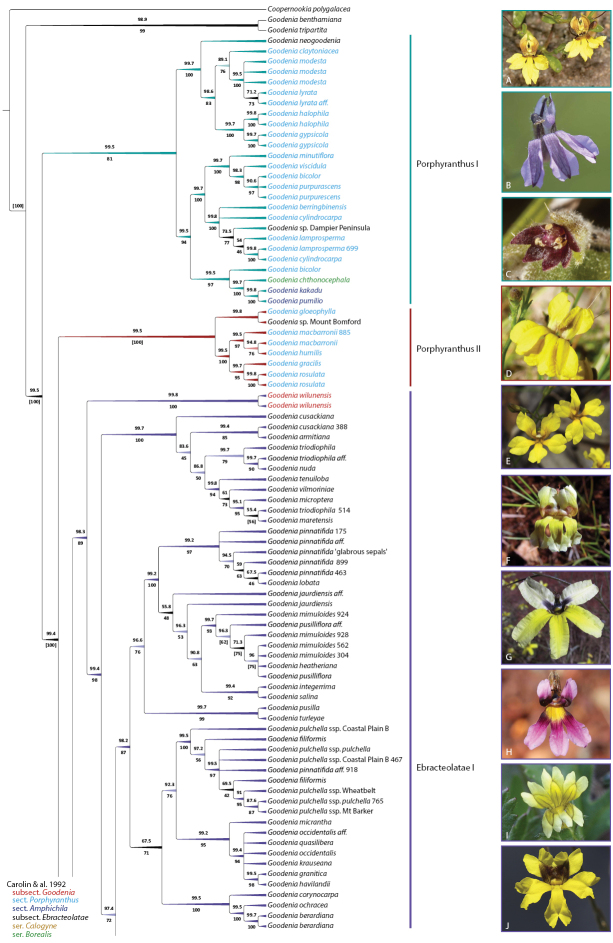
*Goodenia* Clade B_1 phylogeny from combined nrDNA + cpDNA sequence data and exemplar taxa of major subclades. Topology is 50% majority rule cladogram from the partitioned Bayesian inference analysis. Support values above the branches are Bayesian posterior probabilities and below are maximum likelihood bootstrap values. Branch colour corresponds with support values and taxon colour corresponds to the taxonomic classification of Carolin et al. (1992). For updated taxonomy from this paper, see Tables 1, 2. Taxa represented by multiple accessions are distinguished by project code numbers as listed in Suppl. material 1. **A***G. claytoniacea***B***G. purpurascens***C***G. pumilio***D***G. macbarronii***E***G. pinnatifida***F***G. nuda***G***G. tenuiloba***H***G. vilmoriniae***I***G. pusilliflora***J***G. occidentalis*. Images: F. & J. Hort (**A**); C. Nieminski (**B**); R.L. Barrett (**C**); N. Blair (**D**); K.A. Shepherd (**E, J**); A. Perkins (**F, G**); R. Fryer & J. Newland (**H**); A. Gardner (**I**).

**Figure 7.**
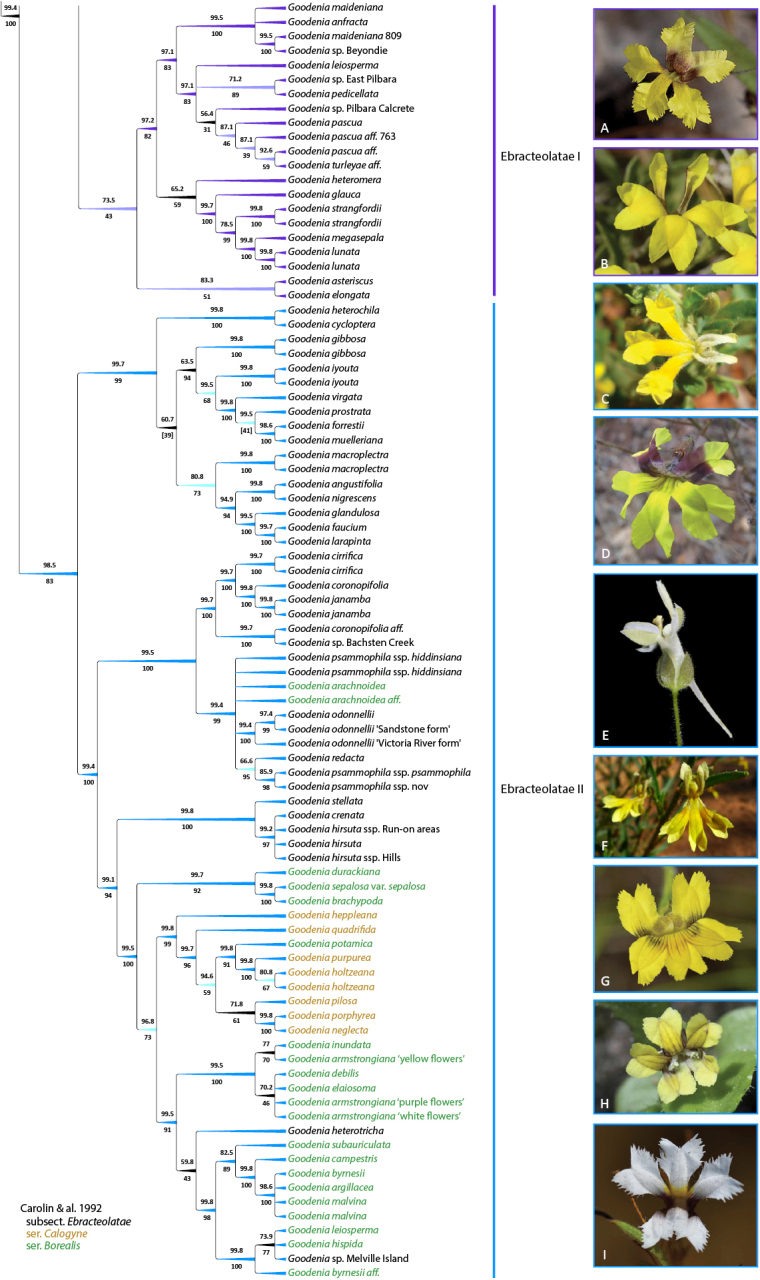
*Goodenia* Clade B_2 phylogeny from combined nrDNA + cpDNA sequence data and exemplar taxa of major subclades. Topology is 50% majority rule cladogram from the partitioned Bayesian inference analysis. Support values above the branches are Bayesian posterior probabilities and below are maximum likelihood bootstrap values. Branch colour corresponds with support values and taxon colour corresponds to the taxonomic classification of Carolin et al. (1992). For updated taxonomy from this paper, see Tables 1, 2. Taxa represented by multiple accessions are distinguished by project code numbers as listed in Suppl. material 1. **A***G. leiosperma***B***G. heteromera***C***G. heterochila***D***G. muelleriana***E***G. macroplectra***F***G. glandulosa***G***G. odonnellii***H***G. pilosa***I***G. armstrongiana* (White form). Images: C. Nieminski (**A, G–I**); Seeds of South Australia (**B, C, F**); A. Perkins (**D**); K.R. Thiele (**E**).

**Figure 8.**
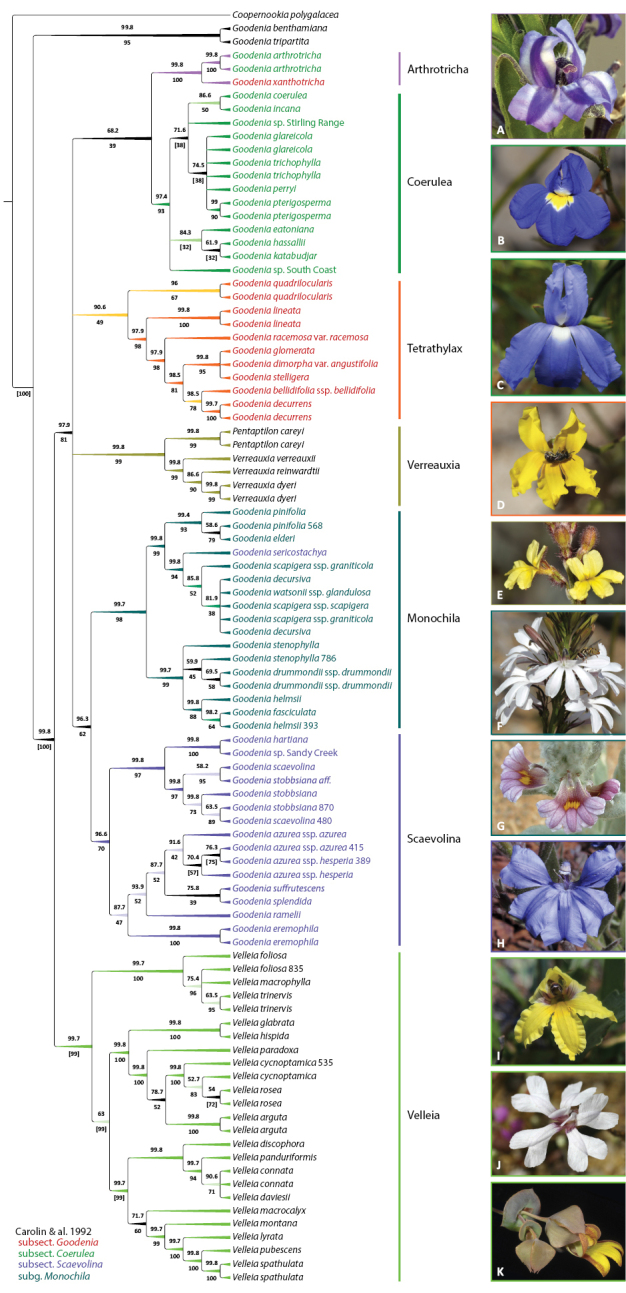
*Goodenia* Clade C phylogeny from combined nrDNA + cpDNA sequence data and exemplar taxa of major subclades. Topology is 50% majority rule cladogram from the partitioned Bayesian inference analysis. Support values above the branches are Bayesian posterior probabilities and below are maximum likelihood bootstrap values. Branch colour corresponds with support values and taxon colour corresponds to the taxonomic classification of Carolin et al. (1992). For updated taxonomy from this paper, see Tables 1, 2. Taxa represented by multiple accessions are distinguished by project code numbers as listed in Suppl. material 1. **A***G. xanthotricha***B***G. coerulea***C***G. hassallii***D***G. quadrilocularis***E***Verreauxia reinwardtii***F***G. helmsii***G***G. sericostachya***H***G. stobbsiana***I***Velleia paradoxa***J***V. rosea***K***V. connata*. Images: A. Crawford (**A**); F. & J. Hort (**B, E–G**); K.A. Shepherd (**C, D, I, J**); A. Perkins (**H**); K.R.Thiele (**K**).

*Goodenia* is the largest and most floristically diverse genus in Goodeniaceae with c. 220 species. Species are largely confined to Australia apart from a few representatives that extend northwards to New Guinea, Indonesia, Malaysia, Philippines, and China, with a single taxon also endemic to the Island of Java (Leenhouts 1957; Carolin 1992e; Hong and Howarth 2011). *Goodenia* are annual or perennial herbs or low shrubs that occupy a wide variety of habitats in almost every biome across the Australian continent. Many species have yellow, white or blue bilabiate flowers (Figs 2, 5–8), although there have been multiple independent floral symmetry shifts to a fan-shaped flower form (Gardner et al. 2016b). Fruit structure, seed coat surface patterns and appendages such as wings (Fig. 2F) are also important diagnostic characters for the genus (Carolin 1980). Recently, it was determined that the genus *Goodenia* had been lectotypified incorrectly as the first named species, *G. ramosissima* Sm., is in fact a species of *Scaevola* (≡ *S. ramosissima* (Sm.) K.Krause). Consequently, a proposal was put forward to conserve the name *Goodenia* using the conserved type *G. ovata* Sm. (Shepherd et al. 2017) (Fig. 2A), which was subsequently accepted (Applequist 2019). Carolin’s (1992e) infrageneric classification currently recognises two subgenera and various sections, subsections and series (Table 1).

**Table 1.** Revised classification of *Goodenias.l.* comparing the *Flora of Australia* treatment by Carolin (1992e) to an expanded *Goodenia* circumscribed herein that includes the genera *Selliera*, *Verreauxia*, *Pentaptilon* and *Velleia*, with the updated infrageneric classification and new authorities also provided.

Clade	Carolin et al. (1992)					Shepherd et al.		
	Genus	subgenus	section	subsection	series	Subgenus	Section	Subsection
Clade A	* Goodenia *	* Goodenia *	* Goodenia *	* Goodenia *		* Goodenia *	*Goodenia* [including *Selliera*]	
	* Goodenia *	* Goodenia *	* Goodenia *	* Goodenia *		* Goodenia *	*Rosulatae* (K.Krause) K.A.Sheph.	
Clade B	* Goodenia *	* Goodenia *	* Porphyranthus *			*Porphyranthus* (G.Don) K.A.Sheph.	* Porphyranthus *	
	* Goodenia *	* Goodenia *	* Amphichila *			*Porphyranthus* (G.Don) K.A.Sheph.	* Porphyranthus *	
	* Goodenia *	* Goodenia *	* Goodenia *	* Ebracteolatae *		*Porphyranthus* (G.Don) K.A.Sheph.	*Ebracteolatae* (K.Krause) K.A.Sheph.	
	* Goodenia *	* Goodenia *	* Goodenia *	* Borealis *	* Borealis *	*Porphyranthus* (G.Don) K.A.Sheph.	*Ebracteolatae* (K.Krause) K.A.Sheph.	
	* Goodenia *	* Goodenia *	* Goodenia *	* Borealis *	* Calogyne *	*Porphyranthus* (G.Don) K.A.Sheph.	*Ebracteolatae* (K.Krause) K.A.Sheph.	
Clade C	* Goodenia *	* Monochila *				* Monochila *	* Monochila *	*Monochila* (G.Don) K.A.Sheph.
	* Goodenia *	* Monochila *				* Monochila *	* Monochila *	*Infracta* K.A.Sheph.
	* Goodenia *	* Goodenia *	* Coeruleae *	* Coeruleae *		* Monochila *	* Coeruleae *	
	* Goodenia *	* Goodenia *	* Coeruleae *	* Scaevolina *		* Monochila *	*Scaevolina* (Carolin) K.A.Sheph.	
	* Goodenia *	*Goodenia p.p.*	*Goodenia p.p.*			* Monochila *	* Tetrathylax *	
	* Verreauxia *					* Monochila *	*Verreauxia* (Benth.) K.A.Sheph.	
	* Pentaptilon *					* Monochila *	*Verreauxia* (Benth.) K.A.Sheph.	
	* Velleia *		* Velleia *			* Monochila *	* Velleia *	
	* Velleia *		* Menoceras *			* Monochila *	* Velleia *	
	* Velleia *		* Euthales *			* Monochila *	* Velleia *	

*Selliera*, a genus of three fan-flowered species from Australia, New Zealand and Chile, was supported as distinct within Carolin’s (1977) ‘*Goodenia* group’. However, he later questioned its status (Carolin 1992f), noting that these species resembled members of Goodenia sect. Goodenia with fruits that show “a striking resemblance to that of *G. koningsbergeri* (Backer) Backer ex Bold. although somewhat smaller” (Carolin 1966), and he suggested that future work may determine that *Selliera* should be synonymised under *Goodenia*. This appears to be supported as a single representative of the genus, *Selliera radicans* Cav. (Fig. 2I), was shown to group with the unusual *Scaevola collaris* (Carolin 1992c) (Fig. 2B) and the fan-flowered *G. viscida* R.Br. (Fig. 5C) in *Goodenia* Clade A (Jabaily et al. 2012; Gardner et al. 2016a). Furthermore, *S. radicans* had been previously synonymised into *Goodenia* by Persoon (1805); however, subsequent workers had not taken up this proposed change.

*Verreauxia*, a small genus of three species from southwest Western Australia, is distinguished by unusual multi-cellular branched hairs and a unilocular ovary (Fig. 4 and Fig. 8E) that develops into an indehiscent, nut-like fruit with a single seed that (unusually) does not contain any mucilaginous cells in the seed coat testa (Carolin 1966, 1992i). Carolin (1977) also included this genus in his ‘*Goodenia* group’ along with the closely allied monotypic *Pentaptilon*, which has similar branched hairs but is distinguished by its uniquely winged ovary and fruit (Carolin 1992h). *Pentaptilon* together with *Verreauxia* formed a monophyletic group in molecular analyses within the morphologically variable Goodenia Clade C (Gardner et al. 2016a; Jabaily et al. 2018).

*Velleia*, the final genus in Carolin’s (1977) ‘*Goodenia* group’, currently includes 21 species endemic to Australia except *V. spathulata* R.Br., which is also found in Malaysia, western New Guinea and the Louisiade Archipelago (Leenhouts 1957; Carolin 1992g). Many species within this genus have a distinctive inflorescence structure comprised of expanded axillary dichasia (Fig. 3H). Another important diagnostic feature for *Velleia* is the presence of a predominantly superior ovary with the sepals, corolla and stamens usually adnate to the base (Carolin 1992g) (Fig. 2E). In contrast, the remaining Core Goodeniaceae generally have inferior ovaries, except *G. macroplectra* (F.Muell.) Carolin; a species in Goodenia subsect. Ebracteolatae (Jabaily et al. 2012) that has free sepals inferior to the ovary while the corolla is fused to the apex (Carolin 2007) (Fig. 7E). However, anatomical examination of *Velleia* ovary sections by Carolin (1959) revealed the floral parts are in fact fused to the ovary to a degree and the stamens are never fully hypogynous and in many species they appear epigynous (Jeanes 1999) (Fig. 4). Carolin (1977) stated that the flowers, fruits and seeds of *Velleia* are similar to those of various species of *Goodenia* and suggested that the morphology of the ovary was not a “reversion to an almost superior ovary but the vestiges of the former inferior condition are retained”. The infrageneric classification of *Velleia*, as currently recognised in the *Flora of Australia*, includes three sections (Carolin 1992g), based on the presence of three sepals (sect. Velleia) or five, which are either connate into a tube (sect. Euthales (R.Br.) Carolin) or free (sect. Menoceras R.Br.). While *Velleia* was supported as monophyletic in molecular analyses, it is placed sister to the remaining species in *Goodenia* Clade C (Gardner et al. 2016a; Jabaily et al. 2018).

### Inflorescence structure in *Goodenia* and allied genera

Genera within *Goodenias.l.* display a wide variation in floral form. Carolin (1967a) suggested the inflorescence structure was based on an open, polytelic, thyrsoid form with bracts that may be leaf-like (Fig. 2H) or reduced. The component axillary cymules of the ‘primitive’ thyrse may become reduced to form racemes and spikes (Fig. 3A), or the main axis may contract to form subumbels (Carolin et al. 1992) (Fig. 2C). Carolin (1967a) outlined nine reference ‘types’ that summarise the variation in the “Bauplan” across *Goodenias.l.* While some inflorescence forms correspond to various infrageneric groups, Carolin’s (1967a) survey of inflorescence structure was not comprehensive enough to extrapolate further. Therefore, a more complete survey was undertaken across the Core Goodeniaceae to determine if patterns in floral form are diagnostic and correspond to monophyletic groups recovered in our molecular analyses.

### Aims

Roger Carolin’s lifetime of work provides a sound framework to test hypotheses about evolutionary relationships in Goodeniaceae and allow for a re-examination of his generic concepts and infrageneric groups. The aim of this study is to build on our previous research (Jabaily et al. 2012; Gardner et al. 2016a; Jabaily et al. 2018) to produce well-sampled and well-resolved phylogenies combining both nrDNA (ITS) and cpDNA (*trnL-F*, *matK*) molecular markers. These updated phylogenies, in conjunction with a survey of inflorescence structure, will clarify our understanding of the systematic importance of these features to characterise subclades within *Goodenias.l.* The time has now come to also update Carolin’s *Flora of Australia* classification to reflect these findings and to formally name and describe monophyletic clades as infrageneric groups in *Goodenias.l.* in order to ensure nomenclatural stability going forward.

## Methods

### Taxon sampling

Our study includes over 95% of described species within *Goodenias.l.* (Suppl. material 1). This paper includes sequences of *trnL-F* and *matK* from Jabaily et al. (2012), *trnL-F*, *matK*, and nrITS samples from Gardner et al. (2016b) with the majority of accessions new to this study. In some instances, multiple accessions of a taxon were included, including some subspecific taxa, to test for monophyly. A number of informal taxa or phrase-named taxa have also been included such as *Goodenia* sp. Dampier Peninsula (B.J. Carter 675) (Western Australian Herbarium 1998-; Council of Heads of Australasian Herbaria 2006-) to test the genetic uniqueness of these taxa and confirm allied species. Initially, a dataset with all taxa was aligned and analysed, but the backbone relationships between Goodenia Clade A, Clade B, and Clade C were unresolved, as expected from our prior work with these loci. Datasets were then created and analysed separately for all taxon sets (A, B, C), following following Jabaily et al. (2012) and Gardner et al. (2016a). Separating the total dataset by clade allowed for more precise alignment across taxa, particularly within *trnL-F*. As the genus *Coopernookia* was confirmed as sister to *Goodenias.l.* (Gardner et al. 2016a), *Coopernookia polygalacea* (de Vriese) Carolin was used as the outgroup for all three taxon sets; within Clade B and Clade C, two accessions from Clade A (*Goodenia benthamiana* Carolin and *G. tripartita* Carolin) were included as additional outgroups.

### Sequencing and Phylogenetic inference

Molecular sequencing primers and protocols follows Gardner et al. (2016a). Sequencing was completed by Macrogen (Seoul, South Korea). Individual loci were aligned in Geneious v. 11.0.2 (Kearse et al. 2012) using the Geneious tree building algorithm, with subsequent manual correction. For all taxon sets (A, B, C), three separate alignments were made for the chloroplast loci (*matK* and *trnL-F*), nuclear ribosomal locus (nrITS), and all data combined (*matK*, *trnL-F*, and nrITS). For each taxon set, individual loci were analysed for models of molecular evolution with Akaike information criterion (AIC) implemented in jModelTest2 (Guindon and Gascuel 2003; Darriba et al. 2012), implemented in CIPRES Science Gateway (Phylo.org; Miller et al. 2010). For the nrITS dataset of Clade B, the model selected was SYM + G + I. For the nrITS and cpDNA datasets of Clade A, the model selected was GTR + G. For the nrITS and cpDNA datasets of Clade C and the cpDNA dataset of Clade B, the model selected was GTR + G +I.

Bayesian phylogenetic analyses using MrBayes 3.2.2 (Ronquist et al. 2012) were conducted in CIPRES Science Gateway. For individual datasets each locus varied independently under the parameters specified by the individual model of molecular evolution. For each Bayesian analysis, two runs were conducted, each with three heated and one cold chain and uniform priors. The heated chain temperature was adjusted to ensure adequate mixing. Each analysis was set to run for up to 100 million generations, autoclosing when the standard deviation of split frequencies reached 0.01. Trees were sampled every 10000 generations, and 25% was discarded as burn-in. The adequacy of each analysis was completed by ensuring effective sample size >100, potential scale reduction factor of ~1.0 for all parameters, and acceptance rates of swaps between adjacent changes was between 0.1–0.7 in Tracer 1.6 (Rambaut et al. 2014). Majority rule consensus trees with posterior probabilities were generated in Geneious.

Maximum likelihood analyses using RAxML v. 8.0 (Stamatakis 2014) were conducted at the high-performance computing cluster (HiPerGator) at the University of Florida using the optimal models of molecular evolution for each dataset as discussed with 1000 bootstrap replications, summarized onto the best ML tree.

### Taxonomy and morphology

Typification, synonymy and taxonomy largely follow the *Flora of Australia* treatment (Carolin et al. 1992) and/or the Australian Plant Name Index (https://biodiversity.org.au/nsl/services/APNI). Field work was conducted over several years in southern Western Australia facilitating the collection of fresh samples for DNA sequencing and examination of plants *in situ*. Types and specimens at various herbaria or on loan (AD, BRI, CANB, CGG, DNA, K, LD, MEL, PERTH, W) were also critically examined for the morphological survey of inflorescence structure and for lectotypifications. Further material was viewed using Global Plants (http://plants.jstor.org/) and the Museum National d’Histoire Naturelle online database (https://science.mnhn.fr/institution/mnhn/search) (indicated by “image!” in the citation). Images of seeds of various species were viewed on the Seeds of South Australia website (https://spapps.environment.sa.gov.au/seedsofsa/). Non-Australian species of *Selliera* were assessed using online images available through the *Flora of New Zealand* (http://www.nzflora.info/search.html).

## Results

### Phylogenetic inference

The cpDNA and nrITS topologies were highly congruent for each taxon set representing Clades A, B and C and no taxon moved between named clades in the nrDNA, cpDNA and combined analyses. For each taxon set, both the chloroplast and nrITS trees (Suppl. materials 2–7) and original alignments (Suppl. materials 8–12) are available. Further, there were no substantial conflicting positions of strongly supported taxa, except where noted below.

Sixty-five accessions were included in the Clade A dataset, representing 50 named taxa (species, subspecies) and four unnamed taxa. Twenty-five of these were not included in previous studies, a 50% increase in taxon coverage. Clade A, representing the majority of species in subsect. Goodenia, resolves into two well-supported subclades (Goodenia I and II) with roughly similar numbers of taxa (Fig. 5). The backbone of subclade Goodenia I was poorly supported, with the position of *G. phillipsiae* Carolin (a species previously included in subsect. Ebracteolatae) differing between datasets. Similarly, accessions of *Selliera* placed in slightly different subclades. In the nrITS analysis, species of *Selliera* resolve as sister to *G. viscida* (previously included in subg. Monochila), several clades removed from *Scaevola collaris* and *G. laevis* Benth.; however, they are placed sister to these species in the cpDNA and combined analyses (Fig. 5). Goodenia II is congruent between datasets and resolves into two subclades. These were congruent between datasets except for a weakly supported subgroup comprising *G. atriplexifolia* A.E.Holland & T.P.Boyle, *G. disperma* F.Muell. and *G. viridula* Carolin that was recovered in the nrITS dataset but not retained in the cpDNA or combined data analyses.

The Clade B dataset comprised 175 accessions of 132 taxa (species, subspecies, and unnamed spp.) including 26 unnamed species. Seventy-seven taxa are newly included in this study, representing 58% of our sampling. Clade B comprises well-supported subclades (Porphyranthus I and II) of sect. Porphyranthus that are successively sister to subsect. Ebracteolatae, which resolves into strongly supported subclades Ebracteolatae I and II (Figs 6, 7). Taxon composition of these subclades and relative support values are congruent between datasets. In addition to including representatives of Carolin’s sect. Porphyranthus, the Porphyranthus I clade also comprises two representatives *G. kakadu* Carolin and *G. pumilio* R.Br. from sect. Amphichila; a small section of diminutive species found in damp habitats in Northern Australia with the latter species also extending to New Guinea. Our analyses show that *G. chthonocephala* Carolin, a poorly known and unusual cushion-like plant previously included in ser. Borealis Carolin, is also allied to these two species (Fig. 6). Further, *G. neogoodenia* Carolin, an atypical species currently included in subsect. Ebracteolatae, is allied to another group of northern Australian species in the Porphyranthus I clade. The remaining representatives of Carolin’s (1992e) ser. Borealis are included in the Ebracteolatae II clade, intermingled with species previously included in ser. Calogyne (Fig. 7), while *G. wilunensis* Carolin (subsect. Goodenia), is sister to the Ebracteolatae I clade (Fig. 6).

Clade C represents the most morphologically diverse group. Analyses included 92 accessions representing 67 taxa (with 4 being unnamed), a 31% increase in the number of species previously sampled across this clade. Seven individual subclades were well supported: a small group in sect. Goodenia, subsections *Scaevolina* and *Coerulea*, subg. Monochila and the genera *Velleia*, *Verreauxia* and *Pentaptilon* (Fig. 8). However, the relationships between clades remains unclear. In the combined analysis, subg. Monochila and subsect. Scaevolina were supported as sister, but this relationship was not found in the individual cpDNA and nrDNA trees. Similarly, *G. xanthotricha* de Vriese and *G. arthrotricha* Benth. were weakly supported as sister to subsect. Coeruleae in the combined analysis only. Surprisingly, *G. quadrilocularis* R.Br. is supported as sister to *Velleia* on the nrITS tree while it was sister to the subset of species from subsect. Goodenia in the cpDNA and combined analyses.

### Inflorescence morphology

Carolin (1967a) originally classified the various inflorescence structures evident in *Goodenias.l.* into nine different types, based on a relatively limited number of species. A survey of inflorescence morphology across *Goodenias.l.* was undertaken here, utilising published information, images and herbarium specimens, to confirm key diagnostic characters such as the position and insertion of leaves and bracts, the presence or absence of bracteoles, and overall inflorescence form (Table 2). It should be noted that it is not always easy to distinguish leafy bracts from cauline leaves or between bracts and bracteoles in this group. For example, Albrecht (2002) observed that while *Goodenia halophila* Albr. and *G. cylindrocarpa* Albr. have structures subtending the flowers that look like bracteoles, axillary buds are sometimes present. For that reason, he decided to follow the classification of Briggs and Johnson (1979) and used the term “opposite or sub-opposite bracts” rather than bracteoles. While more accurate in some respects, this terminology is not entirely satisfactory and subsequent authors have continued to use the term bracts for reduced cauline leaves that subtend flowers, and appendages on the flower stalk, when present, are termed bracteoles (Holland and Boyle 2002; Cowie 2005; Pellow and Porter 2005; Sage and Shepherd 2007; Lang 2014). This survey also follows Carolin’s concepts for floral structure; however, a more comprehensive evo-devo study of floral development that considers the genetic mechanisms that control branching patterns of the floral-axis (i.e. inflorescences), would greatly improve our understanding of these complex structures.

**Table 2.** An updated linear sequence and classification for *Goodenias.l.* including phylogenetic position (NS = species not sequenced) and a summary of morphological characters such as leaf position, inflorescence form and type (as characterised in Figure 2), the presence of leafy (L), bractose (N) or disc-like (D) bracts, and presence (1) or absence (0) of bracteoles. Authorities for most taxa are available in APNI (https://biodiversity.org.au/nsl/services/apni). *Species with an uncertain placement within Clade C.

New linear sequence	Flora of Australia sequence	Current name	Shepherd et al. name	Shepherd et al. classification	Phylogenetic clade	Leaves	Inflorescence form	Inflorescence type	Bracts leafy (L) or bracteose (N)	Bracteoles absent (0); present (1)
1	111	*Goodenia phillipsiae*		subg. Goodenia sect. Goodenia	Goodenia ss I_I	cauline	thyrse	A	L	1
2	45	*Goodenia vernicosa*		subg. Goodenia sect. Goodenia	Goodenia ss I_I	cauline	raceme; thyrse	A	L	1
3	50	*Goodenia benthamiana*		subg. Goodenia sect. Goodenia	Goodenia ss I_I	cauline	raceme	A	L	1
4	43	*Goodenia ovata*		subg. Goodenia sect. Goodenia	Goodenia ss I_I	cauline	raceme; thyrse	A	L	1
5	49	*Goodenia amplexans*		subg. Goodenia sect. Goodenia	Goodenia ss I_I	cauline	raceme	A	L	1
6	44	*Goodenia varia*		subg. Goodenia sect. Goodenia	Goodenia ss I_I	cauline	raceme; thyrse	A	L	1
7	10	*Goodenia viscida*		subg. Goodenia sect. Goodenia	Goodenia ss I_I	cauline	spike	A	L	1
8		*Selliera radicans*	*Goodenia radicans*	subg. Goodenia sect. Goodenia	Goodenia ss I_I	cauline	raceme; solitary	A	L	1
9		*Selliera rotundifolia*	*Goodenia heenanii*	subg. Goodenia sect. Goodenia	Goodenia ss I_I	cauline	raceme; solitary	A	L	?
10		*Scaevola collaris*	*Goodenia collaris*	subg. Goodenia sect. Goodenia	Goodenia ss I_I	cauline	raceme; solitary	A	L	1
11	93	Goodenia laevis subsp. laevis		subg. Goodenia sect. Goodenia	Goodenia ss I_I	cauline	raceme; thyrse	A	L	1
12		Goodenia laevis subsp. humifusa		subg. Goodenia sect. Goodenia	Goodenia ss I_I	cauline	raceme; thyrse	A	L	1
13		*Velleia exigua*	*Goodenia exigua*	subg. Goodenia sect. Goodenia	NS	Basal; cauline	solitary; raceme	A; C	L	1
14		*Goodenia koningsbergeri*		subg. Goodenia sect. Goodenia	NS	cauline	raceme	A	L	1
15	56	*Goodenia calcarata*		subg. Goodenia sect. Goodenia	Goodenia ss I_II	cauline	raceme	A	N, L	1
16	47	*Goodenia chambersii*		subg. Goodenia sect. Goodenia	Goodenia ss I_II	cauline	raceme; thyrse	A	L	1
17		*Goodenia valdentata*		subg. Goodenia sect. Goodenia	Goodenia ss I_II	cauline	raceme; thyrse	A	L	1
18	48	*Goodenia kingiana*		subg. Goodenia sect. Goodenia	Goodenia ss I_II	cauline	raceme; thyrse	A	L	1
19	53	*Goodenia brunnea*		subg. Goodenia sect. Goodenia	Goodenia ss I_II	cauline	raceme; thyrse	A	L	1
20	54	*Goodenia saccata*		subg. Goodenia sect. Goodenia	NS	cauline	raceme; thyrse	A	L	1
21	46	*Goodenia grandiflora*		subg. Goodenia sect. Goodenia	Goodenia ss I_II	cauline	raceme; thyrse	A	L	1
22	51	*Goodenia albiflora*		subg. Goodenia sect. Goodenia	Goodenia ss I_II	cauline	raceme	A	L	1
23	57	*Goodenia macmillanii*		subg. Goodenia sect. Goodenia	Goodenia ss I_II	cauline	raceme	A	L	1
24	55	*Goodenia stirlingii*		subg. Goodenia sect. Goodenia	Goodenia ss I_II	cauline	raceme	A	L	1
25	58	*Goodenia fordiana*		subg. Goodenia sect. Rosulatae	Goodenia ss II_I	basal	raceme; solitary	B; C	L	1
26	88	*Goodenia dyeri*		subg. Goodenia sect. Rosulatae	Goodenia ss II_I	basal	solitary	C	L	1
27	83	*Goodenia affinis*		subg. Goodenia sect. Rosulatae	Goodenia ss II_I	basal	raceme; solitary	B; C	L	1
28	86	*Goodenia willisiana*		subg. Goodenia sect. Rosulatae	Goodenia ss II_I	basal	raceme; solitary	B; C	L	1
29	87	*Goodenia robusta*		subg. Goodenia sect. Rosulatae	NS	basal; cauline	raceme	B	L	1
30	81	*Goodenia geniculata*		subg. Goodenia sect. Rosulatae	Goodenia ss II_I	basal; cauline	raceme; solitary	B; C	L	1
31	82	*Goodenia lanata*		subg. Goodenia sect. Rosulatae	Goodenia ss II_I	basal; cauline	raceme	B	L	1
32	84	*Goodenia convexa*		subg. Goodenia sect. Rosulatae	Goodenia ss II_I	basal	raceme; solitary	B; C	L	1
33	80	*Goodenia blackiana*		subg. Goodenia sect. Rosulatae	Goodenia ss II_I	basal	raceme; solitary	B; C	L	1
34	85	*Goodenia tripartita*		subg. Goodenia sect. Rosulatae	Goodenia ss II_I	basal	raceme; solitary	B; C	L	1
35	90	*Goodenia glabra*		subg. Goodenia sect. Rosulatae	Goodenia ss II_I	basal; cauline	raceme	B	L	1
36		*Goodenia expansa*		subg. Goodenia sect. Rosulatae	Goodenia ss II_I	basal; cauline	raceme	B	L	1
37	91	*Goodenia peacockiana*		subg. Goodenia sect. Rosulatae	Goodenia ss II_I	basal; cauline	raceme	B	L	1
38	92	*Goodenia schwerinensis*		subg. Goodenia sect. Rosulatae	Goodenia ss II_I	basal; cauline	raceme	B	L	1
39		*Goodenia atriplexifolia*		subg. Goodenia sect. Rosulatae	Goodenia ss II_II	cauline	spike	A	L	1
40	68	*Goodenia disperma*		subg. Goodenia sect. Rosulatae	Goodenia ss II_II	cauline	raceme	A	L	1
41	69	*Goodenia viridula*		subg. Goodenia sect. Rosulatae	Goodenia ss II_II	cauline	spike	A	L	1
42	70	*Goodenia stephensonii*		subg. Goodenia sect. Rosulatae	NS	cauline	raceme	A	L	1
43	71a	Goodenia heterophylla subsp. heterophylla		subg. Goodenia sect. Rosulatae	NS	cauline	raceme	A	L	1
44	71b	Goodenia heterophylla subsp. egalndulosa		subg. Goodenia sect. Rosulatae	Goodenia ss II_II	cauline	raceme	A; B	L	1
45	71c	Goodenia heterophylla subsp. teucriifolia		subg. Goodenia sect. Rosulatae	NS	cauline	raceme	A; B	L	1
46	71d	Goodenia heterophylla subsp. montana		subg. Goodenia sect. Rosulatae	NS	cauline	raceme	A	L	1
47	72	*Goodenia rotundifolia*		subg. Goodenia sect. Rosulatae	Goodenia ss II_II	cauline	raceme	A	L	1
48	73	*Goodenia arenicola*		subg. Goodenia sect. Rosulatae	NS	cauline	solitary	A	?N, L	1
49	76	*Goodenia xanthosperma*		subg. Goodenia sect. Rosulatae	Goodenia ss II_II	basal; cauline	raceme	B	L	1
50	79	*Goodenia rupestris*		subg. Goodenia sect. Rosulatae	NS	basal; cauline	raceme	B	L	1
51	94	*Goodenia mueckeana*		subg. Goodenia sect. Rosulatae	Goodenia ss II_II	basal; cauline	raceme; thyrse	A, B	L	1
52	77	*Goodenia centralis*		subg. Goodenia sect. Rosulatae	Goodenia ss II_II	basal; cauline	raceme	B	L	1
53	78	*Goodenia goodeniacea*		subg. Goodenia sect. Rosulatae	Goodenia ss II_II	basal; cauline	thyrse	B	L	1
54	75	Goodenia hederacea subsp. hederacea		subg. Goodenia sect. Rosulatae	Goodenia ss II_II	basal; cauline	raceme; thyrse	B	L	1
55	75a	Goodenia hederacea subsp. alpestris		subg. Goodenia sect. Rosulatae	Goodenia ss II_II	basal; cauline	raceme	B	L	1
56	74	*Goodenia delicata*		subg. Goodenia sect. Rosulatae	Goodenia ss II_II	basal; cauline	raceme	B	L	1
57	153	*Goodenia neogoodenia*		subg. Porphyranthus sect. Porphyranthus	Porphyranthus I	basal; cauline	raceme	E; F	N; L	0
58	25	*Goodenia claytoniacea*		subg. Porphyranthus sect. Porphyranthus	Porphyranthus I	basal; cauline	raceme; thyrse; panicle-like	B	L	1
59	24	*Goodenia modesta*		subg. Porphyranthus sect. Porphyranthus	Porphyranthus I	basal; cauline	thyrse; panicle-like	B	L	1
60	23	*Goodenia lyrata*		subg. Porphyranthus sect. Porphyranthus	Porphyranthus I	basal; cauline	raceme	B	L	1
61		*Goodenia halophila*		subg. Porphyranthus sect. Porphyranthus	Porphyranthus I	basal; cauline	panicle-like	B, E	L, N	1
62		*Goodenia gypsicola*		subg. Porphyranthus sect. Porphyranthus	Porphyranthus I	basal	panicle-like	B, E	L, N	1
63	19	*Goodenia minutiflora*		subg. Porphyranthus sect. Porphyranthus	Porphyranthus I	basal	raceme; thyrse	E	N	1
64	20	*Goodenia viscidula*		subg. Porphyranthus sect. Porphyranthus	Porphyranthus I	basal	thyrse; panicle-like	B, E	L, N	1
65	16	*Goodenia bicolor*		subg. Porphyranthus sect. Porphyranthus	Porphyranthus I	basal; cauline	raceme; thyrse	E	N	1
66	18	*Goodenia purpurascens*		subg. Porphyranthus sect. Porphyranthus	Porphyranthus I	basal	thyrse; panicle-like	E	N	1
67	22	*Goodenia berringbinensis*		subg. Porphyranthus sect. Porphyranthus	Porphyranthus I	basal	raceme; thyrse	E	N	1
68	15	*Goodenia lamprosperma*		subg. Porphyranthus sect. Porphyranthus	Porphyranthus I	basal; cauline	raceme; thyrse	B	L	1
69		*Goodenia corralina*		subg. Porphyranthus sect. Porphyranthus	NS	basal; cauline	raceme; thyrse	B, E	L, N	1
70		*Goodenia cylindrocarpa*		subg. Porphyranthus sect. Porphyranthus	Porphyranthus I	basal	panicle-like	B, E	L, N	1
71		*Goodenia nocoleche*		subg. Porphyranthus sect. Porphyranthus	NS	basal; cauline	raceme	B, E	L; N	1
72	165	*Goodenia chthonocephala*		subg. Porphyranthus sect. Porphyranthus	Porphyranthus I	basal	solitary	D	L	0
73	178	*Goodenia kakadu*		subg. Porphyranthus sect. Porphyranthus	Porphyranthus I	basal; cauline	solitary; raceme	D; F	L	0
74		*Goodenia oenpelliensis*		subg. Porphyranthus sect. Porphyranthus	NS	basal; cauline	solitary; raceme	D; F	L	0
75	177	*Goodenia pumilio*		subg. Porphyranthus sect. Porphyranthus	Porphyranthus I	basal; cauline	solitary	D	L	0
76		*Goodenia cravenii*		subg. Porphyranthus sect. Porphyranthus	NS	basal	solitary; raceme	D; F	L	0
77	17	*Goodenia gloeophylla*		subg. Porphyranthus sect. Porphyranthus	Porphyranthus II	basal; cauline	raceme	B	L	1
78	13	*Goodenia macbarronii*		subg. Porphyranthus sect. Porphyranthus	Porphyranthus II	basal (mostly); cauline	raceme; thyrse	E	N	1
79	12	*Goodenia paniculata*		subg. Porphyranthus sect. Porphyranthus	NS	basal	raceme; thyrse or panicle-like	B, E	L, N	1
80	14	*Goodenia humilis*		subg. Porphyranthus sect. Porphyranthus	Porphyranthus II	basal	raceme	E	N	1
81	11	*Goodenia gracilis*		subg. Porphyranthus sect. Porphyranthus	Porphyranthus II	basal; cauline	raceme; thyrse	E	N	1
82		*Goodenia rosulata*		subg. Porphyranthus sect. Porphyranthus	Porphyranthus II	basal; cauline	thyrse; panicle-like	E	N	1
83	89	*Goodenia wilunensis*		subg. Porphyranthus sect. Ebracteolatae	Ebracteolatae I	basal; cauline	raceme	B	L	1
84	149	*Goodenia cusackiana*		subg. Porphyranthus sect. Ebracteolatae	Ebracteolatae I	basal	raceme	F	N	0
85	139	*Goodenia armitiana*		subg. Porphyranthus sect. Ebracteolatae	Ebracteolatae I	basal; cauline	raceme; subumbel	F	L	0
86	141	*Goodenia triodiophila*		subg. Porphyranthus sect. Ebracteolatae	Ebracteolatae I	cauline	raceme; subumbel	F	L	0
87	143	*Goodenia nuda*		subg. Porphyranthus sect. Ebracteolatae	Ebracteolatae I	basal	raceme; subumbel	F	L	0
88	144	*Goodenia pallida*		subg. Porphyranthus sect. Ebracteolatae	NS	basal; cauline	raceme; subumbel	F	L	0
89	148	*Goodenia tenuiloba*		subg. Porphyranthus sect. Ebracteolatae	Ebracteolatae I	basal; cauline	raceme	F	L	0
90	150	*Goodenia vilmorinae*		subg. Porphyranthus sect. Ebracteolatae	Ebracteolatae I	basal; cauline	raceme; subumbel	F	L	0
91	142	*Goodenia microptera*		subg. Porphyranthus sect. Ebracteolatae	Ebracteolatae I	basal; cauline	raceme; subumbel	F	L	0
92		*Goodenia maretensis*		subg. Porphyranthus sect. Ebracteolatae	Ebracteolatae I	basal; cauline	raceme; subumbel	F	L	0
93	110	*Goodenia pinnatifida*		subg. Porphyranthus sect. Ebracteolatae	Ebracteolatae I	basal	raceme; subumbel	F	L	0
94	120	*Goodenia lobata*		subg. Porphyranthus sect. Ebracteolatae	Ebracteolatae I	basal; cauline	raceme; subumbel	F	L	0
95	121	*Goodenia salmoniana*		subg. Porphyranthus sect. Ebracteolatae	NS	?basal; cauline	raceme	F	L	0
96		*Goodenia jaurdiensis*		subg. Porphyranthus sect. Ebracteolatae	Ebracteolatae I	basal	raceme	F	L	0
97	115	*Goodenia mimuloides*		subg. Porphyranthus sect. Ebracteolatae	Ebracteolatae I	basal	raceme	F	L	0
98		*Goodenia heatheriana*		subg. Porphyranthus sect. Ebracteolatae	Ebracteolatae I	basal	raceme	F	L	0
99	109	*Goodenia pusilliflora*		subg. Porphyranthus sect. Ebracteolatae	Ebracteolatae I	basal	raceme	F	L	0
100	107	*Goodenia integerrima*		subg. Porphyranthus sect. Ebracteolatae	Ebracteolatae I	basal; cauline	subumbel	F	L	0
101		*Goodenia salina*		subg. Porphyranthus sect. Ebracteolatae	Ebracteolatae I	basal	raceme; subumbel	F	L	0
102	102	*Goodenia pusilla*		subg. Porphyranthus sect. Ebracteolatae	Ebracteolatae I	basal	raceme	F	L	0
103		*Goodenia turleyae*		subg. Porphyranthus sect. Ebracteolatae	Ebracteolatae I	basal	raceme	F	L	0
104	123	*Goodenia filiformis*		subg. Porphyranthus sect. Ebracteolatae	Ebracteolatae I	basal	raceme; subumbel	F	L	0
105	122	*Goodenia pulchella*		subg. Porphyranthus sect. Ebracteolatae	Ebracteolatae I	basal	raceme; subumbel	F	L	0
106	128	*Goodenia micrantha*		subg. Porphyranthus sect. Ebracteolatae	Ebracteolatae I	basal; cauline	raceme	F	L	0
107	125	*Goodenia quasilibera*		subg. Porphyranthus sect. Ebracteolatae	Ebracteolatae I	basal	raceme; subumbel	F	L	0
108	124	*Goodenia concinna*		subg. Porphyranthus sect. Ebracteolatae	NS	basal	raceme; subumbel	F	L	0
109	126	*Goodenia occidentalis*		subg. Porphyranthus sect. Ebracteolatae	Ebracteolatae I	basal	raceme; subumbel	F	L	0
110	127	*Goodenia krauseana*		subg. Porphyranthus sect. Ebracteolatae	Ebracteolatae I	basal	subumbel	F	L	0
111		*Goodenia granitica*		subg. Porphyranthus sect. Ebracteolatae	Ebracteolatae I	basal; cauline	raceme	F	L	0
112	129	*Goodenia havilandii*		subg. Porphyranthus sect. Ebracteolatae	Ebracteolatae I	basal; cauline	raceme	F	L	0
113	105	*Goodenia corynocarpa*		subg. Porphyranthus sect. Ebracteolatae	Ebracteolatae I	basal; cauline	raceme; subumbel	F	L	0
114	114	*Goodenia ochracea*		subg. Porphyranthus sect. Ebracteolatae	Ebracteolatae I	basal	raceme	F	L	0
115	113	*Goodenia berardiana*		subg. Porphyranthus sect. Ebracteolatae	Ebracteolatae I	basal; cauline	raceme; subumbel	F	L	0
116	104	*Goodenia maideniana*		subg. Porphyranthus sect. Ebracteolatae	Ebracteolatae I	basal; cauline	raceme; subumbel	F	L	0
117	103	*Goodenia anfracta*		subg. Porphyranthus sect. Ebracteolatae	Ebracteolatae I	basal; cauline	raceme; subumbel	F	L	0
118		*Goodenia pedicellata*		subg. Porphyranthus sect. Ebracteolatae	Ebracteolatae I	basal	solitary; raceme	D; F	L	0
119	100	*Goodenia pascua*		subg. Porphyranthus sect. Ebracteolatae	Ebracteolatae I	basal; cauline	raceme; subumbel	F	L	0
120	101	*Goodenia heteromera*		subg. Porphyranthus sect. Ebracteolatae	Ebracteolatae I	basal	subumbel; solitary	F	L	0
121	97	*Goodenia glauca*		subg. Porphyranthus sect. Ebracteolatae	Ebracteolatae I	basal; cauline	raceme	B; F	L	1; 0
122	98	*Goodenia fascicularis*		subg. Porphyranthus sect. Ebracteolatae	NS	basal; cauline	raceme; subumbel	F	L	0
123	108	*Goodenia strangfordii*		subg. Porphyranthus sect. Ebracteolatae	Ebracteolatae I	cauline	raceme; subumbel	F	L	0
124	118	*Goodenia megasepala*		subg. Porphyranthus sect. Ebracteolatae	Ebracteolatae I	basal; cauline	raceme; subumbel	F	L	0
125	99	*Goodenia lunata*		subg. Porphyranthus sect. Ebracteolatae	Ebracteolatae I	basal	raceme; subumbel	F	L	0
126		*Goodenia asteriscus*		subg. Porphyranthus sect. Ebracteolatae	Ebracteolatae I	basal	subumbel	F	L	0
127	117	*Goodenia elongata*		subg. Porphyranthus sect. Ebracteolatae	Ebracteolatae I	basal; cauline	raceme	F	L	0
128	132	*Goodenia heterochila*		subg. Porphyranthus sect. Ebracteolatae	Ebracteolatae II	basal; cauline	raceme	B; F	L	1; 0
129	131	*Goodenia cycloptera*		subg. Porphyranthus sect. Ebracteolatae	Ebracteolatae II	basal; cauline	raceme; subumbel	F	L	0
130	112	*Goodenia gibbosa*		subg. Porphyranthus sect. Ebracteolatae	Ebracteolatae II	basal; cauline	raceme; subumbel	F	L	0
131	119	*Goodenia iyouta*		subg. Porphyranthus sect. Ebracteolatae	Ebracteolatae II	cauline	raceme	F	L	0
132	140	*Goodenia virgata*		subg. Porphyranthus sect. Ebracteolatae	Ebracteolatae II	basal; cauline	raceme; subumbel	F	L	0
133		*Goodenia effusa*		subg. Porphyranthus sect. Ebracteolatae	NS	basal; cauline	raceme; subumbel	F	L	0
134	145	*Goodenia prostrata*		subg. Porphyranthus sect. Ebracteolatae	Ebracteolatae II	basal	raceme; subumbel	F	L	0
135	147	*Goodenia forrestii*		subg. Porphyranthus sect. Ebracteolatae	Ebracteolatae II	cauline	raceme	F	L	0
136	146	*Goodenia muelleriana*		subg. Porphyranthus sect. Ebracteolatae	Ebracteolatae II	basal; cauline	raceme; subumbel	F	L	0
137	116	*Goodenia macroplectra*		subg. Porphyranthus sect. Ebracteolatae	Ebracteolatae II	basal; cauline	raceme; subumbel	F	L	0
138	96	*Goodenia angustifolia*		subg. Porphyranthus sect. Ebracteolatae	Ebracteolatae II	basal; cauline	raceme	B, F	L	0, 1
139	95	*Goodenia nigrescens*		subg. Porphyranthus sect. Ebracteolatae	Ebracteolatae II	basal; cauline	raceme	B	L	1
140	133	*Goodenia glandulosa*		subg. Porphyranthus sect. Ebracteolatae	Ebracteolatae II	cauline	raceme	F	L	0
141	135	*Goodenia faucium*		subg. Porphyranthus sect. Ebracteolatae	Ebracteolatae II	cauline	raceme	F	L	0
142	134	*Goodenia larapinta*		subg. Porphyranthus sect. Ebracteolatae	Ebracteolatae II	basal; cauline	raceme	F	L	0
143	138	*Goodenia cirrifica*		subg. Porphyranthus sect. Ebracteolatae	Ebracteolatae II	basal; cauline	raceme	B	L	1
144	106	*Goodenia coronopifolia*		subg. Porphyranthus sect. Ebracteolatae	Ebracteolatae II	basal	raceme	F	L	0
145	130	*Goodenia janamba*		subg. Porphyranthus sect. Ebracteolatae	Ebracteolatae II	basal	raceme; subumbel	F	N	0
146		Goodenia psammophila subsp. psammophila		subg. Porphyranthus sect. Ebracteolatae	Ebracteolatae II	basal; cauline	raceme; subumbel	F	L	0
147		Goodenia psammophila subsp. hiddinsiana		subg. Porphyranthus sect. Ebracteolatae	Ebracteolatae II	basal; cauline	raceme	F	L	0
148	155	*Goodenia arachnoidea*		subg. Porphyranthus sect. Ebracteolatae	Ebracteolatae II	basal; cauline	raceme; subumbel	F	L	0
149		*Goodenia pritzelii*		subg. Porphyranthus sect. Ebracteolatae	NS	basal; cauline	raceme; subumbel	F	L	0
150	137	*Goodenia odonnellii*		subg. Porphyranthus sect. Ebracteolatae	Ebracteolatae II	basal; cauline	raceme; subumbel	F	L	0
151	136	*Goodenia redacta*		subg. Porphyranthus sect. Ebracteolatae	Ebracteolatae II	basal; cauline	raceme; subumbel	F	L	0
152	152	*Goodenia stellata*		subg. Porphyranthus sect. Ebracteolatae	Ebracteolatae II	basal; cauline	raceme	F	L	0
153		*Goodenia crenata*		subg. Porphyranthus sect. Ebracteolatae	Ebracteolatae II	basal; cauline	raceme; subumbel	F	L	0
154	151	*Goodenia hirsuta*		subg. Porphyranthus sect. Ebracteolatae	Ebracteolatae II	basal; cauline	raceme; subumbel	F; B	L	0; 1
155	158	*Goodenia durackiana*		subg. Porphyranthus sect. Ebracteolatae	Ebracteolatae II	basal; cauline	raceme	F	L	0
156	154	Goodenia sepalosa var. sepalosa		subg. Porphyranthus sect. Ebracteolatae	Ebracteolatae II	basal; cauline	raceme	F	L	0
157	154	Goodenia sepalosa var. glandulosa		subg. Porphyranthus sect. Ebracteolatae	NS	basal; cauline	raceme	F	L	0
158	156	*Goodenia brachypoda*		subg. Porphyranthus sect. Ebracteolatae	Ebracteolatae II	cauline	raceme	F	L	0
159	173	*Goodenia heppleana*		subg. Porphyranthus sect. Ebracteolatae	Ebracteolatae II	basal; cauline	raceme	F	L	0
160	174	*Goodenia symonii*		subg. Porphyranthus sect. Ebracteolatae	NS	basal; cauline	raceme	F	L	0
161	176	*Goodenia quadrifida*		subg. Porphyranthus sect. Ebracteolatae	Ebracteolatae II	basal; cauline	raceme	F	L	0
162	162	*Goodenia potamica*		subg. Porphyranthus sect. Ebracteolatae	Ebracteolatae II	basal; cauline	raceme	F	L	0
163	175	*Goodenia purpurea*		subg. Porphyranthus sect. Ebracteolatae	Ebracteolatae II	basal; cauline	raceme	F	L	0
164	172	*Goodenia holtzeana*		subg. Porphyranthus sect. Ebracteolatae	Ebracteolatae II	basal; cauline	raceme; subumbel (rarely)	F	L	0
165	169	Goodenia pilosa subsp. pilosa		subg. Porphyranthus sect. Ebracteolatae	Ebracteolatae II	basal; cauline	raceme	F	L	0
166		Goodenia pilosa subsp. chinensis		subg. Porphyranthus sect. Ebracteolatae	NS	basal; cauline	raceme	F	L	0
167	168	*Goodenia porphyrea*		subg. Porphyranthus sect. Ebracteolatae	Ebracteolatae II	cauline	raceme	F	L	0
168	170	*Goodenia neglecta*		subg. Porphyranthus sect. Ebracteolatae	Ebracteolatae II	basal; cauline	raceme; subumbel	F	L	0
169		*Goodenia inundata*		subg. Porphyranthus sect. Ebracteolatae	Ebracteolatae II	cauline	raceme; subumbel	F	L	0
170	166	*Goodenia armstrongiana*		subg. Porphyranthus sect. Ebracteolatae	Ebracteolatae II	cauline	raceme	F	L	0
171		*Goodenia debilis*		subg. Porphyranthus sect. Ebracteolatae	Ebracteolatae II	basal; cauline	raceme	F	L	0
172		*Goodenia elaiosoma*		subg. Porphyranthus sect. Ebracteolatae	Ebracteolatae II	cauline	raceme	F	L	0
173		*Goodenia heterotricha*		subg. Porphyranthus sect. Ebracteolatae	Ebracteolatae II	cauline	raceme; thyrse	F	L	0
174	164	*Goodenia subauriculata*		subg. Porphyranthus sect. Ebracteolatae	Ebracteolatae II	cauline	raceme	F	L	0
175	160	*Goodenia campestris*		subg. Porphyranthus sect. Ebracteolatae	Ebracteolatae II	basal; cauline	raceme	F	L	0
176	159	*Goodenia byrnesii*		subg. Porphyranthus sect. Ebracteolatae	Ebracteolatae II	basal; cauline	raceme	F	L	0
177	167	*Goodenia argillacea*		subg. Porphyranthus sect. Ebracteolatae	Ebracteolatae II	cauline	raceme	F	L	0
178	161	*Goodenia malvina*		subg. Porphyranthus sect. Ebracteolatae	Ebracteolatae II	cauline	raceme	F	L	0
179	157	*Goodenia leiosperma*		subg. Porphyranthus sect. Ebracteolatae	Ebracteolatae II	basal; cauline	raceme	F	L	0
180	163	*Goodenia hispida*		subg. Porphyranthus sect. Ebracteolatae	Ebracteolatae II	basal; cauline	raceme	F	L	0
181	34	*Goodenia coerulea*		subg. Monochila sect. Coeruleae	Coerulea	basal; cauline	raceme; thyrse	E	N	1
182	37	*Goodenia incana*		subg. Monochila sect. Coeruleae	Coerulea	basal; cauline	raceme	B	L	1
183	39	*Goodenia glareicola*		subg. Monochila sect. Coeruleae	Coerulea	basal	raceme; panicle-like	E	N	1
184	35	*Goodenia trichophylla*		subg. Monochila sect. Coeruleae	Coerulea	basal; cauline	raceme; thyrse	E	N	1
185	36	*Goodenia perryi*		subg. Monochila sect. Coeruleae	Coerulea	basal; cauline	raceme	E	N	1
186	38	*Goodenia pterigosperma*		subg. Monochila sect. Coeruleae	Coerulea	basal; cauline	raceme; thyrse	E	N	1
187	40	*Goodenia eatoniana*		subg. Monochila sect. Coeruleae	Coerulea	basal; cauline	raceme; thyrse	E	L	1
188	42	*Goodenia hassallii*		subg. Monochila sect. Coeruleae	Coerulea	basal; cauline	raceme; thyrse	E	L	1
189		*Goodenia katabudjar*		subg. Monochila sect. Coeruleae	Coerulea	basal; cauline	raceme	E	L	1
190	41	*Goodenia leptoclada*		subg. Monochila sect. Coeruleae	NS	basal; cauline	raceme	E	L	1
191		*Goodenia lancifolia*		subg. Monochila sect. Coeruleae	NS	basal; cauline	raceme	E	L	1
192	59	*Goodenia quadrilocularis*		subg. Monochila sect. Tetraphylax	Tetrathylax	basal; cauline	raceme	E	N	1
193	65	*Goodenia lineata*		subg. Monochila sect. Tetraphylax	Tetrathylax	basal	raceme (panicle?)	E	N	1
194	67a	Goodenia racemosa var. racemosa		subg. Monochila sect. Tetraphylax	Tetrathylax	cauline	raceme; thyrse	G	N	1
195	67b	Goodenia racemosa var. latifolia		subg. Monochila sect. Tetraphylax	NS	cauline	raceme; thyrse	G	N	1
196	64	*Goodenia glomerata*		subg. Monochila sect. Tetraphylax	Tetrathylax	basal; cauline	spike	E	N	1
197	62a	Goodenia dimorpha var. dimorpha		subg. Monochila sect. Tetraphylax	NS	basal; cauline	thyrse-like panicle	E	N	1
198	62b	Goodenia dimorpha var. angustifolia		subg. Monochila sect. Tetraphylax	Tetrathylax	basal; cauline	thyrse-like panicle	E	N	1
199	63	*Goodenia stelligera*		subg. Monochila sect. Tetraphylax	Tetrathylax	basal; cauline	raceme; thyrse	E	N	1
200	66a	Goodenia bellidifolia subsp. bellidifolia		subg. Monochila sect. Tetraphylax	Tetrathylax	basal	raceme	E	N	1
201	66b	Goodenia bellidifolia subsp. argentea		subg. Monochila sect. Tetraphylax	NS	basal; cauline	raceme	E	N	1
202	60	*Goodenia decurrens*		subg. Monochila sect. Tetraphylax	Tetrathylax	basal (indeterminate); cauline	raceme; thyrse	G	N	1
203	61	*Goodenia rostrivalvis*		subg. Monochila sect. Tetraphylax	NS	basal	raceme; thyrse	E	N	1
204		*Pentaptilon careyi*	*Goodenia careyi*	subg. Monochila sect. Verreauxia	Verreauxia	basal; cauline	thyrse	E	N	1
205		*Verreauxia verreauxii*	*Goodenia verreauxii*	subg. Monochila sect. Verreauxia	Verreauxia	basal	raceme; thyrse	E	N	1
206		*Verreauxia reinwardtii*	*Goodenia reinwardtii*	subg. Monochila sect. Verreauxia	Verreauxia	cauline	raceme; spike-like thyrse	G	N	1
207		*Verreauxia dyeri*	*Goodenia etheira*	subg. Monochila sect. Verreauxia	Verreauxia	cauline	raceme; spike-like thyrse	G	N	1
208	4	*Goodenia pinifolia*		subg. Monochila sect. Monochila subsect. Monochila	Monochila	cauline	raceme; thyrse	G	N	1
209	5	*Goodenia elderi*		subg. Monochila sect. Monochila subsect. Monochila	Monochila	cauline	raceme; thyrse	G	N	1
210	26	*Goodenia sericostachya*		subg. Monochila sect. Monochila subsect. Monochila	Monochila	basal; cauline	spike	G	N	1
211		Goodenia scapigera subsp. graniticola		subg. Monochila sect. Monochila subsect. Monochila	Monochila	cauline	raceme; thyrse	G	L	1
212	1	Goodenia scapigera subsp. scapigera		subg. Monochila sect. Monochila subsect. Monochila	Monochila	cauline	thyrse	G	N	1
213	3a	Goodenia watsonii subsp. watsonii		subg. Monochila sect. Monochila subsect. Monochila	NS	basal	thyrse	E	N	1
214	3b	Goodenia watsonii subsp. glandulosa		subg. Monochila sect. Monochila subsect. Monochila	Monochila	basal	thyrse	E	N	1
215	2	*Goodenia decursiva*		subg. Monochila sect. Monochila subsect. Monochila	Monochila	cauline	thyrse	G	N	1
216	7	*Goodenia stenophylla*		subg. Monochila sect. Monochila subsect. Infracta	Monochila	cauline	spike; spike like thyrse	G	N	1
217	8	Goodenia drummondii subsp. drummondii		subg. Monochila sect. Monochila subsect. Infracta	Monochila	cauline	spike; spike-like thyrse	G	N	1
218		Goodenia drummondii subsp. megaphylla		subg. Monochila sect. Monochila subsect. Infracta	NS	cauline	spike; spike-like thyrse	G	N	1
219	9	*Goodenia helmsii*		subg. Monochila sect. Monochila subsect. Infracta	Monochila	cauline	spike; spike-like thyrse	G	N	1
220	6	*Goodenia fasciculata*		subg. Monochila sect. Monochila subsect. Infracta	Monochila	cauline	spike; spike-like thyrse	G	N	1
221		*Goodenia hartiana*		subg. Monochila sect. Scaevolina	Scaevolina	cauline	raceme	B	L	1
222	27	*Goodenia scaevolina*		subg. Monochila sect. Scaevolina	Scaevolina	basal; cauline	raceme; thyrse	B	L	1
223	28	*Goodenia stobbsiana*		subg. Monochila sect. Scaevolina	Scaevolina	basal; cauline	thyrse	B	L	1
224	33	Goodenia azurea subsp. azurea		subg. Monochila sect. Scaevolina	Scaevolina	cauline	raceme; thyrse	B	L	1
225		Goodenia azurea subsp. hesperia		subg. Monochila sect. Scaevolina	Scaevolina	cauline	raceme; thyrse	B	L	1
226	29	*Goodenia suffrutescens*		subg. Monochila sect. Scaevolina	Scaevolina	cauline	thyrse	B	L	1
227		*Goodenia splendida*		subg. Monochila sect. Scaevolina	Scaevolina	cauline	raceme	B	L	1
228	32	*Goodenia ramelii*		subg. Monochila sect. Scaevolina	Scaevolina	basal; cauline	raceme; thyrse	E	N	1
229	31	*Goodenia eremophila*		subg. Monochila sect. Scaevolina	Scaevolina	basal; cauline	thyrse	B	L	1
230	2	*Velleia foliosa*	*Goodenia brendannarum*	subg. Monochila sect. Velleia	Velleia	basal; cauline	axillary dichasia	H	N	1
231	3	*Velleia macrophylla*	*Goodenia macrophylla*	subg. Monochila sect. Velleia	Velleia	basal	axillary dichasia	H	N	1
232	1	*Velleia trinervis*	*Goodenia trinervis*	subg. Monochila sect. Velleia	Velleia	basal	axillary dichasia	H	N, L	1
233	14	*Velleia glabrata*	*Goodenia glabrata*	subg. Monochila sect. Velleia	Velleia	basal	axillary dichasia	H	N, L	1
234	11	*Velleia hispida*	*Goodenia capillosa*	subg. Monochila sect. Velleia	Velleia	basal	axillary dichasia	H	N, L	1
235	13	*Velleia paradoxa*	*Goodenia paradoxa*	subg. Monochila sect. Velleia	Velleia	basal	axillary dichasia	H	N	1
236	10	*Velleia cycnopotamica*	*Goodenia cycnopotamica*	subg. Monochila sect. Velleia	Velleia	basal	axillary dichasia	H	N	1
237	9	*Velleia rosea*	*Goodenia rosea*	subg. Monochila sect. Velleia	Velleia	basal	axillary dichasia	H	N	1
238	12	*Velleia arguta*	*Goodenia arguta*	subg. Monochila sect. Velleia	Velleia	basal	axillary dichasia	H	N	1
239	8	*Velleia discophora*	*Goodenia discophora*	subg. Monochila sect. Velleia	Velleia	basal	axillary dichasia	H	D	1
240	6	*Velleia panduriformis*	*Goodenia panduriformis*	subg. Monochila sect. Velleia	Velleia	basal	axillary dichasia	H	D	1
241	7	*Velleia connata*	*Goodenia connata*	subg. Monochila sect. Velleia	Velleia	basal	axillary dichasia	H	D	1
242	5	*Velleia daviesii*	*Goodenia daviesii*	subg. Monochila sect. Velleia	Velleia	basal	axillary dichasia	H	L	1
243	19	*Velleia macrocalyx*	*Goodenia macrocalyx*	subg. Monochila sect. Velleia	Velleia	basal; cauline	axillary dichasia	H	N	1
244	20	*Velleia perfoliata*	*Goodenia perfoliata*	subg. Monochila sect. Velleia	NS	basal	axillary dichasia	H	D	1
245	21	*Velleia montana*	*Goodenia montana*	subg. Monochila sect. Velleia	Velleia	basal	axillary dichasia	H	N	1
246	15	*Velleia lyrata*	*Goodenia caroliniana*	subg. Monochila sect. Velleia	Velleia	basal	axillary dichasia	H	N	1
247	16	*Velleia parvisepta*	*Goodenia parvisepta*	subg. Monochila sect. Velleia	NS	basal	axillary dichasia	H	N	1
248	18	*Velleia pubescens*	*Goodenia subsolana*	subg. Monochila sect. Velleia	Velleia	basal	axillary dichasia	H	N	1
249	17	*Velleia spathulata*	*Goodenia mystrophylla*	subg. Monochila sect. Velleia	Velleia	basal	axillary dichasia	H	N	1
250*	30	*Goodenia arthrotricha*		subg. Monochila	Clade C (unplaced)	basal; cauline	raceme; thyrse	B	L	1
251*	52	*Goodenia xanthotricha*		subg. Monochila	Clade C (unplaced)	cauline	raceme	B	L	1

Carolin’s (1967a) original classification of inflorescence structure is now revised to eight different morphologies, characterised as Forms A–H (Fig. 3). Carolin’s Type 1 form, seen in the type species *G. ovata* (Fig. 3A), was characterised by the terminal shoot ending with a main inflorescence (MI), subtended by a zone of enrichment (EZ) and then the zone of inhibition or vegetative zone (V) (these labelled HF, BZ and V respectively in Carolin 1967a). The overall structure is a thyrse with leafy bracts, which Carolin observed in other species in subsect. Goodenia such as *G. mueckeana* F.Muell. (see table 1 in Carolin 1967a, note the infrageneric classification in that work follows Krause 1912). The subsequent year’s growth in this inflorescence type is continued by lateral buds in the inhibition zone of the previous year’s growth. In Carolin’s Type 2 form, every partial inflorescence is reduced to 1(–2) flower(s) per raceme, as observed in species of *Coopernookia* and other members of subsect. Goodenia such as *G. laevis* (Fig. 3A inset) and *G. calcarata* (F.Muell.) F.Muell. Type 1 and 2 inflorescence forms intergrade somewhat, as some species such as *G. varia* R.Br. and *G. grandiflora* Sims were recorded by Carolin (1967a) as having both a Type 1 and Type 2 Bauplan. As such, these two inflorescence types have been combined into a new category designated as Form A. This form occurs in all species in the Goodenia I subclade of Clade A, and several species allied to *G. atriplexfolia* in the Goodenia II subclade (Table 2). Other species in this latter clade also exhibit inflorescence Form B previously known as Carolin’s Type 5, which have a basal rosette but with leafy bracteoles, as observed in *G. hederacea* Sm. (Fig. 3B) and species allied to *G. affinis* de Vriese in subsect. Goodenia. A small group in this category from subg. Porphyranthus may have inflorescences that are panicle-like; however, some of the lateral inflorescences from the main stem appear to be monochasial cymes, where the youngest flowers emerge from the axil of a bract below older flowers or may be reduced to a single flower (D. Albrecht pers. comm.). Carolin (1967a) noted that *G. rotundifolia* R.Br. exhibited a Type 5 inflorescence (see table 1, Carolin 1967a), but this species tends to have cauline leaves and leafy racemes rather than a basal rosette, so its inflorescence is correctly categorised as Form A. Carolin also observed that among *G. hederacea* and allied species the main stem may not produce an inflorescence in a given year, presumably due to poor growing conditions, continuing with vegetative growth potentially for several years, as the inflorescences are entirely a product of the enrichment zone.

Two inflorescence groups that were previously not characterised by Carolin are recognised here. A number of small, tufted herbaceous species in the Goodenia II subclade allied to *G. convexa* Carolin show a reduction of the inhibition zone and the vegetative branching zone to form a basal rosette of leaves, but with solitary, bracteolate flowers produced in the axils of the basal leaves. These species are categorised as having a Form C inflorescence (Fig. 3C). While another group of herbs from the Porphyranthus I clade including the diminutive *G. chthonocephala* Carolin, as well as *G. kakadu* and *G. pumilio* previously included in sect. Amphichila along with the recently described *G. cravenii* (Fig. 6C) and *G. oenpelliensis* R.L.Barrett, all have ebracteolate, solitary flowers in leaf axils, which is categorised here as Form D (Fig. 3D). It should be noted that *G. oenpelliensis*, which is currently only known from a single locality in the Northern Territory, has dimorphic inflorescences with both solitary flowers and short, ebracteolate cymes (Barrett and Barrett 2018), the latter type being categorised as Form F (see below and Fig. 3F).

Species characterised as having a Form E inflorescence herein include a diverse group from the Porphyranthus subclades of Clade B and representatives of the Coreulea, Tetrathylax, Verreauxia and Scaevolina subclades of Clade C that have a basal rosette of leaves and inflorescences with non-leafy bracts and bracteoles that form panicles (e.g. *G. paniculata* Sm., Fig. 3E), racemes (e.g. *G. gracilis* R.Br., Fig. 3E inset above), or a thyrse-like inflorescence (e.g. *G. pterygosperma* Krause, Fig. 3E inset below).

Species of *Goodenia* lacking bracteoles from subsects. *Ebracteolatae* and *Borealis* currently placed in the Ebraceolatae subclades of Clade B were variously categorised under Carolin’s inflorescence Types 6–8. Carolin’s Type 6 inflorescence morphology was characterised by leafy, ebracteolate racemes, as observed in various species in subsect. Ebracteolatae such as *G. hispida* R.Br. (Fig. 3F). Previously, *G. pumilio*, from sect. Amphichila, was also recorded as having a Type 6 inflorescence but as stated above, this morphology is now treated as Form D. Carolin’s Type 7 group included species with an inflorescence similar in form to Type 6 but with reduced, non-leafy bracts (e.g. *G. pinnatifida* Schltdl., *G. fascicularis* F.Muell. & Tate (Fig. 3F inset above), and allied species). Carolin (1967a) noted that *G. cycloptera* R.Br. had both Type 6 and Type 7 inflorescences, while *G. filiformis* R.Br., also from subsect. Ebracteolatae, was documented as having both Type 7 and Type 8 inflorescences, the latter form characterised by an inflorescence where the internodes are shortened to form a subumbel and the bracts are leaf-like, as observed in *G. concinna* Benth. (Fig. 2C) and *G. pulchella* Benth. (Fig. 3F inset below). It is now evident that many species in the Ebraceolatae subclades of Clade B may exhibit variations in inflorescence morphology, particularly when growing under varying seasonal conditions, and so Carolin’s inflorescence Types 6–8 are grouped together here under Form F (Table 2).

Carolin’s Type 3 inflorescence (here treated as Form G) was defined as being the same as Type 1 but the bracteoles are reduced rather than leafy. This was observed in members of subg. Monochila, for example *G. scapigera* R.Br. (Figs 2G, 3G) and *G. racemosa* F.Muell., as well as *Verreauxia reinwardtii* (de Vriese) Benth.

Finally, Carolin (1967a) treated species in the genus *Velleia* as having a Bauplan that was a modification of the Type 1 form (recognised here as Form H), where the whole of the terminal “paracladium” (the enrichment zone) is contracted and the inflorescences are elongated with each partial inflorescence expanding into complex branching dichasia or “dichotomous axillary cymes” forming a significant component of the overall plant habit (Carolin 1967c). Carolin (1967a) also noted that the terminal bud apparently continues to grow from year to year.

## Discussion

Taxonomic stability is important, particularly in species-rich groups that are horticulturally popular such as the family Goodeniaceae. Under-sampling in phylogenetic studies can result in premature taxonomic decisions as the addition of further taxa or more informative data may highlight significant incongruencies. This is often most problematic in groups with poor backbone resolution. In light of this, we have been reluctant to make taxonomic changes based on our previous molecular phylogenetic studies, particularly within the morphologically diverse *Goodenias.l.* (Jabaily et al. 2012; Gardner et al. 2016a; Jabaily et al. 2018). Through ongoing studies, we now believe we have addressed key sampling and data issues by including multiple accessions and combining genome skimming and Sanger sequencing from across multiple gene regions and genomic compartments. A number of potentially new phrase-named species (designated by ‘sp.’ and a relevant phrase name e.g. *Goodenia* sp. Mount Bomford (M.D. Barrett 423)) that are currently recognised on Australian plant name databases (Council of Heads of Australasian Herbaria, 2006-; Western Australian Herbarium, 1998-), and variants that show an affinity to but may be distinct from current species (‘aff’), were also included in this study. Australia has a high level of species discovery and description (Wege et al. 2015), and yet many taxa that have been provisionally recognised as new are yet to be formally named and described. Western Australia is a centre for diversity for many groups including *Goodenia* with more than 70% of known species of this genus found there. Given that approximately 44% of the state’s undescribed taxa are listed as being poorly known and of conservation concern (Smith and Jones 2018), with many facing continued significant threat due to land clearing and habitat fragmentation, fire, weed invasion, disease and climate change, it is essential that the description of new taxa is expedited. By including variants and phrase-named taxa in molecular phylogenies, their closest allied taxa can be confirmed, thus focusing taxonomic study to the most relevant species group to facilitate their taxonomic resolution.

The results of this molecular study reconfirm our earlier findings where *Goodenias.l.* is paraphyletic with respect to *Pentaptilon*, *Selliera*, *Velleia*, and *Verreauxia* (Jabaily et al. 2012; Gardner et al. 2016a; Jabaily et al. 2018). Unfortunately, there are no obvious synapomorphies that define this broad group and yet the various included genera are relatively morphologically well circumscribed. This led to earlier suggestions that *Goodenia* could potentially be more narrowly defined to represent only the species within Clade A (including *Selliera* and *Scaevola collaris*), as the newly conserved type *G. ovata* (Shepherd et al. 2017) falls within this clade. In this case, *Pentaptilon*, *Velleia* and *Verreauxia* would be retained along with several newly reinstated or circumscribed segregate genera. However, this outcome would be significantly more taxonomically disruptive as around 160 name changes would be required, mostly in the species-rich Clade B where the earliest available name is *Calogyne*. Moreover, phylogenetic under-resolution and conflict remains within floristically diverse Clade C, likely due to recent radiation, possible hybridisation and incomplete lineage sorting. This, in conjunction with our re-assessment of key morphological characters including inflorescence form and ovary structure, and expanded molecular phylogenetic data, has led us to the pragmatic taxonomic decision to synonymise *Pentaptilon*, *Selliera*, *Velleia*, and *Verreauxia* into an expanded *Goodenia*.

### Synonymisation of *Diaspasis*

*Scaevola* is not discussed in detail in this study other than to provide the new combination for the monotypic Western Australian genus *Diaspasis* (Jabaily et al. 2012). *Diaspasis* was first recognised as distinct by Robert Brown (1810) due to its nearly actinomorphic flowers (see Fig. 1E in Jabaily et al. 2012) and connate anthers. *Scaevola* by contrast, typically has fan-like flowers with free anthers (Carolin et al. 1992); however, shared characters between these genera include a dry indehiscent fruit with a hard endocarp and a spathulate embryo (cf. terete in the LAD clade *sensu*Jabaily et al. 2012) and similar trichomes (Carolin 1971). Moreover, Carolin (1959) noted that *D. filifolia* had a similar anatomical floral and stylar structure to *Scaevola albida* (Sm.) Druce and *S. hookeri* (de Vriese) F.Muell. ex Hook.f. Recent floral morphometric analyses of the Core Goodeniaceae have shown that floral symmetry is quite labile across the family with various species of *Scaevola* tending towards a pseudo-radial symmetry (e.g. *S. phlebopetala* F.Muell.: see Gardner et al. 2016b) somewhat like the form of *D. filifolia*. Molecular data also supports *Diaspasis* as congeneric with *Scaevola* (Howarth et al. 2003; Jabaily et al. 2012; Gardner et al. 2016a) and so a new combination for this species is provided.

### No synapomorphic characters define *Goodenias.l.*

Key characters previously used to distinguish *Goodenia* (Carolin 1992e) include bilabiate flowers, an inferior ovary that has 2 incomplete locules with > 2 ovules either present in two rows or scattered over the surface of the placenta, and fruits being dry, bi-valved, dehiscent capsules with flat seeds that have a rim or wing (Fig. 2F). Carolin (1992e) did note there were exceptions, such as *G. neogoodenia* and some representatives of the fan-flowered subg. Monochila, which have 1-seeded, indehiscent nuts, as does the newly included *Scaevola collaris* (Fig. 2B). Moreover, molecular sequence data show that species of *Selliera* that have indehiscent dry or fleshy fruits are also embedded within *Goodenia*. Clearly these various indehiscent fruits are superficially similar, but evidently non-homologous, to the indehiscent fruits of *Scaevola*; a diagnostic character for that genus. Further, the fan-flower form typical for *Scaevola* has also evolved independently across every major clade in *Goodenia* (Gardner et al. 2016b). It was evident to Carolin (1966) that while *Scaevola* and *Goodenia* were allied, these genera had distinct evolutionary histories and that the “similarities in the ovary structure are the result of convergence rather than common origin” as the locules in the ovary of *Goodenia* are derived from two carpels rather than one as evident in *Scaevola* (Carolin 1959). While there are no easily discernible synapomorphies available for *Goodenias.l.*, this is a well-supported clade, so a combination of characters is required to recircumscribe this genus. Thus, a revised classification based on our understanding of phylogenetic relationships within the newly expanded *Goodenia* is outlined, recognising monophyletic groups at infrageneric levels (Table 1) including three newly circumscribed subgenera *Goodenia*, *Porphyranthus* and *Monochila* representing Clades A, B and C respectively.

### New infrageneric taxa within subgenus Goodenia (Goodenia Clade A)

Subgenus Goodenia as recognised herein reflects Carolin’s (1992e) subsect. Goodenia in most respects, with two major clades (Goodenia I and Goodenia II) now formally recognised as sections (Table 1). Goodenia I includes the newly conserved type species *G. ovata* (Shepherd et al. 2017) and so represents sect. Goodenia, while Goodenia II is a recircumscription of Krause’s (1912) ser. Rosulatae K.Krause, recognised herein as sect. Rosulatae (K.Krause) K.A.Sheph.

In the combined molecular analysis, Goodenia I includes *G. phillipsiae*; a species previously placed in sect. Ebracteolatae despite the presence of bracteoles, although Carolin (1992e) did acknowledge that this was a species of “uncertain affinity” as apparent related species were ebracteolate. The typical subclade with *G. ovata* and allied species includes prostrate or decumbent subshrubs, many of which have long stoloniferous branches that may root at the nodes (Fig. 2I). These plants are usually glabrous or viscid, with bright yellow bilabiate flowers where the dorsal lobes are erect and sometimes overlapping (Fig. 5A, E–G). One exception is *G. viscida* (Fig. 5C), an erect fan-flowered subshrub from south-west Western Australia. Carolin et al. (1992) was also uncertain of the systematic placement of this species but included it in subg. Monochila due its yellow fan-shaped flowers, despite the fact *G. viscida* “has a seed-surface pattern unlike any other species [within the subgenus] and is therefore likely to be misplaced” (Carolin 1980).

The second subclade in Goodenia I includes *Selliera*, a small genus of prostrate, woody perennials with white or pale pink fan-flowers that have fleshy fruits that become woody and corky with age, which are found near coastal, winter-wet or saline flats (Fig. 5B) in Australia, New Zealand and Chile. Also in this clade is *Scaevola collaris* (Fig. 2C), a widespread fan-flowered species often found around the margins of salt lakes across arid Australia with a uniquely beaked fruit with a sponge-like woody endocarp. This unusual species is placed sister to *G. laevis*, which is confined to the southern regions of Western Australia and has capsular fruits as seen in the rest of sect. Goodenia. However, in a surprising twist, recent analysis of new ITS sequence data has shown that the enigmatic species *Velleia exigua* (F.Muell.) Carolin is in fact more closely allied to *G. laevis* (R. Jabaily, unpublished data). Initial attempts to molecular sequence *V. exigua* had failed, and its systematic position was equivocal. This species was previously included in *Goodenia* (as *G. exigua* F.Muell.) and while Carolin (1992g) transferred it to Velleia sect. Euthales, he noted that it was unlike other species of the genus due to the presence of solitary and almost sessile flowers, and sepals that were adnate to the ovary in the lower half (in contrast to all other species where the sepals are adnate to the base of the ovary). The indusium of *V. exigua* was also considered to be unique within *Velleia*, but on close inspection it is remarkably similar to that seen in *Goodenia viscida* and indeed *Scaevola collaris*, which was described by Carolin (1992g) as being obloid and longer than it is wide. Moreover, the indusium in these species is notched at the apex and has no obvious fringing hairs on the lips (Fig. 5C), although tiny hairs are present on the indusium of *S. collaris*. Based on morphological and molecular evidence, it is now clear that *V. exigua* should be included in sect. Goodenia.

The final subclade recovered in the Goodenia I clade includes several erect, glabrous subshrubs allied to *G. kingiana* Carolin, that have articulated pedicels and large bilabiate flowers where the dorsal petals are spreading to expose a long style supporting a broad indusium. The South Australian species *G. saccata* Carolin was not sequenced, but it is morphologically allied to *G. grandiflora* Sims and so is included in the typical section.

The Goodenia II subclade, treated here as sect. Rosulatae (K.Krause) K.A.Sheph., includes erect or decumbent shrubs and herbs with rosulate and/or cauline leaves that are often covered in a dense tomentum of soft, multicellular hairs. Some species such as *G. rotundifolia* are variable and may be glabrous or have a mix of simple, glandular and multicellular hairs. There are two well supported subclades in this section that are not taxonomically recognised. In the first, *G. fordiana* Carolin is placed sister to a subclade of small tufted herbs from southern Australia allied to *G. convexa*. These species have cottony, multicellular hairs and solitary flowers that are sometimes supported by a distinctly geniculate pedicel that is sharply bent at the point of bracteole attachment. Sister to this, is a less well-resolved group allied to the widespread *G. glabra* R.Br. that includes decumbent herbs or subshrubs from northern and central Australia, which may also have a geniculate pedicel. The second monophyletic group in the Goodenia II subclade includes decumbent plants that occur in more arid inland regions of Australia with a centre of diversity in Queensland. Two potentially new species informally known as *G.* sp. Carnarvon Range (D.J. Edinger Nats 30) and *G.* sp. Mt Castletower (M.D. Crisp 2753) (Council of Heads of Australasian Herbaria 2006-), also fall within this clade. Finally, *G. arenicola* Carolin, a species currently only known from the type locality on Stradbroke Island in Queensland, as well as *G. robusta* (Benth.) K.Krause and *G. rupestris* Carolin, were not successfully sequenced, but are included in sect. Rosulatae as they share key diagnostic characters such as low prostrate habit and a tomentum of multicellular hairs. According to Carolin (1992e), *G. stephensonii* F.Muell. is allied to *G. heterophylla* Sm. and this species is placed in sect. Rosulatae for now; however, *G. stephensonii* is glandular hairy and somewhat viscid, features that are common to species included in sect. Goodenia.

*Goodenia wilunensis*, *G. xanthotricha*, and a group of species allied to *G. quadrilocularis* were all previously included in Carolin’s (1992e) subsect. Goodenia, but based on molecular and morphological data these species are now excluded from our recircumscribed Goodenia and will be discussed in later sections.

### Recognition of *Porphyranthus* as a new subgenus (Goodenia Clade B)

Section Porphyranthus is elevated to subgeneric rank herein as subgen. Porphyranthus (G.Don) K.A.Sheph., which encompasses the variation evident across the monophyletic Clade B. Two sections corresponding to the two major subclades within this new subgenus are also recognised. G.Don’s (1834) original sect. Porphyranthus, represented by Porphyranthus I and II in the molecular analyses, is expanded to include sect. Amphichila. While Krause’s (1912) subsect. Ebracteolatae, encompassing the Ebracteolatae I and II clades, is recircumscribed to include both ser. Borealis and Calogyne of sect. Borealis and elevated to a section, recognised here as sect. Ebracteolatae (K.Krause) K.A.Sheph. (Table 1).

The reinstatement of *G. rosulata* Domin and recognition of a further three new species (Albrecht 2002; Holland and Boyle 2002; Pellow and Porter 2005; Sage and Shepherd 2007) in sect. Porphyranthus in recent decades has resulted in the expansion of this section from Carolin’s (1992e) concept. The majority of species in this group are herbaceous annuals or perennials that grow in sandy soils and winter wet situations and creek beds in eastern and northern Australia, with *G. purpurascens* R.Br. also found in New Guinea (Carolin 1992e). These species generally have basal leaves and spreading, bracteolate inflorescences that may comprise a large part of the plant, and glossy, round seeds that are small (less 1 mm wide) with a very narrow mucilaginous wing c. 0.1 mm wide. *Goodenia kakadu*, *G. pumilio* and the recently described *G. cravenii* (Fig. 6C) and *G. oenpelliensis* (Barrett and Barrett 2014, 2018), are currently included in sect. Amphichila. These small, sometimes mat-like herbs have tiny reddish-purple fan-like flowers and small convex seeds with a minute wing. All four species are here transferred to sect. Porphyranthus along with *G. chthonocephala*, a species previously included in ser. Borealis that has an unusual cushion-like habit and tiny flowers held in a dense head at ground level. The Western Australian *G. neogoodenia*, originally recognised as the monotypic *Neogoodenia minutiflora* due to its tiny wingless corolla, and enlarged, indehiscent, 1-celled fruit (Gardner and George 1963), was transferred by Carolin (1990) to *Goodenia* (in subsect. Ebracteolatae). On molecular evidence it is clear *G. neogoodenia* is allied to a group of species in sect. Porphyranthus that are often associated with the margins of inland salt lakes including *G. halophila* and *G. gypsicola* Symon (Fig. 2H).

A potentially new Western Australia species, *G.* sp. Mount Bomford (M.D. Barrett 423), placed sister to the purple-flowered *G. gloeophylla* Carolin. Similarly, *G.* sp. Dampier Peninsula (B.J. Carter 675) is shown to be allied to a group that may exhibit an aquatic phase when growing under flooded conditions producing distinctive floating leaves with long petioles as observed in *G. lamprosperma* F.Muell. (Carolin 1992e) and *G. berringbinensis* Carolin (Gibson 2014). *Goodenia nocoleche* Pellow & J.L.Porter, *G. paludicola* Carolin, *G. paniculata* and *G. corralina* L.W.Sage & K.A.Sheph. were not sequenced; however, they are here included in sect. Porphyranthus due to the shared presence of a floriferous inflorescence, leafy bracts, bracteoles, and small seeds, with *G. nocoleche* also recorded as producing aquatic leaves under flood conditions (Pellow and Porter 2005).

Over the last two decades, 10 new species have been recognised in Carolin’s (1992e) subsect. Ebracteolatae (Sage 2000; Sage 2001; Sage and Dixon 2005; Sage and Shepherd 2007; Barrett and Barrett 2014; Barrett and Barrett 2018). This is currently the largest infrageneric group in *Goodenia* and is characterised by a lack of bracteoles, generally yellow flowers, and distinctively winged seeds. Many species are annuals or herbaceous perennials found in the more arid regions of the Australian continent. Indeed, it is evident that the Eremaean interior has been an important source and sink for diversification within this group (Jabaily et al. 2014). In these arid regions, many species are confined to damp areas around the margins of creeks and lakes that germinate or regenerate from rootstock after significant cyclonic rainfall (Sage and Pigott 2003), thus ‘avoiding’ harsher seasonal conditions during the long dry season. Of interest is the Western Australian bracteolate *G. wilunensis* that placed sister to the Ebracteolatae clade. While this section is characterised as generally being ebracteolate, there are a few other species within this clade that do retain this character, such as *G. nigrescens* Carolin and *G. cirrifica* F.Muell.

As stated, sect. Ebracteolatae as recognised here, is expanded to include the former ser. Borealis and Calogyne of subsect. Borealis, a group characterised by leafy inflorescences, a lack of bracteoles and seeds with a prominent rim rather than an obvious wing. Since the *Flora of Australia* treatment (Carolin 1992e), three new species, *G. inundata* L.W.Sage & J.P.Piggot, *G. debilis* A.E.Holland & T.P.Boyle and *G. elaiosoma* Cowie, have been included in ser. Borealis (Sage 2001; Holland and Boyle 2002; Cowie 2005), a group segregated on the presence of a simple style and broad sepals to 2.5 mm wide, in contrast to the divided style and narrow sepals to 0.4 mm wide that distinguished ser. Calogyne. Species in this former subsection are found in northern Australia, with the exception of *G. armstrongiana* de Vriese (ser. Borealis), which also occurs in New Guinea, while the widespread and variable *G. pilosa* (R.Br.) Carolin (ser. Calogyne) is found in damp areas in Northern Australia, New Guinea, Indonesia, Malaysia, Philippines and China.

Further species currently included in Carolin’s subsect. Ebracteolatae, such as *G. concinna* (Fig. 2C), *G. symonii* (Carolin) Carolin, *G. fascicularis*, the recently recognised *G. effusa* A.E.Holland (Holland 2015), and reinstated *G. pritzelii* (Barrett and Barrett 2018), were not successfully sequenced. Material was also unavailable for the very poorly understood *G. salmoniana* (F.Muell.) Carolin and *G. pallida* Carolin, which are only known from type collections from the Gascoyne and Pilbara regions in Western Australia. All of these species are retained within this newly circumscribed section due to the presence of diagnostic characters and the confirmed phylogenetic position of morphologically allied species; however, the position of *G. salmoniana* is equivocal as this species was originally placed in *Velleia* by Mueller (as *V. salmoniana* F.Muell.), most likely because its sepals are fused to the lower half of the ovary and the indusium lips are glabrous, unlike other species in this group.

### Expansion of subg. Monochila (Goodenia Clade C)

Clade C is the most morphologically diverse clade in *Goodenias.l.* and, while relationships between some of the subclades are unclear, each is generally supported as monophyletic. As such, subg. Monochila is expanded herein to include all members of Clade C, with most subclades formally named at the sectional level.

Typical sect. Monochila is easily recognised as all members (except the newly included *G. sericostachya* C.A.Gardner) have white, fan-shaped flowers (Fig. 2D) and a narrow indusium that is supported by a style covered in stiff, short, spreading hairs (Carolin 1992e). *G. sericostachya*, a narrow range endemic from Western Australia, was previously included in subsect. Scaevolina due to its dense indumentum of silver white hairs and pink fan-like flowers with a yellow throat (Fig. 8G). However, on close inspection it is evident that this species has the distinctive short hairs on the style and the narrow indusium that are diagnostic for sect. Monochila, confirming its affinity to other species in this clade. Two subsections are further recognised in sect. Monochila. The typical subsection includes all species with short white hairs on the style and capsular fruits, while the remaining species with short purple hairs on the style and a nut-like fruit are now included in a new subsection named herein as subsect. Infracta K.A.Sheph.

The majority of Carolin’s (1992e) species included in his subsect. Goodenia fall within Goodenia Clade A; however, a small clade of yellow-flowered species were found to be nested within Goodenia Clade C (Jabaily et al. 2012; Gardner et al. 2016a). Don’s (1834) sect. Tetrathylax G.Don, which previously only comprised the Western Australian narrow range endemic *G. quadrilocularis* (Figs 2A, 8D), is here resurrected and expanded to include this group, represented by several diploid and polyploid taxa (Peacock 1963) from eastern Australia. sect. Tetrathylax is superficially similar to species in Clade A, due to the presence of yellow, bilabiate flowers, but this section is characterised by distinctive inflorescences comprising long, leafless, interrupted spikes, racemes or panicles where the upper pedicels have short, linear bracteoles close to the flowers with the lower ones being more distant. Carolin (1980) also noted that this group is characterised by unique seeds, that have a “sinuous-areolate” seed coat with the shape of the radial wall thickening as “type 4”. *Goodenia rostrivalvis* Domin was not sequenced, but is included in sect. Tetrathylax due to its morphological similarity to allied species such as *G. decurrens* R.Br.

In Carolin’s (1992e)*Flora of Australia* treatment, sect. Coeruleae comprises subsections *Scaevolina* and *Coeruleae*, and is represented by blue-flowered species of *Goodenia* where the septum of the ovary is at least 2/3 as long as the locule. In our molecular analyses, these subsections are each supported as monophyletic, but they never group together (Jabaily et al. 2018). Accordingly, they are treated as separate sections in our new classification. sect. Coeruleae is re-circumscribed here to only include the members of the former subsect. Coeruleae, representing the blue-flowered perennial herbs and low subshrubs from southwest Western Australia with seeds that have a dry, membranous wing greater than 0.1 mm wide. This section now also includes *G. katabudjar* Cranfield & L.W.Sage and *G. lancifolia* L.W.Sage & Cranfield (Cranfield and Sage 1997; Sage 2000). The latter species was not placed in the phylogeny, along with *G. leptoclada* Benth., due to poor sequence results but both species share the diagnostic characters of this section.

Sect. Scaevolina represents the predominantly northern Australian blue-flowered perennials that have seeds with a narrow, mucilaginous wing c. 1 mm wide and has been expanded to include G. azurea subsp. hesperia L.W.Sage & Albr., *G. hartiana* L.W.Sage and *G. splendida* A.E.Holland & T.P.Boyle since the publication of Carolin’s *Flora* treatment (Holland and Boyle 2002; Sage and Albrecht 2006).

Two species previously included in subsect. Scaevolina have a more southern distribution than typical. The first is the unusual fan-flowered *G. sericostachya* that is now included in sect. Monochila. The second is the rare species *G. arthrotricha* (Smith and Jones 2018), whose broader phylogenetic relationships remain unclear. In all molecular analyses, this species forms a well-supported clade with the short-range endemic *G. xanthotricha* (Fig. 8A), but their relationship to other subclades remains equivocal. While *G. xanthotricha* was previously included in sect. Goodenia, Carolin (1992e) acknowledged that it was “a species difficult to place” noting that even though it has blue flowers, the seed coat ornamentation is “aculeate” rather than “colliculate-punctate” as seen in other members of subsect. Scaevolina. Furthermore, though members of the current sect. Scaevolina generally have an indusium that is longer than wide, the indusium in *G. arthrotricha* is wider than long, similar to that observed in many of the species of sect. Coeruleae. *Goodenia arthrotricha* and *G. xanthotricha* form a very weakly supported association with the southern *Coeruleae* clade in the nrITS analysis, but neither species has a seed with a dry wing > 0.1 mm wide (see the seed rim in Fig. 2F) that characterise this group. Both *G. arthrotricha* and *G. xanthotricha* are naturally rare, but somewhat surprisingly, their distribution overlaps as both species are found in a nature reserve situated on the Dandaragan plateau in Western Australia, although never observed as co-occurring (Western Australian Herbarium 1998-). One could hypothesise that their relatively close situation and morphological features that show some congruence to both sect. Coeruleae and *Scaevolina*, could suggest that one or both may be of possible hybrid origin. As these species are difficult to place systematically and no obvious synapomorphy supports them as distinct from other blue-flowered species, they currently remain unplaced within subg. Monochila.

*Verreauxia* is a small genus of three species from southwest Western Australia characterised by simple, unbranched and branched multicellular hairs, glandular hairs with multicellular heads (Carolin 1971), and a unilocular ovary that becomes indehiscent and nut-like in fruit. The closely allied monotypic *Pentaptilon* (Carolin 1992h), which occurs around the northernmost border between the South-West and Eremaean Botanical Provinces in Western Australia, has similarly unusual branched hairs; however, it was recognised as distinct due to its uniquely winged ovary and fruit, and morphologically distinct seeds. These genera group together in a monophyletic subclade within Clade C and so are recognised here as sect. Verreauxia in subg. Monochila.

The final monophyletic group that consistently placed sister to the rest of the morphologically diverse Clade C (Jabaily et al. 2018) is the genus *Velleia* characterised by inflorescences that are axillary dichasia, which form most of the plant habit (although sometimes flowers may be solitary), and a predominantly superior ovary (Fig. 2G). Carolin (1980) also noted that while the seeds in some *Velleia* showed similarity to various species of *Goodenia*, a number displayed a ‘characteristic wrinkling’. Carolin’s (1992g) infrageneric classification of *Velleia* recognised three sections, based on the presence of three sepals (sect. Velleia) or five, which may be either connate into a tube (sect. Euthales) or free (sect. Menoceras); however, in our analyses sect. Menoceras was shown to be paraphyletic and there was only moderate support for some of these former sections in the chloroplast analyses. As such, we propose here to reduce *Velleia* to a section of subg. Monochila and to not formally recognise any further groups within it. *Velleia parvisepta* Carolin and *V. perfoliata* R.Br., a narrow range endemic from New South Wales, while not sequenced are retained within this section as both species have the typical Form H inflorescence and sepals fused to near the base of the ovary, which are characters typical for sect. Velleia. It should be noted that while *V. perfoliata* is placed after *V. macrocalyx* de Vriese in the proposed updated linear sequence for the genus (Table 2), Carolin (1992g) noted that this poorly known species had connate bracteoles that form a disk-like funnel unlike other species in his sect. Velleia. Three species in the former sect. Menoceras (*V. discophora* F.Muell., *V. panduriformis* A.Cunn. ex Benth. and *V. connata* F.Muell.) have similarly modified bracteoles to *V. perfoliata* (Table 2) and future sequencing of this species may confirm it is more closely allied to this group of taxa.

### Conclusion

While this could be considered the final chapter of our detailed study of *Goodenias.l.*, resulting in a new understanding and an updated classification of this captivating group, it is not likely to be the final word. *Goodenias.l.* represents an outstanding clade for further studies, particularly of inflorescence and floral form, seed traits, and the potential impacts of adaptations on rates of diversification. These well sampled and resolved phylogenies also allow for the inclusion of Goodeniaceae in meta-studies of diversification patterns across Australia and in other biodiversity hotspots like the Southwest Australian Floristic Region (SWAFR) (Jabaily et al. 2014). Furthermore, this framework will support more in-depth studies at the species level to hopefully expedite the recognition of many new but as yet unnamed taxa.

### Taxonomic treatment

In this present treatment, revised descriptions of infrageneric groups are provided with a synopsis of the species currently recognised therein, including updated nomenclatural changes. An updated key to genera in the family, including *Brunonia* (previously placed in the monotypic Brunoniaceae), and incorporating *Selliera*, *Pentaptilon*, *Velleia* and *Verreauxia* into *Goodenia* is also provided. A key is also provided to infrageneric groups as recognised in this paper.

### Key to Genera (modified from Carolin et al. 1992, previously recognised genera in parentheses)

**Table:** 

1a	Anthers connate	**2**
1b	Anthers free	**6**
2a	Ovules and seeds more than 2 per locule; fruit a dehiscent capsule or fragmenting into articles, rarely an indehiscent beaked fruit	**3**
2b	Ovules and seeds 1 or 2 per locule; fruit an indehiscent nut, not beaked	**4**
3a	Leaves all cauline; indusium 2-lipped with stigmatic tissue on the outer surface	*** Lechenaultia ***
3b	Leaves cauline and basal; indusium cup-like with stigmatic tissue inside	*** Anthotium ***
4a	Inflorescence a solitary capitulum on a naked scape; corolla lobes connate towards the base	*** Brunonia ***
4b	Inflorescence a loose, terminal raceme; corolla lobes free at the base	**5**
5a	Corolla auriculate; hairs branched, rarely absent	*** Dampiera ***
5b	Corolla without auricles; hairs simple	***Scaevola filifolia* (*Diaspasis*)**
6a	Ovules (and usually seeds) more than 2 per locule	**7**
6b	Ovules and seeds 1 or 2 per locule	**8**
7a	Corolla with long, stiff bristles inside; seeds glossy, strophiolate, without a wing	*** Coopernookia ***
7b	Corolla without bristles inside (may have hairs or enations); seeds dull, estrophiolate, with or without a wing	***Goodenia* (*Velleia* ; *Selliera*)**
8a	Plants glabrous or with simple hairs; flowers fan-like (rarely pseudoradial); ovary glabrous or with simple hairs, without wings	**9**
8b	Plants with branched and simple hairs; flowers bilabiate; ovary with dense multicellular hairs in 3 lines, sometimes winged	***Goodenia* (*Verreauxia; Pentaptilon*)**
9a	Style with short, stiff hairs at 90°	**Goodenia (subg. Monochila)**
9b	Style without short, stiff hairs at 90°	**10**
10a	Indusium broad (length equal to or shorter than width); fruit without a distinctive beak	*** Scaevola ***
10b	Indusium narrow (length longer than width); fruit with a distinctive beak up to 6 mm long	***Goodenia* (*Scaevola collaris*)**

### Key to infrageneric groups within Goodenia (modified from Carolin et al. 1992, previously recognised taxa in brackets)

**Table:** 

1a	Sepals variously adnate to the ovary (ovary appears inferior)	**2**
1b	Sepals adnate to the ovary basally (ovary appears superior)	**15**
2a	Bracteoles present	**3**
2b	Bracteoles usually absent (if rarely present then < 3 mm long and deltoid)	**13**
3a	Flowers fan-like	**4**
3b	Flowers bilabiate	**7**
4a	Ovules and seeds > 2	**subg. Goodenia sect. Goodenia (Selliera)**
4b	Ovules and seeds 1 or 2	**5**
5a	Corolla lobes fused for more than 2/3 of their length, cream with a purplish-brown blush towards the base; fruit with a beak 1–6 mm long	**subg. Goodenia sect. Goodenia (*Scaevola collaris*)**
5b	Corolla lobes free for more than 2/3 their length, white, cream or yellow with purplish spots or pink with a yellow throat; fruit without a beak	**6**
6a	Style with short white hairs at 90°; fruit a cylindrical to ovoid capsule	**subg. Monochila sect. Monochila subsect. Monochila**
6b	Style with short purple hairs at 90°; fruit a globular to subglobular nut	**subg. Monochila sect. Monochila subsect. Infracta**
7a	Ovary with dense multicellular hairs in 3 lines; fruit sometimes winged	**subg. Monochila sect. Verreauxia (*Verreauxia* ; *Pentaptilon*)**
7b	Ovary glabrous or with simple hairs; fruit not winged	**8**
8a	Ovules numerous, scattered over the surface of the placenta; seeds < 1 mm diameter	**subg. Porphyranthus sect. Porphyranthus**
8b	Ovules and seeds in two rows in each locule; seeds >1 mm diameter	**9**
9a	Corolla blue, often yellowish in throat; seeds colliculate or reticulate	**10**
9b	Corolla usually yellow or cream, rarely blue; seeds tuberculate or smooth and glossy	**11**
10a	Subshrubs or herbs, usually densely glandular-hairy; seeds with narrow mucilaginous wing c. 0.1 mm wide	**subg. Monochila sect. Scaevolina**
10b	Herbs with basal stock, glabrous or with simple or glandular hairs; seeds with membranous wing > 0.1 mm wide	**subg. Monochila sect. Coeruleae**
11a	Plants with or without a basal rosette of leaves; inflorescence a thyrse, raceme or spike with leafy bracts and bracteoles or with flowers solitary in leaf axils; seed coat various, not sinuous-areolate	** Goodenia **
11b	Plants usually with a basal rosette of leaves; inflorescence a long, leafless, interrupted spike, raceme or panicle; seed coat sinuous-areolate	**subg. Monochila sect. Tetrathylax**
12a	Erect or stoloniferous herbs or subshrubs with cauline leaves, glabrous or viscid with glandular and simple hairs	**subg. Goodenia sect. Goodenia and *G. nigrescens* , *G. cirrifica***
12b	Decumbent or prostrate herbs with tufted or rosulate leaves, with simple, multicellular and/or glandular hairs, these often forming a dense, soft indumentum	subg. Goodenia sect. Rosulatae and *G. wilunensis*, G hirsuta*
13a	Flowers fan-like, without auricles	**subg. Porphyranthus sect. Porphyranthus (sect. Amphichila)**
13b	Flowers bilabiate, auriclate	**14**
14a	Flowers minute c. 1 mm long with a solitary ovule; fruit indehiscent	**subg. Porphyranthus sect. Porphyranthus (*G. neogoodenia*)**
14b	Flowers > 1 mm long with ≤ 30 ovules; fruit usually a dehiscent capsule	**subg. Porphyranthus sect. Ebracteolatae (subsect. Ebracteolatae; subsect. Borealis)**
15a	Bracteoles present; inflorescences in dichasia or flowers solitary in axils of basal leaves	**subg. Monochila sect. Velleia (Velleia)**
15b	Bracteoles absent; inflorescence a raceme or subumbel	**subg. Porphyranthus sect. Ebracteolatae (*G. macroplectra*)**

#### Scaevola

Taxon classificationPlantaeAsteralesGoodeniaceae

L., Mant. Pl. 2: 145. 1771 nom. cons.

467CB9AA-C0F9-5F99-B670-B1AA4261D08B

= Diaspasis R.Br., Prodr. 586. 1810, syn. nov. – Type: D. filifolia R.Br. ≡ Scaevola filifolia (R.Br.) K.A.Sheph.
= Roemeria Dennst., Schlüssel Hortus malab. 24. 1818, *nom. illeg.* [non Roemeria Medik., Ann. Bot. (Usteri) 1(3): 15. 1792] – Type: R. lobelia Dennst. ≡ Scaevola taccada (Gaertn.) Roxb.
sect. Crossotoma = Scaevola sect. Crossotoma G.Don, Gen. hist. 3: 730. 1834 ≡ Crossotoma (G.Don) Spach., Hist. nat. vég. 9: 583. 1838 – Type: Scaevola spinescens R.Br.
= Scaevola sect. Pogonanthera G.Don, Gen. hist. 3: 729. 1834 ≡ Pogonanthera (G.Don) Spach, Hist. nat. vég. 9: 583. 1838, *nom. illeg.* (non Pogonanthera Blume, Flora 14: 520. 1831) – Type: Scaevola striata R.Br.
= Scaevola sect. Xerocarpa G.Don, Gen. hist. 3: 729. 1834 ≡ Xerocarpa (G.Don) Spach, Hist. nat. vég. 9: 583. 1838 – Type: Scaevola crassifolia Labill.
= Pogonetes Lindl., Intr. nat. syst. bot., ed. 2, 443. 1836, *nom. inval., nom. nud.*
= Camphusia de Vriese, Ned. Kruidk. Arch. 2: 148. 1851 – Type: C. glabra (Hook. & Arn.) de Vriese ≡ Scaevola glabra Hook. & Arn.
= Merkusia de Vriese, Ned. Kruidk. Arch. 2: 150. 1851 – Type: M. crassifolia (Labill.) de Vriese ≡ Scaevola crassifolia Labill.
= Temminckia de Vriese, Ned. Kruidk. Arch. 2: 141. 1851 – Type: T. mollis de Vriese ≡ Scaevola mollis Hook. & Arn.
= Molkenboeria de Vriese, Natuurk. Verh. Holl. Maatsch. Wetensch. Haarlem ser. 2, 10: 38. 1854 – Type: M. pilosa (Benth.) de Vriese ≡ Scaevola pilosa Benth.
= Nigromnia Carolin, Nuytsia 1: 292 (1974) – Type: N. globosa Carolin ≡ Scaevola globosa (Carolin) Carolin.

##### Type

(designated by W.R. Greuter et al. (eds), Reg. Veg. 118: 276. 1988). *Scaevola lobelia* L., *nom. illeg., typ. cons*. ≡ *Scaevola plumieri* (L.) Vahl.

#### Goodenia

Taxon classificationPlantaeAsteralesGoodeniaceae

Sm., Spec. bot. New Holland 15. 1793, nom. cons. (fide Shepherd et al. 2017; Applequist 2019)

E2C885BB-0A98-5F70-AD0C-B9C8D66EB494

##### Type.

*G. ovata* Sm., *typ. cons.* (*fide*Shepherd et al. 2017; Applequist 2019).

##### Description.

Perennial ***shrubs*** or ***subshrubs***, or annual or perennial ***herbs***, sometimes stoloniferous and rooting at nodes; glabrous, or with simple (sometimes multicellular) hairs, or viscid with glandular hairs. ***Leaves*** basal and/or cauline, petiolate or sessile, entire to pinnatifid, usually with axillary hairs. ***Inflorescence*** a raceme, thyrse, spike, panicle, subumbel, axillary dichasia, or flowers solitary in axils of basal leaves; pedicels sometimes articulate, rarely geniculate, with or without bracteoles. ***Sepals*** 5 or 3, fused or free, variously adnate to ovary. ***Corolla*** bilabiate or fan-like (lobes almost equal), white, cream, yellow, orange, pink, mauve, blue or purple; corolla-lobes usually winged, sometimes unequally; with hairs in the throat (rarely glabrous), sometimes with enations; often auriculate; sometimes with pouch or spur; stamens free, epigynous or hypogynous; style simple or 2–4-fid, glabrous or with simple hairs; indusia 1–4, 2-lipped, usually with bristles on lips; ovary inferior or superior, rarely winged, usually incompletely 2-locular with few to many ovules either in two rows or scattered over surface of the placentas, or solitary. ***Fruit*** a 2- or 4-valved capsule (rarely fleshy), 1-seeded nut, 4-seeded hard drupe or rarely a soft, indehiscent fruit with wings (*G. careyi*). ***Seeds*** flat or biconvex, usually with a rim or wing that is sometimes reduced.

##### Number of taxa and distribution.

The genus has c. 251 taxa and is predominantly Australian. *Goodenia pilosa* extends to New Guinea, Indonesia, Malaysia, southern China and Philippines, while *G. armstrongiana*, *G. purpurascens* and *G. pumilio* extend to New Guinea and *G. koningsbergeri* occurs in India, Thailand, Cambodia, Malaysia and Indonesia according to Karthigeyan et al. (2009). Species previously included in *Selliera* also occur in coastal habitats in New Zealand and South America.

#### Goodenia Sm. subg. Goodenia

Taxon classificationPlantaeAsteralesGoodeniaceae

2DB5FE94-625D-571F-B8C2-49414071F98C

##### Description.

***Shrubs, subshrubs*** or ***herbs***, erect, decumbent or prostrate, sometimes stoloniferous and rooting at nodes. ***Leaves*** basal, cauline or both with the upper leaves sometimes smaller and narrower. ***Flowers*** in thyrses, racemes, spikes, or flowers solitary in leaf axils; bracts usually leafy; bracteoles usually present, pedicel infrequently geniculate, articulate or not. ***Corolla*** bilabiate or fan-like, usually yellow, sometimes white, cream or blueish purple, rarely pink; throat usually with scattered hairs, often with enations, not auriculate and often with a pouch. ***Style*** simple. ***Ovary*** with a variable septum from very short to 2/3 as long as locule; ovules in 2 rows in each locule or scattered, rarely solitary. ***Fruit*** a capsule with 2 valves, persistent or deciduous, rarely a fleshy fruit or 1-seeded nut. ***Seeds*** with a wing 0.1–0.2 mm wide and mucilaginous or obsolete.

##### Number of taxa and distribution.

This subgenus currently includes 51 species, with 47 confined to Australia and three also occurring in New Zealand, Chile and southern Asia.

#### Goodenia Sm. subg. Goodenia sect. Goodenia

Taxon classificationPlantaeAsteralesGoodeniaceae

36DA7601-D91B-5AF3-939B-603904809B4B

= Selliera Cav., Anales Hist. Nat. 1(1): 41, t. 5, fig. 2. 1799 ≡ Goodenia sect. Selliera (Cav.) G.Don, Gen. hist. 3: 725. 1834 – Type: S. radicans Cav. ≡ Goodenia radicans (Cav.) Pers.
≡ Goodenia sect. Ochrosanthus G.Don, Gen. hist. 3: 724. 1834 – Type (designated by Carolin in George (ed.), Fl. Australia 35: 330. 1992): G. ovata Sm.
= Picrophyta F.Muell., Linnaea 25: 421. 1853 – Type: P. albiflora (Schltdl.) F.Muell. ≡ Goodenia albiflora Schltdl.
= Goodenia sect. Eugoodenia Benth., *Fl. Austral*. 4: 51, 57. 1868, *nom. inval.*
≡ Goodenia sect. Goodenia ser. Bracteolatae Benth., Fl. Austral. 4: 52, 59. 1868 ≡ Goodenia sect. Goodenia subsect. Bracteolatae (Benth.) K.Krause in H.G.A. Engler (ed.), Pflanzenr. 54(277): 46. 1912 – Type (designated by Carolin in George (ed.), Fl. Australia 35: 331. 1992): G. ovata Sm.
= Goodenoughia F.Muell., Syst. Census Austral. pl. 88. 1882, *nom. inval., pro syn.*
≡ Goodenoughia Siebert & Voss, Vilm. Blumengärtn. ed 3. 1(1): 559. 1894, *nom. illeg., nom. superfl.* – Type: Goodenoughia ovata (Sm.) Siebert & Voss ≡ Goodenia ovata Sm.
= Goodenia ser. Suffruticosae K.Krause in H.G.A.Engler (ed.), Pflanzenr. 54: 46, 59. 1912 – Type: G. ovata Sm.

##### Description.

***Shrubs, subshrubs*** or ***herbs***, glabrous or viscid with glandular and simple hairs. ***Leaves*** usually cauline. ***Flowers*** in leafy thyrses, racemes, spikes, or solitary in leaf axils; pedicel sometimes articulate. ***Corolla*** bilabiate or fan-like.

##### Number of taxa and distribution.

This section contains 23 species, most of which are Australian, while *G. heenanii* and *G. radicans* are present in New Zealand with the latter species also found in South America. The only other extra Australian species is *G. koningsbergeri*, which occurs in India, Thailand, Cambodia, Malaysia and Indonesia.

##### Included species.

*G. albiflora* Schltdl., *G. amplexans* F.Muell., *G. benthamiana* Carolin, *G. brunnea* Carolin, *G. calcarata* (F.Muell.) F.Muell., *G. chambersii* F.Muell., *G. collaris* (F.Muell.) K.A.Sheph., *G. exigua* F.Muell., *G. grandiflora* Sims, *G. heenanii* K.A.Sheph., *G. kingiana* Carolin, *G. koningsbergeri* (Back.) Back. ex Bold., *G. laevis* Benth., G. laevis subsp. humifusa L.W.Sage, G. laevis Benth. subsp. laevis, *G. macmillanii* F.Muell., *G. ovata* Sm., *G. phillipsiae* Carolin, *G. radicans* (Cav.) Pers., *G. saccata* Carolin, *G. stirlingii* F.M.Bailey, *G. valdentata* P.J.Lang, *G. varia* R.Br., *G. vernicosa* J.M.Black, *G. viscida* R.Br.

#### Goodenia subg. Goodenia sect. Rosulatae

Taxon classificationPlantaeAsteralesGoodeniaceae

(K.Krause) K.A.Sheph., comb. et stat. nov.

386C0C39-3DEE-55D1-8EEE-F40462B9C4FE

urn:lsid:ipni.org:names:77209991-1

= Goodenia ser. Rosulatae K.Krause in H.G.A.Engler (ed.), Pflanzenr. 54: 46, 52. 1912 – Type (designated by Carolin in George (ed.), Fl. Australia 35: 331. 1992): G. geniculata R.Br.
= Catospermum Benth., Hooker’s Icon. Pl. 11: t. 1028. 1867 – Type: C. muelleri Benth., *nom. illeg*. ≡ Goodenia goodeniacea (F.Muell.) Carolin.

##### Description.

***Herbs*** or occasionally ***subshrubs***, usually with multicellular hairs sometimes becoming glabrescent, or rarely with simple and glandular hairs. ***Leaves*** basal and/or cauline. ***Flowers*** usually in racemes or solitary in leaf axils; pedicels usually not articulate. ***Corolla*** bilabiate.

##### Number of taxa and distribution.

This section includes 28 species found in every state of Australia across a range of biomes with some species extending into arid central inland regions.

##### Included species.

*G. affinis* de Vriese, *G. arenicola* Carolin, *G. atriplexifolia* A.E.Holland & T.P. Boyle, *G. blackiana* Carolin, *G. centralis* Carolin, *G. convexa* Carolin, *G. delicata* Carolin, *G. disperma* Mueller, *G. dyeri* K.Krause, *G. expansa* A.E.Holland & T.P.Boyle, *G. fordiana* Carolin, *G. geniculata* R.Br., *G. glabra* R.Br., *G. goodeniacea* (F.Muell.) Carolin, *G. hederacea* Sm., G. hederacea subsp. alpestris (K.Krause) Carolin, G. hederacea Sm. subsp. hederacea, *G. heterophylla* Sm., G. heterophylla subsp. eglandulosa Carolin, G. heterophylla Sm. subsp. heterophylla, G. heterophylla subsp. montana Carolin, G. heterophylla subsp. teucriifolia (F.Muell.) Carolin, *G. lanata* R.Br., *G. mueckeana* F.Muell., *G. peacockiana* Carolin, *G. robusta* (Benth.) K.Krause, *G. rotundifolia* R.Br., *G. rupestris* Carolin, *G. schwerinensis* Carolin, *G. stephensonii* F.Muell., *G. tripartita* Carolin, *G. viridula* Carolin, *G. willisiana* Carolin, *G. xanthosperma* F.Muell.

#### Goodenia subg. Porphyranthus

Taxon classificationPlantaeAsteralesGoodeniaceae

(G.Don) K.A.Sheph. comb. et stat. nov.

C61A417E-DC7A-5CD0-8ADC-4D93E499078E

urn:lsid:ipni.org:names:77209992-1

≡ Goodenia sect. Porphyranthus G.Don, Gen. hist. 3: 725. 1834 – Type (designated by Carolin in George (ed.), Fl. Australia 35: 330. 1992): G. purpurascens R.Br.

##### Description.

***Subshrubs*** or ***herbs***, erect or decumbent, sometimes with a basal stock. ***Leaves*** basal, cauline or both, sometimes with smaller stem leaves. ***Flowers*** in thyrses, racemes, or subumbels, rarely head-like or solitary in axils; bracts leafy or bracteose; bracteoles present or absent; pedicels maybe articulate. ***Corolla*** bilabiate, rarely fan-like, yellow, mauve, brownish, purple, pink, or blue; throat glabrous or with long stiff hairs sometimes arranged in rows and confluent towards base, often without enations, often with auricules; pocket usually inconspicuous. ***Style*** simple or 3- or 4-fid. ***Ovary*** with septum short to 2/3 locule length; ovules in two rows in each locule or scattered over the surface of the placentas. ***Fruit*** a capsule, valves 2, persistent or deciduous, entire or bifid. ***Seeds*** with a prominent rim or a mucilaginous wing.

##### Number of taxa and distribution.

This subgenus consists of 121 species that are predominantly Australian with a few species such as *G. pumilio* and *G. armstrongiana* extending to New Guinea, while *G. pilosa* is widespread through southern Asia and China.

#### Goodenia subg. Porphyranthus sect. Porphyranthus

Taxon classificationPlantaeAsteralesGoodeniaceae

G.Don

E0BEC7AE-C62E-5577-8D64-B21A83B92447

= Goodenia sect. Amphichila DC., Prodr. 5: 516. 1836 – Type: G. pumilio R.Br.
= Neogoodenia C.A.Gardner & A.S.George, J. Roy. Soc. Western Australia 46: 138, fig. 6. 1963 – Type: N. minutiflora C.Gardner & A.S.George ≡ Goodenia neogoodenia Carolin.

##### Description.

***Herbs. Leaves*** basal, cauline or both. ***Flowers*** in thyrses, racemes, or loose panicles, rarely head-like or solitary in axils, bracts often leaf-like in lower parts becoming linear distally; pedicel usually with bracteoles. ***Corolla*** bilabiate or rarely fan-like, yellow, mauve to pinkish, or deep red, glabrous inside or with a few hairs. ***Style*** simple. ***Ovary*** with more than 30 ovules scattered over placentas. ***Seeds*** mostly less than 1 mm wide, glossy, with a prominent rim or wing *c.* 0.1 mm wide or obsolete.

##### Number of taxa and distribution.

A section of 26 species, mostly in northern and central Australia with *G. purpurascens* and *G. pumilio* also present in New Guinea. Many species are confined to seasonally wet habitats.

##### Included species.

*G. berringbinensis* Carolin, *G. bicolor* F.Muell. ex Benth., *G. chthonocephala* Carolin, *G. claytoniacea* F.Muell. ex Benth., *G. corralina* L.W.Sage & K.A.Sheph.; *G. cravenii* R.L.Barrett & M.D.Barrett, *G. cylindrocarpa* Albr., *G. gloeophylla* Carolin, *G. gracilis* R.Br., *G. gypsicola* Symon; *G. halophila* Albr., *G. humilis* R.Br., *G. kakadu* Carolin, *G. lamprosperma* F.Muell., *G. lyrata* Carolin, *G. macbarronii* Carolin, *G. minutiflora* F.Muell., *G. modesta* J.M.Black, *G. neogoodenia* Carolin, *G. nocoleche* Pellow & J.L.Porter, *G. oenpelliensis* R.L.Barrett, *G. paniculata* Sm., *G. pumilio* R.Br., *G. purpurascens* R.Br., *G. rosulata* Domin, *G. viscidula* Carolin.

#### Goodenia subg. Porphyranthus sect. Ebracteolatae

Taxon classificationPlantaeAsteralesGoodeniaceae

(K.Krause) K.A.Sheph., comb. et stat. nov.

2FE9C96F-C531-5A5E-A942-A3A7D26CAA07

urn:lsid:ipni.org:names:77209993-1

≡ Goodenia subsect. Ebracteolatae K.Krause in H.G.A.Engler (ed.), Pflanzenr. 54: 46. 1912 – Type (here designated): Goodenia pinnatifida Schltdl.
= Calogyne R.Br., Prodr. 579. 1810 ≡ Goodenia ser. Calogyne (R.Br.) Carolin, Fl. Australia 35: 331. 1992 – Type: C. pilosa R.Br. ≡ Goodenia pilosa (R.Br.) Carolin.
= Distylis Gaudich., Voy. Uranie 10: 460, t. 80. 1829 – Type: D. berardiana Gaudich. ≡ Goodenia berardiana (Gaudich.) Carolin.
= Balingayum Blanco, Fl. Filip. 187. 1837 – Type: Balingayum decumbens Blanco = Goodenia pilosa (R.Br.) Carolin.
= Aillya de Vriese, Natuurk. Verh. Holl. Maatsch. Wetensch. Haarlem ser. 2, 10: 75. 1854 – Type: A. umbellata (Vriese) Vriese = Goodenia pulchella Benth.
= Goodenia sect. Goodenia ser. Foliosae Benth., Fl. Austral. 4: 53, 69. 1868 – Type (designated by Carolin in George (ed.), Fl. Australia 35: 331. 1992): G. strangfordii F.Muell.
= Goodenia ser. Pedicellosae Benth., Fl. Austral. 4: 54, 73. 1868 – Type (designated by Carolin in George (ed.), Fl. Australia 35: 331. 1992): G. cycloptera R.Br.
= Symphyobasis K.Krause, Pflanzenr. 54: 40, fig. 11. 1912 – Type: S. macroplectra (F.Muell.) K.Krause ≡ Goodenia macroplectra (F.Muell.) Carolin.
= Goodenia subsect. Borealis Carolin ser. Borealis in A.S.George (ed.), Fl. Australia 35: 331 (1992) – Type: G. sepalosa F.Muell. ex Benth.

##### Description.

Low ***shrubs*** or ***herbs***. ***Leaves*** usually basal (sometimes ephemeral) and/or cauline. ***Flowers*** usually in racemes or subumbels with leafy bracts; pedicel usually without bracteoles. ***Corolla*** bilabiate, yellow, mauve or brownish purple, with hairs inside arranged in rows often becoming confluent towards base. ***Style*** simple or 3- or 4-fid. ***Ovary*** with 30 or less ovules, in two rows in each locule. ***Seeds*** usually more than 1.5 mm wide, rarely glossy, wing prominent and usually mucilaginous.

##### Number of taxa and distribution.

This section includes 95 species across Australia with *G. armstrongiana* extending northwards into New Guinea. The annual G. pilosa subsp. pilosa extends from Australia to Indonesia, Papua New Guinea, Malaysia, and the Philippines while the perennial G. pilosa subsp. chinensis is found in China and Vietnam.

##### Included species.

*G. anfracta* J.M.Black, *G. angustifolia* Carolin, *G. arachnoidea* Carolin, *G. argillacea* Carolin, *G. armitiana* F.Muell, *G. armstrongiana* de Vriese, *G. asteriscus* P.J.Lang, *G. berardiana* (Gaudich.) Carolin, *G. brachypoda* (F.Muell. ex Benth.) Carolin, *G. byrnesii* Carolin, *G. campestris* Carolin, *G. cirrifica* F.Muell., *G. concinna* Benth., *G. coronopifolia* R.Br., *G. corynocarpa* F.Muell.; *G. crenata* Carolin & L.W.Sage; *G. cusackiana* F.Muell., *G. cycloptera* R.Br., *G. debilis* A.E.Holland & T.P.Boyle; *G. durackiana* Carolin; *G. effusa* A.E.Holland, *G. elaiosoma* Cowie; *G. elongata* Labill., *G. fascicularis* F.Muell. & Tate; *G. faucium* Carolin; *G. filiformis* R.Br., *G. forrestii* F.Muell., *G. gibbosa* Carolin, *G. glandulosa* K.Krause, *G. glauca* F.Muell., *G. granitica* L.W.Sage & K.A.Sheph., *G. havilandii* Maiden & Betche, *G. heatheriana* L.W.Sage, *G. heppleana* (W.Fitzg.) Carolin, *G. heterochila* F.Muell., *G. heteromera* F.Muell., *G. heterotricha* M.D.Barrett & R.L.Barrett, *G. hirsuta* F.Muell., *G. hispida* R.Br., *G. holtzeana* (Specht) Carolin, *G. integerrima* Carolin, *G. inundata* L.W.Sage & J.P.Pigott, *G. iyouta* Carolin, *G. janamba* Carolin, *G. jaurdiensis* L.W.Sage & K.A.Sheph., *G. krauseana* Carolin, *G. larapinta* Tate, *G. leiosperma* Carolin, *G. lobata* Ising, *G. lunata* J.M.Black, *G. macroplectra* (F.Muell.) Carolin, *G. maideniana* W.Fitzg., *G. malvina* Carolin, *G. maretensis* R.L.Barrett, *G. megasepala* Carolin, *G. micrantha* Hemsl. ex Carolin, *G. microptera* F.Muell., *G. mimuloides* S.Moore, *G. muelleriana* Carolin, *G. neglecta* (Carolin) Carolin, *G. nigrescens* Carolin, *G. nuda* E.Prtiz., *G. occidentalis* Carolin, *G. ochracea* Carolin, *G. odonnellii* F.Muell., *G. pallida* Carolin, *G. pascua* Carolin, *G. pedicellata* L.W.Sage & K.W.Dixon, *G. pilosa* (R.Br.) Carolin, G. pilosa (R.Br.) Carolin subsp. pilosa, G. pilosa subsp. chinensis (Benth.) D.G.Howarth & D.Y.Hong, *G. pinnatifida* Schltdl., *G. porphyrea* (Carolin) Carolin, *G. potamica* Carolin, *G. pritzelii* Domin, *G. prostrata* Carolin, *G. psammophila* L.W.Sage & M.D.Barrett, G. psammophila subsp. hiddinsiana L.W.Sage & M.D.Barrett, G. psammophila L.W.Sage & M.D.Barrett subsp. psammophila, *G. pulchella* Benth., *G. purpurea* (F.Muell.) Carolin, *G. pusilla* (de Vriese) de Vriese, *G. pusilliflora* F.Muell., *G. quadrifida* (Carolin) Carolin, *G. quasilibera* Carolin, *G. redacta* Carolin, *G. salina* L.W.Sage & K.A.Sheph., *G. salmoniana* (F.Muell.) Carolin, *G. sepalosa* F.Muell. ex Benth., G. sepalosa var. glandulosa Carolin, G. sepalosa F.Muell. ex Benth. var. sepalosa, *G. stellata* Carolin, *G. strangfordii* F.Muell., *G. subauriculata* C.T.White, *G. symonii* (Carolin) Carolin, *G. tenuiloba* F.Muell., *G. triodiophila* Carolin, *G. turleyae* L.W.Sage & K.A.Sheph., *G. vilmoriniae* F.Muell., *G. virgata* Carolin, *G. wilunensis* Carolin.

#### Goodenia subg. Monochila

Taxon classificationPlantaeAsteralesGoodeniaceae

(G.Don) Carolin, Fl. Australia 35: 330. 1992.

3F16FFA2-8A6F-5A4F-B456-F8CC4D6B0B58

= Goodenia sect. Monochila G.Don, Gen. hist. 3: 725. 1834.

##### Type.

(designated by Carolin in George (ed.), Fl. Australia 35: 330. 1992): *G. scapigera* R.Br.

##### Description.

***Shrubs, subshrubs*** or ***herbs***, erect or decumbent, sometimes with a basal stock. ***Leaves*** basal, cauline or both. ***Flowers*** in axillary dichasia or terminal thyrses, racemes or spikes; bracts leafy or bracteose; bracteoles present; pedicels may be articulate. ***Corolla*** bilabiate or fan-like, white (sometimes with purple spots at the base of the lobes), yellow, blue or blueish-purple or rarely pink to mauve; throat glaborous, sometimes with stiff hairs, with or without enations, with or without auricles; pouch inconspicuous or prominent to 1/2 ovary length. ***Style*** simple. ***Ovary*** with septum *c.* 2/3 locule length or 1 locular; ovules usually in 2-rows, rarely solitary. ***Fruit*** a capsule with 2 valves, persistent or deciduous, rarely indehiscent and nutlike. ***Seeds*** with or without a membranous or mucilaginous wing.

##### Number of taxa and distribution.

The subgenus Monochila includes 58 species across six sections. Western Australia is a centre of diversity for this group with species from sect. Velleia also found in Eastern Australia and one species present in New Guinea.

#### Goodenia subg. Monochila sect. Monochila

Taxon classificationPlantaeAsteralesGoodeniaceae

G.Don, Gen. hist. 3: 725. 1834.

46B9DC76-BD08-578E-85C0-3B9C719FFF94

##### Description.

***Shrubs, subshrubs*** or ***herbs***. ***Leaves*** basal, cauline or both. ***Flowers*** in thyrses or spikes; usually with bracteose bracts; bracteoles present. ***Sepals*** equal. ***Corolla*** fan-like, white with brown or purple spots at the base of each lobe or pink with a bright yellow throat, with stiff hairs in throat, enations absent, without auricles; pouch inconspicuous, to 1/2 ovary length. ***Ovary*** 2-locular with ovules solitary or to 40, usually in 2-rows in each locule. ***Fruit*** either a capsule with valves bifid or indehiscent and nutlike. ***Seeds*** to c.1 mm, wing < 0.5 mm and mucilaginous or obsolete.

##### Number of taxa and distribution.

A Western Australian section of 10 species.

#### Goodenia subg. Monochila

Taxon classificationPlantaeAsteralesGoodeniaceae

sect. Monochila subsect. Monochila

174DA39E-C177-5F70-83E7-599D102081A3

= Stekhovia de Vriese, Natuurk. Verh. Holl. Maatsch. Wetensch. Haarlem ser. 2, 10: 166. 1854. – Type: S. scapigera (R.Br.) de Vriese ≡ Goodenia scapigera R.Br.

##### Description.

***Style*** with simple white hairs. ***Ovary*** with 12–40 ovules. ***Fruit*** a cylindrical to ovoid capsule.

##### Number of taxa and distribution.

A subsection of six species endemic to south-west Western Australia.

##### Included species.

*G. decursiva* W.Fitzg., *G. elderi* F.Muell. & Tate, *G. pinifolia* de Vriese, *G. scapigera* R.Br., G. scapigera subsp. graniticola L.W.Sage, G. scapigera R.Br. subsp. scapigera, *G. sericostachya* C.A.Gardner, *G. watsonii* F.Muell. & Tate, G. watsonii subsp. glandulosa Carolin, G. watsonii F.Muell. & Tate subsp. watsonia.

#### Goodenia subg. Monochila sect. Monochila subsect. Infracta

Taxon classificationPlantaeAsteralesGoodeniaceae

K.A.Sheph. subsect. nov.

08F5DDE4-6516-5767-B210-9E401BE3FBF1

= Scaevola ser. Parviflorae Benth., Fl. Austral. 4: 86, 103. 1868 – Type: S. fasciculata Benth. ≡ Goodenia fasciculata (Benth.) Carolin.

##### Type.

*G. helmsii* (E.Pritz.) Carolin.

##### Description.

***Style*** with stiff purple hairs. ***Ovary*** with 1–3 ovules. ***Fruit*** a globular to subglobular nut.

##### Etymology.

The name is from the Latin *infractus* (unbroken) in reference to members of this section having a hard, nut-like fruit.

##### Number of taxa and distribution.

A subsection of four species endemic to south-west Western Australia.

##### Included species.

*G. drummondii* Carolin, G. drummondii Carolin subsp. drummondii, G. drummondii subsp. megaphylla L.W.Sage, *G. fasciculata* (Benth.) Carolin, *G. helmsii* (E.Pritz.) Carolin, *G. stenophylla* F.Muell.

#### Goodenia subg. Monochila sect. Scaevolina

Taxon classificationPlantaeAsteralesGoodeniaceae

(Carolin) K.A.Sheph., comb. et stat. nov.

381E6B37-2BB5-57C3-AA07-33202EF937D2

urn:lsid:ipni.org:names:77209994-1

≡ Goodenia subsect. Scaevolina Carolin in A.S.George (ed.), Fl. Austral. 35: 331. 1992 – Type: G. scaevolina F.Muell.

##### Description.

***Subshrubs*** or ***herbs***. ***Leaves*** basal or cauline, basal leaves sometimes absent in mature plants. ***Flowers*** in thyrses or racemes comprising at least 1/2 to 2/3 of the plant, with leafy bracts; bracteoles present. ***Sepals*** equal. ***Corolla*** bilabiate or becoming fan-like, blue usually with a yellow or whitish throat, usually with hairs on margins and in the throat, enations prominent; scarcely auriculate; pouch usually prominent. ***Ovary*** 2-locular with 20–60 ovules in two rows in each locule. ***Fruit*** a capsule, valves entire or bifid. ***Seeds*** > 1.5 mm wide, wing *c.* 0.1 mm wide and mucilaginous or obsolete.

##### Number of taxa and distribution.

A section of eight species from northern and central Australia extending southwards into the Eremaean bioregion of Western Australia.

##### Included species.

*G. azurea* F.Muell., Goodenia azurea F.Muell. subsp. azurea, G. azurea subsp. hesperia L.W.Sage & Albr., *G. eremophila* E.Pritz., *G. hartiana* L.W.Sage, *G. ramelii* F.Muell., *G. scaevolina* F.Muell., *G. splendida* A.E.Holland & T.P.Boyle, *G. stobbsiana* F.Muell., *G. suffrutescens* Carolin.

#### Goodenia subg. Monochila sect. Coeruleae

Taxon classificationPlantaeAsteralesGoodeniaceae

(Benth.) Carolin in A.S.George (ed.), Fl. Australia 35: 330. 1992 (as ‘Caeruleae’)

EB1A037B-6080-5506-B2DD-C08E585980F0

≡ Goodenia subg. Goodenia ser. Coeruleae Benth., Fl. Austral. 4: 53, 65. 1868 (as ‘Caeruleae’) ≡ Goodenia subg. Goodenia subsect. Coeruleae (Benth.) Carolin, Fl. Australia 35: 330. 1992 (as ‘Caeruleae’) – Type (designated by Carolin in George (ed.), Fl. Australia 35: 330. 1992): G. coerulea R.Br.

##### Description.

***Herbs*** with basal stock. ***Leaves*** basal or cauline. ***Flowers*** in racemes arising from axils of basal leaves with bracteose or leafy bracts; bracteoles present. ***Sepals*** equal. ***Corolla*** bilabiate; blue usually with a yellow or whitish throat, usually with hairs in the throat, enations present or absent; scarcely auriculate; pouch present or absent. ***Ovary*** 2-locular with 20–40 ovules in two rows in each locule. ***Fruit*** a capsule, valves entire or bifid. ***Seeds*** > 1.5 mm wide, wing > 0.1 mm wide dry, hyaline or obsolete.

##### Number of taxa and distribution.

A section of 11 species from south-west Western Australia.

##### Included species.

*G. coerulea* R.Br., *G. eatoniana* F.Muell., *G. glareicola* Carolin, *G. hassallii* F.Muell., *G. incana* R.Br., *G. katabudjar* Cranfield & L.W.Sage, *G. lancifolia* L.W.Sage & Cranfield, *G. leptoclada* Benth., *G. perryi* C.A.Gardner ex Carolin, *G. pterigosperma* R.Br., *G. trichophylla* de Vriese ex Benth.

#### Goodenia subg. Monochila sect. Tetrathylax

Taxon classificationPlantaeAsteralesGoodeniaceae

G.Don, Gen. hist. 3: 725. 1834

B1CA7B52-078B-53C1-85DE-09F64425515A

≡ Tetrathylax (G.Don) de Vriese, Natuurk. Verh. Holl. Maatsch. Wetensch. Haarlem ser. 2, 10: 164. 1854 (as ‘Tetraphylax’) – Type: T. quadrilocularis (R.Br.) Vriese ≡ Goodenia quadrilocularis R.Br.
= Goodenia sect. Goodenia ser. Racemosae Benth., Fl. Austral. 4: 51, 57 (1868) – Type (designated by Carolin in George (ed.), Fl. Australia 35: 331. 1992): G. decurrens R.Br.

##### Description.

***Subshrubs*** or ***herbs***. ***Leaves*** basal or if cauline usually narrower and smaller. ***Flowers*** in thyrse-like panicles, racemes or spikes with bracteose bracts; bracteoles present. ***Sepals*** equal. ***Corolla*** bilabiate, yellow, lemon or rarely orange, hairs in throat, enations present; scarcely auriculate to auriculate. ***Ovary*** 2-locular with 15–65 ovules in two rows in each locule. ***Fruit*** a capsule, valves usually + bifid or rarely entire. ***Seeds*** 1–1.8 mm long, wing c. 0.1 mm wide and mucilaginous or with a rim.

##### Number of taxa and distribution.

A section of nine species present in coastal and highland areas including the Blue Mountains in eastern Australia and *G. quadrilocularis* from near Cape le Grand in south-west Western Australia.

##### Included species.

*G. bellidifolia* Sm., G. bellidifolia subsp. argentea Carolin, G. bellidifolia Sm. subsp. bellidifolia, *G. decurrens* R.Br., *G. dimorpha* Maiden & Betche, G. dimorpha var. angustifolia Maiden & Betche, G. dimorpha Maiden & Betche var. dimorpha, *G. glomerata* Maiden & Betche, *G. lineata* J.H.Willis, *G. quadrilocularis* R.Br., *G. racemosa* F.Muell., G. racemosa var. latifolia Carolin, G. racemosa F.Muell. var. racemosa, *G. rostrivalvis* Domin, *G. stelligera* R.Br.

##### Note.

Don (1834) recognised the section Tetrathylax (meaning four – pouch) to include *G. quadrilocularis*. The name was formed providing the Greek and Latin translations for *tetras* (four-fold) and *thylax* (a cell) in recognition of the 4-celled condition of the capsule. de Vriese treated the section at generic rank with the incorrect spelling ‘*Tetraphylax*’, which was followed by subsequent authors until corrected by Carolin (1992e).

#### Goodenia subg. Monochila sect. Verreauxia

Taxon classificationPlantaeAsteralesGoodeniaceae

(Benth.) K.A.Sheph., comb. et. stat. nov.

D1C04659-DAEE-5B0F-82EC-5786B5FB1FA3

urn:lsid:ipni.org:names:77210076-1

≡ Verreauxia Benth., Fl. Austral. 4: 105. 1868, syn. nov. – Type: V. verreauxii (de Vriese) Carolin = Dampiera verrauxii de Vriese.
= Pentaptilon E.Pritz., Bot. Jahrb. Syst. 35(4): 564, fig. 65. 1905, syn. nov. – Type: P. careyi (F.Muell.) Pritzel ≡ Catospermum careyi F.Muell. ≡ Goodenia careyi (F.Muell.) K.A.Sheph.

##### Description.

***Herbs or shrubs***. ***Leaves*** basal and/or cauline, with unique branched hairs. ***Flowers*** in loose or spike-like, often a branched thyrse on a terminal scape with bracteose bracts; bracteoles present. ***Sepals*** equal. ***Corolla*** bilabiate, yellow, with or without hairs inside throat, enations absent; scarcely auriculate; pouch inconspicuous to short. ***Ovary*** 1- to 2-locular, with unique reddish or golden multicellular hairs between 3 of the sepaline ribs that may be winged (in *G. careyi*), with 1–6 ovules per locule scattered over placentas. ***Fruit*** a compressed, hairy nut or an indehiscent capsule with wings. ***Seeds*** 1.7–2.5 mm long, wing obsolete.

##### Number of taxa and distribution.

A section of four species endemic to south-western Australia.

##### Included species.

*G. careyi* (F.Muell.) K.A.Sheph., *G. etheira* K.A.Sheph., *G. reinwardtii* (de Vriese) K.A.Sheph., *G. verreauxii* (de Vriese) K.A.Sheph.

#### Goodenia subg. Monochila sect. Velleia

Taxon classificationPlantaeAsteralesGoodeniaceae

(Sm.) K.A.Sheph., comb. et. stat. nov.

55B303A0-3BA7-5059-8367-DE36EB216933

urn:lsid:ipni.org:names:77210077-1

≡ Velleia Sm., Trans. Linn. Soc. London, Bot. 4: 217. 1798, syn. nov. – Type: V. lyrata R.Br.
= Euthales R.Br., Prodr. 579. 1810 ≡ Velleia sect. Euthales (R.Br.) Carolin, Proc. Linn. Soc. New South Wales 92(1): 28. 1967 – Type: E. trinervis (Labill.) R.Br. ≡ Goodenia trinervis (Labill.) K.A. Sheph.
= Menoceras (R.Br.) Lindl., Veg. kingd. 685. 1846 ≡ Velleia sect. Menoceras R.Br., Prodr. 580. 1810 – Type: (designated by Carolin, Proc. Linn. Soc. New South Wales 92(1): 34. 1967): Velleia paradoxa R.Br. ≡ Goodenia paradoxa (R.Br.) K.A.Sheph.
= Velleia sect. Aceratia F.Muell., Trans. Philos. Soc. Victoria 1: 17. 1854 – Type: V. connata F.Muell. ≡ Goodenia connata (F.Muell.) K.A.Sheph.
= Antherostylis C.A.Gardner, J. Roy. Soc. Western Australia 19: 91. 1934 – Type: A. calcarata C.Gardner ≡ G. arguta (R.Br.) K.A. Sheph.

##### Description.

***Herbs*** with short stems. ***Leaves*** basal or cauline. ***Flowers*** in axillary dichasia with scapes erect to prostrate or flowers solitary in axils usually with bracteose bracts; bracteoles present and sometimes disc-like. ***Sepals*** equal or adaxial one larger. ***Corolla*** bilabiate yellow, orange, mauve, pink or white, with or without hairs in the throat, enations absent or present, auriculate, pouch absent or present, sometimes forming a spur. ***Ovary*** 2-locular with 4–25 ovules. ***Fruit*** a capsule with with 2 or 4 valves. ***Seeds*** 1.5–6 mm long, wing 0.5–2 mm wide or with a thickened rim.

##### Number of taxa and distribution.

The section includes 20 species, of which 19 are endemic to Australia with *G. mystrophylla* K.A.Sheph. (previously *Velleia spathulata*) also present in New Guinea.

##### Included species.

*G. arguta* (R.Br.) K.A.Sheph., *G. brendannarum* K.A.Sheph., *G. capillosa* K.A.Sheph., *G. caroliniana* K.A.Sheph., *G. connata* (F.Muell.) K.A.Sheph., *G. cycnopotamica* (F.Muell.) K.A.Sheph., *G. daviesii* (F.Muell.) K.A.Sheph., *G. discophora* (F.Muell.) K.A.Sheph., *G. subsolana* K.A.Sheph., *G. glabrata* (Carolin) K.A.Sheph., *G. macrocalyx* (de Vriese) K.A.Sheph., *G. macrophylla* (Lindl.) F.Muell., *G. montana* (Hook.f.) K.A.Sheph., *G. mystrophylla* K.A.Sheph., *G. panduriformis* (A.Cunn. ex Benth.) K.A.Sheph., *G. paradoxa* (R.Br.) K.A.Sheph., *G. parvisepta* (Carolin) K.A.Sheph., *G. perfoliata* (R.Br.) K.A.Sheph., *G. rosea* (S.Moore) K.A.Sheph., *G. trinervis* (Labill.) K.A.Sheph.

### Incertae sedis

*Goodenia arthrotricha* Benth., Fl. Austral. 4: 62. 1868 – Lectotype (designated by Carolin, Telopea 3(4): 539. 1990): Australia. Western Australia. S.W. Australia, 1848, *J.Drummond 190* (K 000215740 [image!]; isolectotype: BM 001041473 [image!]; probable isolectotype: MEL 23036 [image!], MEL 23037 [image!]).

*Goodenia xanthotricha* de Vriese, Natuurk. Verh. Holl. Maatsch. Wetensch. Haarlem ser. 2, 10: 155. 1854 – Type citation: “Nov. Holl. Verreaux. (*Herb. propr.*)”. Type: *n.v*.

### New Combinations and reinstated taxa

#### Goodenia arguta

Taxon classificationPlantaeAsteralesGoodeniaceae

(R.Br.) K.A. Sheph. comb. nov.

B5ED1F6D-C2E6-548E-8A16-2C10090F4DBF

urn:lsid:ipni.org:names:77210078-1

≡ Velleia arguta R.Br., Prodr. 580. 1810 – Holotype: Australia: Western Australia. Base of the Mountains near Inlet No. XII South Coast, *s. dat.*, *R.Brown s.n*. [Bennett no. 2548] (BM 000949843 [image!]).

#### Goodenia brendannarum

Taxon classificationPlantaeAsteralesGoodeniaceae

K.A.Sheph. nom. nov.

4F72C072-DBB7-5BB7-83AA-132E7383D5C0

urn:lsid:ipni.org:names:77210079-1

≡ Velleia macrophylla var. foliosa Benth. Fl. Austral. 4: 48. 1868 ≡ Velleia foliosa (Benth.) K.Krause in H.G.A.Engler (ed.), Pflanzenr. 54: 40. 1912 – Lectotype (designated by Carolin, Proc. Linn. Soc. New South Wales 92(1): 33 (1967): Australia. Western Australia. S.W. Australia, *J.Drummond 182* (K 000215384 [image!]; isolectotypes: MEL 9736 [image!], NSW *n.v.*, P 00698807 [image!], P 00698808 [image!])).

##### Note.

The epithet ‘foliosa’ is unavailable in *Goodenia* as it is preoccupied by *Goodenia foliosa* (F.Muell. ex Benth.) Domin (= *G. decursiva* W.Fitzg.).

##### Etymology.

This species commemorates Australian botanists Brendan Lepschi (1969–) and Anna Monro (1974–), in recognition of their tireless service to the botanical community through providing expert nomenclatural and taxonomic advice and maintenance of the *Australian Plant Name Index* (https://biodiversity.org.au/nsl/services/APNI), a truly invaluable resource that lists published Australian vascular plant names and key citations in the literature.

#### Goodenia capillosa

Taxon classificationPlantaeAsteralesGoodeniaceae

K.A.Sheph. nom. nov.

E8025213-947D-59D6-92C4-D9E347063647

urn:lsid:ipni.org:names:77210080-1

≡ Velleia hispida W.Fitzg., *W. Austral. Nat. Hist. Soc.* 1: 25 (1904) – Lectotype (designated by Carolin, Proc. Linn. Soc. New South Wales 92(1): 42 (1967): Nannine, *W.V.Fitzgerald*, Sept. 1903 (NSW 75661, *n.v.*); isolectotype: Australia. Western Australia. Nannine, Sep. 1903, [*W.V.Fitzgerald s.n.*] (PERTH 01639986!)).

##### Note.

The epithet ‘hispida’ is unavailable in *Goodenia* as it is preoccupied by *G. hispida* R.Br.

##### Etymology.

Named from the Latin *capillosus* (hairy) in reference to the indumentum present on the leaves and sepals.

#### Goodenia careyi

Taxon classificationPlantaeAsteralesGoodeniaceae

(F.Muell.) K.A.Sheph. comb. nov.

8FD6DE2A-3F19-56A1-B2D2-5BD0F36A4CD7

urn:lsid:ipni.org:names:77210081-1

≡ Catospermum careyi F.Muell., Australas. Chem. Druggist 6: 96. 1884 – Holotype: Australia: Western Australia. Between Northampton and Shark Bay, 1884, *S.Carey s.n.* (MEL 2192442 [image!]). ≡ Pentaptilon careyi (F.Muell.) Pritzel, Bot. Jahrb. Syst. 35(4): 564, fig. 65. 1905.

#### Goodenia caroliniana

Taxon classificationPlantaeAsteralesGoodeniaceae

K.A.Sheph. nom. nov.

2AF7E61A-12E0-52E4-962E-5AE4BAC72749

urn:lsid:ipni.org:names:77210082-1

≡ Velleia lyrata R.Br., Prodr. 580. 1810 – Lectotype (designated by Carolin, Proc. Linn. Soc. New South Wales 92(1): 48 (1967): Australia. New South Wales. South Head of Port Jackson, 1803, *R.Brown s.n.* ([Bennett no. 2549] (BM 001041389 [image!]); isolectotypes: BM 001041387 [image!], BM 001041388 [image!], BM 001041390 [image!], CANB 279052!, G-DC 00322630 [image!], K 000215413 [image!]; K 00215414 [image!], K 00215415 [image!], MEL 9713 [image!], P 00698809 [image!], P 00698810 [image!]).

##### Note.

The epithet ‘lyrata’ is unavailable in *Goodenia* as it is preoccupied by *G. lyrata* Carolin.

##### Etymology.

This species is endemic to the Sydney region of New South Wales and is named in honour of Roger Charles Carolin (1929–), an Associate Professor at the University of Sydney and Curator of the John Ray Herbarium (SYD) for more than 30 years. During his tenure Carolin published numerous treatments including revision of the family Goodeniaceae for the *Flora of Australia*.

#### Goodenia collaris

Taxon classificationPlantaeAsteralesGoodeniaceae

(F.Muell.) K.A.Sheph. comb. nov.

6CC44E8E-0B98-510F-81A5-59CB9944AE55

urn:lsid:ipni.org:names:77210083-1

≡ Scaevola collaris F.Muell., Rep. pl. Babbage’s Exped. 15 (1859) – Type citation: “On sand ridges near Wonnomulla.” Possible syntype: Australia. South Australia. Lake Eyre, *s. dat., leg. ign. s.n.* (AD 97604803 [image!]).

##### Note.

Carolin (1992c) cited the type of the name *Scaevola collaris* F.Muell. as “Near Wonnamulla, S.A., *Babbage Expedition*; holo: MEL.” A specimen of *S. collaris* labelled “NW interior of South Australia, 1859” (*J.M.Stuart s.n.*, MEL 1520987A, *n.v.*) is held at MEL (N.G. Walsh, pers. comm. 2019), but this is unlikely to represent original material of this name, and Carolin’s text may be an interpretation of the type citation rather than label data from any physical specimens. Similarly, a specimen of *S. collaris* at K labelled “Mr McDougal [*sic*; McDouall] Stuart’s journey of 1859 to the interior of Australia” (K 000216199 [image!])), is also unlikely to represent original material, as noted by Roger Carolin’s 1973 annotation on the specimen. Both specimens at K and MEL are likely to have been collected during McDouall Stuart’s second or third expeditions to northern South Australia during 1859, rather than during the Babbage expedition of 1858 (see Morris 1976 and Symes 1969, respectively). A collection at AD (AD 97604803) may represent original material of this name. The collection comprises two flowering branchlets of *Goodenia collaris*, is labelled “Scaevola collaris F.v.Muell. / Lake Eyre” in an unknown hand, and matches the description given in the protologue, with the exception of fruits, which are absent from this material. The AD material is here treated as a possible syntype, in the absence of conclusive information as to its exact origin and history at this time.

#### Goodenia connata

Taxon classificationPlantaeAsteralesGoodeniaceae

(F.Muell.) K.A.Sheph. comb. nov.

5605C97F-2054-5570-B381-A1EC152DC237

urn:lsid:ipni.org:names:77210084-1

≡ Velleia connata F.Muell., Trans. Philos. Soc. Victoria 1: 18. 1855 – Holotype: Australia. Sandhills towards the junction of the Murray & Murrumbidgee, Dec. 1853, *F. von Mueller s.n.* (MEL 594385 [image!]).

##### Note.

Carolin (1967c) cites a Mueller collection at K (K 00215370) as the “Holo (?) type”, and states “There is no specimen in MEL corresponding to this and it is assumed the holotype was sent to, and retained by K”. Subsequently, Carolin (1992g) treated this same collection as an “iso[type]”, and it is annotated by Carolin as “part of the HOLOTYPE.” Carolin does not appear to have seen MEL 594385, treated here as the holotype of this name. The MEL specimen is a good match for the protologue, including the locality statement. The specimen at K, referred to and examined by Carolin, represents *Goodenia connata* and is of a similar developmental state as the specimen at MEL. It bears a label in Mueller’s hand reading “Velleya (Aceratia) connata FvMueller, Murray Scrub”. However, it is not certain whether this specimen is part of the original material, and it is therefore not considered for purposes of typification of this name.

#### Goodenia cycnopotamica

Taxon classificationPlantaeAsteralesGoodeniaceae

(F.Muell.) K.A.Sheph. comb. nov.

B5458A19-B95F-560F-B5CA-4B3BA27AA7F9

urn:lsid:ipni.org:names:77210085-1

≡ Velleia cycnopotamica F.Muell., Fragm. 6: 7. 1867 – Lectotype (designated by Carolin, Proc. Linn. Soc. New South Wales 92(1): 41 (1967): Australia. Western Australia. Without precise locality, *s. dat.*, *J.Drummond 410* (MEL 9798 [image!]; isolectotypes: G 00355707 [image!], P 00689747 [image!], P 00698811 [image!])).

##### Note.

Carolin (1967c) cited the type of the name *Velleia cycnopotamica* F.Muell. as “*Holotype* – Ad flumen cygnorum. Drummond no. 410 (MEL 9798) – *Isotypes* – (P.G).” This is here treated as effective lectotypification by Carolin. As Carolin’s citation meets the relevant requirements of ICN Art. 7.11, his use of the terms “holotype” and “isotype” is correctable under ICN Art. 9.10.

#### Goodenia daviesii

Taxon classificationPlantaeAsteralesGoodeniaceae

(F.Muell.) K.A.Sheph. comb. nov.

5B8045C6-D4A2-5690-8317-99F16E06EAA3

urn:lsid:ipni.org:names:77210120-1

≡ Velleia daviesii F.Muell., Fragm. 10: 10. 1876 – Holotype: Australia. Western Australia. Near Ularing, 1875, *Young s.n.* (MEL 9647 [image!]).

#### Goodenia discophora

Taxon classificationPlantaeAsteralesGoodeniaceae

(F.Muell.) K.A.Sheph. comb. nov.

B595E1CC-B650-5A1A-A0E4-C60C64EAC830

urn:lsid:ipni.org:names:77210086-1

≡ Velleia discophora F.Muell., Fragm. 10: 10. 1876 – Holotype: Australia. Western Australia. Near Ularing, 10–15 Oct 1875, *Young s.n.* (MEL 9649 [image!]).

#### Goodenia etheira

Taxon classificationPlantaeAsteralesGoodeniaceae

K.A.Sheph. nom. nov.

5DB740A4-8817-575A-889F-2CDF05B794FF

urn:lsid:ipni.org:names:77210087-1

≡ Verreauxia dyeri E.Pritz. ex Hemsl., Hooker’s Icon. Pl. 28: t. 2782. 1905 – Lectotype (designated by Carolin in A.S.George (ed.), Fl. Austral. 35: 103. 1992: Australia. Western Australia. Railway between Cunderdin and Dedari, 1903, *G.H.Thiselton-Dyer 105* (K 00216471 [image!])).

##### Note.

The epithet ‘dyerib’ is unavailable in *Goodenia* as it is preoccupied by *Goodenia dyeri* Krause.

##### Etymology.

This species is named for the Greek *etheira* (hair, mane) in reference to the villous hairs on the leaves.

#### Goodenia exigua

Taxon classificationPlantaeAsteralesGoodeniaceae

F.Muell., Fragm. 3(22): 142. 1863

9C3EAE0D-0221-5F6A-A172-B67F630F21F9

≡ Selliera exigua (F.Muell.) Benth., Fl. Austral. 4: 82. 1868 ≡ Velleia exigua (F.Muell.) Carolin in A.S.George (ed.), Fl. Australia 35: 334. 1992 – Lectotype (designated by Carolin in A.S.George (ed.), Fl. Australia. 35: 331. 1992): Australia. Western Australia. Moirs Inlet, *s. dat.*, [*G.Maxwell s.n.*] (MEL 24156 [image!]; isolectotype: K 000216089 [image!]).

#### Goodenia glabrata

Taxon classificationPlantaeAsteralesGoodeniaceae

(Carolin) K.A.Sheph. comb. nov.

23C95D16-CA63-509C-A262-9382409C9519

urn:lsid:ipni.org:names:77210088-1

≡ Velleia glabrata Carolin, Proc. Linn. Soc. New South Wales 92: 46. 1967 – Holotype: Australia. Queensland. Urimbin, South of Thargomindah, 16 Aug 1964, *R.C.Carolin 4080* (NSW 100797, *n.v.*).

#### Goodenia heenanii

Taxon classificationPlantaeAsteralesGoodeniaceae

K.A.Sheph. nom. nov.

0844E8FC-C605-527C-9613-A88ED2233F17

urn:lsid:ipni.org:names:77210089-1

≡ Selliera rotundifolia Heenan, New Zealand J. Bot. 35: 133–137. 1997 – Holotype: New Zealand. Manawatu, Hokio Beach, sand plain behind foredunes, 30 Jan 1996, *P.B.Heenan 4/96* (CHR 507535; isotypes: AK *n.v.*, WELT 81947 [image!]).

##### Note.

The epithet ‘rotundifolia’ is unavailable in *Goodenia* as it is preoccupied by *Goodenia rotundifolia* R.Br.

##### Etymology.

Named in honour of the highly respected New Zealand botanist Peter Heenan (1961–), who first recognised this species as distinct.

#### Goodenia macrocalyx

Taxon classificationPlantaeAsteralesGoodeniaceae

(de Vriese) K.A.Sheph. comb. nov.

4F8B3B2B-5E9B-5669-A23D-D5CE4EC16AF5

urn:lsid:ipni.org:names:77210090-1

≡ Velleia macrocalyx de Vriese, in T.L.Mitchell, J. exped. trop. Australia: 258. 1848 – Holotype: Australia. Sub-Tropical New Holland, 1846, *T.L.Mitchell 237* (L 0001763 [image!]).

#### Goodenia macrophylla

Taxon classificationPlantaeAsteralesGoodeniaceae

(Lindl.) F.Muell., Fragm. 6(41): 11. 1867

6FE582F0-41EC-510C-BE0E-51C1A889E497

≡ Euthales macrophylla Lindl., Edward’s Bot. Reg. 26: 54 (1840)
≡ Velleia macrophylla (Lindl.) Benth., Fl. Austral. 4: 47. 1868 – Lectotype (designated by Carolin, Proc. Linn. Soc. New South Wales 92(1): 34 (1967): “(ex) Hort. Soc. Nat. Lond. Grown from seed purchased of James Drummond 1840” (CGE, *n.v.*; isolectotype: K, *n.v.*)).

#### Goodenia montana

Taxon classificationPlantaeAsteralesGoodeniaceae

(Hook.f.) K.A.Sheph. comb. nov.

1E7403B9-DFBC-5454-85E7-635ED6ABBB0F

urn:lsid:ipni.org:names:77210091-1

≡ Velleia montana Hook.f., Hooker’s London J. Bot. 6: 265. 1847 – Lectotype (designated by Carolin, Proc. Linn. Soc. New South Wales 92(1): 56 (1967): Australia. Tasmania. Hampshire Hills, Feb 1837, *R.C.Gunn 227* (K 000215445 [image!])).

#### Goodenia mystrophylla

Taxon classificationPlantaeAsteralesGoodeniaceae

K.A.Sheph. nom. nov.

B72CDD8F-96DD-5616-A13E-5B0212D860AC

urn:lsid:ipni.org:names:77210092-1

≡ Velleia spathulata R.Br., Prodr. 580. 1810 – Lectotype (designated by Carolin, Proc. Linn. Soc. New South Wales 92(1): 51 (1967): Australia: New South Wales. …prope Kingstown Newcastle, Oct – Nov 1804, *R.Brown s.n*. (BM 001041385 *p.p.* [image!]); isolectotype: MEL 9776 *p.p.* [image!])).

##### Note.

The epithet ‘spathulata’ is unavailable in *Goodenia* as it is preoccupied by *Goodenia spathulata* de Vriese (= *G. bellidifolia* Sm.).

##### Etymology.

The name is from the Greek *mystron* (spoon) -*phyllus* (-leaved), in reference to its spoon-shaped leaves.

#### Goodenia panduriformis

Taxon classificationPlantaeAsteralesGoodeniaceae

(A.Cunn. ex Benth.) K.A.Sheph. comb. nov.

EBD560F5-8365-5483-9B06-A9A546284390

urn:lsid:ipni.org:names:77210093-1

≡ Velleia panduriformis A.Cunn. ex Benth., Fl. Austral. 4: 46. 1868 – Lectotype (first-step designated by Carolin, Proc. Linn. Soc. New South Wales 92(1): 36 (1967): “Goodenough Bay and Point Cunningham, N.W. Coast, A. Cunningham (K)”; second-step (designated here): Australia: Western Australia. Point Cunningham & Carlisle Head, the North Point of Goodenough Bay, *s. dat.*, [*A.Cunningham s.n.*] (K 000215368 [image!]; isolectotypes: BM 00104382 [image!], K 000215367 [image!], MEL 9640 [image!])).

##### Note.

Carolin (1967c) designated a collection by Alan Cunningham from “Goodenough Bay and Point Cunningham” at K as the first-step lectotype of *Velleia panduriformis* A.Cunn. ex Benth. The collection designated as lectotype by Carolin comprises two fertile gatherings mounted on one sheet, which have subsequently been treated as two separate accessions with different barcode identifiers. The smaller of these two gatherings (K 000215368), which bears a label in Cunningham’s hand, is here chosen as the second-step lectotype.

#### Goodenia paradoxa

Taxon classificationPlantaeAsteralesGoodeniaceae

(R.Br.) K.A.Sheph. comb. nov.

AAD41B43-25C8-5AE8-AF2A-9DAF8BD3253D

urn:lsid:ipni.org:names:77210094-1

≡ Velleia paradoxa R.Br., Prodr. 580. 1810 – Lectotype (designated by Carolin, Proc. Linn. Soc. New South Wales s92(1): 45 (1967): Australia. New South Wales. Cow pasture plains, Oct. 1803, *R.Brown s.n.* [Bennett no. 2547] (BM 001041380 (two right-hand specimens only) [image!]; isolectotypes: BM 001041381 [image!], CANB 279053!, K 000215386 [image!],; probable isolectotypes: BM 001041379 (two left-hand specimens only) [image!], BRI AQ225859) [image!], MEL 9866 (left-hand specimens marked ‘A’ on sheet) [image!], NSW 78419 [image!], P 00698803 [image!])).

#### Goodenia parvisepta

Taxon classificationPlantaeAsteralesGoodeniaceae

(Carolin) K.A.Sheph. comb. nov.

FD4F489B-25D1-5DEB-9F8A-272BA92CD789

urn:lsid:ipni.org:names:77210095-1

≡ Velleia parvisepta Carolin, Proc. Linn. Soc. New South Wales 92: 49. 1967 – Holotype: AUSTRALIA. New South Wales. Dubbo, 8 Nov 1960, *J.Peacock s.n.* (NSW 100660 [image!]).

#### Goodenia perfoliata

Taxon classificationPlantaeAsteralesGoodeniaceae

(R.Br.) K.A.Sheph. comb. nov.

46A3F7CD-B944-5AA6-AD0D-F8D69A4FB22C

urn:lsid:ipni.org:names:77210096-1

≡ Velleia perfoliata R.Br., Prodr. 581. 1810 – Holotype: Australia. New South Wales. Blue Mountains, 1803, *A.Gordon s.n.* (BM 001041391 [image!]).

#### Goodenia radicans

Taxon classificationPlantaeAsteralesGoodeniaceae

(Cav.) Pers., Syn. pl. 1: 195. 1805

63EAF4F0-1C25-5E70-ABB3-0942813B0B85

≡ Selliera radicans Cav., Anales Hist. Nat. 1(1): 41, t. 5, fig. 2. 1799 – Type citation: “Crece con abundancia en los húmedos inmediatos al mar de S. Cárlos de Chilow, ... y tambien en el valle distante apenas un a legua de Coquimbo, ... . El Sr. Née cogió allí esta planta, que he visto en su herbario, como también el dibuxo que mandó sacar.” **Lectotype (designated here)**: Chile. Carlos y Coquimbo [as ‘Arica y Coquimbo’ on additional, typewritten label (translation of original handwritten label)], *s. dat., L.Née 715* (MA 476260 [image!]. Probable isolectotype: CHILE. In portu Coquimbo … [as ‘San Carlos y Coquimbo (Chile)’ on additional, typewritten label (translation of original handwritten labels)], *s. dat.*, *L. Née s.n.* (MA 476261 [image!]).
= Goodenia repens Labill., Nov. Holl. Pl. 1(5–7): 53, t. 76. 1805 – Type citation: “HABITAT in capite Van-Diemen.” **Lectotype (designated here)**: … Terra Diemen, *s. dat., J.J.H. Labillardière s.n.* (FI 006937 (ex Herb. Webb) [image!]; probable isolectotypes: FI 006938 [image!]; G-DC 00322613 [image!]; P 00698714 [image!]).
= Selliera herpystica Schltdl., Linnaea 20: 598. 1847 – Holotype (*fide* Heuchert et al., Schlentendalia 31: 116. 2017): Australia. South Australia. Südaustralien, auf torfigem im Winter überschwemmten Boden an dem Gawlerriver bei Benthanien, Feb 1845, *H.H.Behr s.n.* (HAL 0098334 [image!]).
= Selliera microphylla Colenso, Trans. & Proc. New Zealand Inst. 22: 473. 1889 [1890]. Probable syntypes: New Zealand. Without precise locality, May 1890, *W. Colenso s.n.* (K 000741872 [image!]; New Zealand. Without precise locality, *s. dat.* (WELT 52409 [image!]); New Zealand. Tongariro … 1889, *H.Hill s.n.* (WELT 59062 [image!], WELT 59063 [image!], WELT 59064 [image!]).

##### Note.

*Nee 715* (MA 476260) is here selected as the lectotype of *Selliera radicans* Cav., as it is the most complete of the available syntypes at MA, bearing both flowers and fruit. *Labillardière s.n.* (FI 006937) is here selected as the lectotype of *Goodenia repens* Labill. The specimen is extensively annotated by Labillardiere, and was formerly part of the Philip Webb herbarium (Webb acquired Labillardiere’s herbarium in 1834; see Stafleu & Cowan 1979).

#### Goodenia reinwardtii

Taxon classificationPlantaeAsteralesGoodeniaceae

(de Vriese) K.A.Sheph. comb. nov.

A1DFB0BF-B063-5AD8-A5D7-509DB3450376

urn:lsid:ipni.org:names:77210097-1

≡ Scaevola reinwardtii de Vriese in J.G.C.Lehmann, Pl. Preiss. 1(3): 409. 1845 ≡ Verreauxia reinwardtii (de Vriese) Benth., Fl. Austral. 4: 105. 1868 – Lectotype (designated by Carolin in A.S.George (ed.), Fl. Austral. 35: 334. 1992): Australia. Western Australia. In planitis arenosa “Quangen” (Victoria), 20 Mar 1840, *L.Preiss* [Plantae Preissianae 1454] (LD 1821186 [image!]; isolectotypes: MEL 42187 [image!], MEL 2192672 [image!]); probable isolectotypes: P 00698676 [image!]), S S08-4783 [image!]).

#### Goodenia rosea

Taxon classificationPlantaeAsteralesGoodeniaceae

(S.Moore) K.A.Sheph. comb. nov.

4A1D05C9-978A-5867-80A0-971D7FB1DB2D

urn:lsid:ipni.org:names:77210098-1

≡ Velleia rosea S.Moore, J. Linn. Soc. Bot. 34: 202. 1898 – Holotype: Australia. Western Australia. Creek between Wilson’s Pool and Lake Darlot, Apr. 1895, *S. Moore s.n.* (BM 001041378 [image!]).

#### Goodenia subsolana

Taxon classificationPlantaeAsteralesGoodeniaceae

K.A.Sheph. nom. nov.

B32F3E40-530B-5D79-919E-DD3ED9BCF18C

urn:lsid:ipni.org:names:77210099-1

≡ Velleia pubescens R.Br., Prodr. 581. 1810 – Syntypes: Australia. Queensland. Shoalwater Bay and Broad Sound, *s. dat.*, [*R.Brown s.n.*] (BM 001041383 [image!], BM 001041384 [image!], CANB 279054!, K 000215429 [image!], (K 000215430 [image!]), MEL 9796) [image!], P 00698800 [image!].

##### Note.

The epithet ‘pubescens’ is unavailable in *Goodenia* as it is preoccupied by *Goodenia pubescens* Sieber ex Spreng. (= *Scaevola albida* (Sm.) Druce).

Carolin (1992g) lists two syntypes at BM as the ‘lectotype’ for *Velliea pubescens* R.Br. and reports lectotypification as having been effected in his 1967 treatment of the genus *Velleia*, viz: “Shoalwater Bay, and Thirsty Sound, [Qld], *R. Brown 87*; lecto: BM, *fide* R.C.Carolin, *Proc. Linn. Soc. New South Wales* 92: 53 (1963) [*sic*; 1967]; isolecto: MEL, P.” However, Carolin (1967c, 1992g) does not effectively lectotypify *Velleia pubescens* as he does not clearly indicate the type element by direct citation, as required by ICN Art. 7.11, rather citing two syntype specimens at BM. The gatherings referred to by Carolin (1967c, 1992g) comprise three flowering plants, mounted on one sheet (BM 001041383), and, as noted by Carolin (1967c), Brown’s original labels, formerly affixed to the specimens themselves, have been detached and glued to the sheet, thereby making it impossible to determine which specimen relates to which label. A lectotype has not been designated for *Velleia pubescens* R.Br., as this will be effected by D.J.Mabberley in a forthcoming publication on the life and work of Robert Brown (D.J.Mabberley pers. comm. 2020).

##### Etymology.

This species is named for the Latin *subsolanus* (eastern, oriental) as this species is found near coastal habitats of Queensland in eastern Australia.

#### Goodenia trinervis

Taxon classificationPlantaeAsteralesGoodeniaceae

(Labill.) K.A.Sheph. comb. nov.

A004E986-F88B-5CB8-8C9A-737A958CD86B

urn:lsid:ipni.org:names:77210100-1

≡ Velleia trinervis Labill. Nov. Holl. Pl. 1(5–7): 54, t. 77. 1805 – Lectotype (designated by Carolin, Proc. Linn. Soc. New South Wales 92(1): 31. 1967: Australia. Tasmania. Nouvelle Hollande, Côte S. O., *s. dat., J.J.H.Labillardiere s.n.* (P 00698795 (ex Herb. Webb) [image!]; probable isolectotypes: BM 001041376 [image!], B-W 04026 [image!], FI 113248 [image!], G 00355635 [image!], G-DC 00322623 [image!], MEL 9651 [image!], P 00698794 [image!], P 00698796 [image!])).

#### Goodenia verreauxii

Taxon classificationPlantaeAsteralesGoodeniaceae

(de Vriese) K.A.Sheph. comb. nov.

960D6BC3-8092-5EB1-87D6-2E5E6FCD59B7

urn:lsid:ipni.org:names:77210101-1

≡ Dampiera verreauxii de Vriese, Natuurk. Verh. Holl. Maatsch. Wetensch. Haarlem ser. 2, 10: 118, t. 20. 1854 ≡ Verreauxia paniculata Benth., Fl. Austral. 4: 105. 1868, *nom. illeg., nom. superfl.* ≡ Verreauxia verreauxii (de Vriese) Carolin, Telopea 2(1): 75. 1980. Type citation: “Nov. Holl. specimine mihi humanissime oblato cum mutlis aliis plantis Novae Hollandiae, a Celeb. Inventore Verreaux, dum hoc. anno 1850 in nostre urbe degebat. Plurimas etiam alias stirpes ab hoc Naturae Investigore repertas et ad Goodenovieas reletas, vidi in Herb. Musei Horti Parisiensis.”

##### Lectotype (here designated).

“Dampiera verreauxii” in de Vriese, Natuurk. Verh. Holl. Maatsch. Wetensch. Haarlem ser. 2, 10: t. 20. (1854).

##### Note.

No specimens are cited by de Vriese (1854) in the protologue for *Dampiera verreauxii*, although de Vriese indicates that he examined material of this taxon made available by Verreaux at the Muséum National d’Histoire Naturelle in Paris (P) in 1850. Verreaux visited Australia during 1842–1846 but his collecting efforts were confined to Tasmania and the east coast of Australia (George 2009) and so it is unlikely he would have obtained material from Western Australia directly. Carolin (1992i) postulated that “The type was probably collected by J. Drummond (*Drummond* 4: 186 cited by Krause, loc. cit. [= Pflanzenr. 54: 170 (1912)])”. This is possible, as Drummond did collect extensively through south-west Western Australia (including the region where this species occurs) and his specimens were sent to various institutions throughout Australia and Europe. Three Drummond collections of this taxon have been located (MEL 42188 [image!], P 03035588 [image!] and P 04057856 [image!]). However, it is not clear whether these specimens represent original material and neither specimen at P is an exact match for the plant illustrated in the protologue. Accordingly, the illustration included in the protologue is here designated as the lectotype for *Dampiera verreauxii* de Vriese.

#### Scaevola filifolia

Taxon classificationPlantaeAsteralesGoodeniaceae

(R.Br.) K.A.Sheph. comb. nov.

34EC7AE6-6D92-5115-AA5C-1C4D24CB2EBA

urn:lsid:ipni.org:names:77210372-1

≡ Diaspasis filifolia R.Br., Prodr. 587. 1810. *Type citation*: “(M.) v.v.” Syntypes: Australia. Western Australia. King George III’s Sound, 21 Dec 1801, *R. Brown s.n.* [Bennett No. 2659] (BM 001041412 [image!], BM 001041413 [image!]; K 000216450 [image!]; K 000216453 [image!]; P 00698693 [image!])).
= Goodenia glandulifera de Vriese, Natuurk. Verh. Holl. Maatsch. Wetensch. Haarlem ser. 2, 10: 129. 1854 – Lectotype (designated by Carolin, Telopea, 3(4): 566 (1990): Australia. Western Australia. In solo turfoso inter frutices prope urbiculam “Albany” (Plantagenet), 4 Oct. 1840, *L.Preiss s.n.* [Plantae Preissiana 2032] (LD 1677627 [image!]; isolectotype: L 0012072 [image!]).
= Scaevola clandestina F.Muell., Fragm. 1(9): 206. 1859 – Type citation: “In Nova Hollandia austro-occidentali.” Type: *n.v*.

##### Note.

A lectotype has not been designated for *Diaspasis filifolia* R.Br., as this will be effected by D.J.Mabberley in a forthcoming publication on the life and work of Robert Brown (D.J.Mabberley pers. comm. 2020).

## Supplementary Material

XML Treatment for Scaevola

XML Treatment for Goodenia

XML Treatment for Goodenia Sm. subg. Goodenia

XML Treatment for Goodenia Sm. subg. Goodenia sect. Goodenia

XML Treatment for Goodenia subg. Goodenia sect. Rosulatae

XML Treatment for Goodenia subg. Porphyranthus

XML Treatment for Goodenia subg. Porphyranthus sect. Porphyranthus

XML Treatment for Goodenia subg. Porphyranthus sect. Ebracteolatae

XML Treatment for Goodenia subg. Monochila

XML Treatment for Goodenia subg. Monochila sect. Monochila

XML Treatment for Goodenia subg. Monochila

XML Treatment for Goodenia subg. Monochila sect. Monochila subsect. Infracta

XML Treatment for Goodenia subg. Monochila sect. Scaevolina

XML Treatment for Goodenia subg. Monochila sect. Coeruleae

XML Treatment for Goodenia subg. Monochila sect. Tetrathylax

XML Treatment for Goodenia subg. Monochila sect. Verreauxia

XML Treatment for Goodenia subg. Monochila sect. Velleia

XML Treatment for Goodenia arguta

XML Treatment for Goodenia brendannarum

XML Treatment for Goodenia capillosa

XML Treatment for Goodenia careyi

XML Treatment for Goodenia caroliniana

XML Treatment for Goodenia collaris

XML Treatment for Goodenia connata

XML Treatment for Goodenia cycnopotamica

XML Treatment for Goodenia daviesii

XML Treatment for Goodenia discophora

XML Treatment for Goodenia etheira

XML Treatment for Goodenia exigua

XML Treatment for Goodenia glabrata

XML Treatment for Goodenia heenanii

XML Treatment for Goodenia macrocalyx

XML Treatment for Goodenia macrophylla

XML Treatment for Goodenia montana

XML Treatment for Goodenia mystrophylla

XML Treatment for Goodenia panduriformis

XML Treatment for Goodenia paradoxa

XML Treatment for Goodenia parvisepta

XML Treatment for Goodenia perfoliata

XML Treatment for Goodenia radicans

XML Treatment for Goodenia reinwardtii

XML Treatment for Goodenia rosea

XML Treatment for Goodenia subsolana

XML Treatment for Goodenia trinervis

XML Treatment for Goodenia verreauxii

XML Treatment for Scaevola filifolia

## Supplementary materials

Supplementary material 1 Summary of GenBank accession numbers, Taxon names, Project numbers, Herbarium Accession numbers, voucher collectors and collection numbers, phylogenetic position and taxonomy and classification according to Shepherd et al. Data type species data https://binary.pensoft.net/file/429363This dataset is made available under the Open Database License (http://opendatacommons.org/licenses/odbl/1.0/). The Open Database License (ODbL) is a license agreement intended to allow users to freely share, modify, and use this Dataset while maintaining this same freedom for others, provided that the original source and author(s) are credited.Kelly A. Shepherd, Brendan J. Lepschi, Eden A. Johnson, Andrew G. Gardner, Emily B. Sessa and Rachel S. Jabaily

Supplementary material 2 Goodenia Clade A cpDNA (trnL-F, matK) phylogeny Data type PDF File Explanation note Phylogeny of concatenated chloroplast loci trnL-F and matK for Goodenia Clade A https://binary.pensoft.net/file/429364This dataset is made available under the Open Database License (http://opendatacommons.org/licenses/odbl/1.0/). The Open Database License (ODbL) is a license agreement intended to allow users to freely share, modify, and use this Dataset while maintaining this same freedom for others, provided that the original source and author(s) are credited.Kelly A. Shepherd, Brendan J. Lepschi, Eden A. Johnson, Andrew G. Gardner, Emily B. Sessa and Rachel S. Jabaily

Supplementary material 3 Goodenia Clade A nrITS phylogeny Data type PDF File https://binary.pensoft.net/file/429365This dataset is made available under the Open Database License (http://opendatacommons.org/licenses/odbl/1.0/). The Open Database License (ODbL) is a license agreement intended to allow users to freely share, modify, and use this Dataset while maintaining this same freedom for others, provided that the original source and author(s) are credited.Kelly A. Shepherd, Brendan J. Lepschi, Eden A. Johnson, Andrew G. Gardner, Emily B. Sessa and Rachel S. Jabaily

Supplementary material 4 Goodenia Clade B cpDNA (trnL-F, matK) phylogeny Data type PDF File Explanation note Phylogeny of concatenated chloroplast loci trnL-F and matK for Goodenia Clade B https://binary.pensoft.net/file/429366This dataset is made available under the Open Database License (http://opendatacommons.org/licenses/odbl/1.0/). The Open Database License (ODbL) is a license agreement intended to allow users to freely share, modify, and use this Dataset while maintaining this same freedom for others, provided that the original source and author(s) are credited.Kelly A. Shepherd, Brendan J. Lepschi, Eden A. Johnson, Andrew G. Gardner, Emily B. Sessa and Rachel S. Jabaily

Supplementary material 5 Goodenia Clade B nrITS phylogeny Data type PDF File https://binary.pensoft.net/file/429367This dataset is made available under the Open Database License (http://opendatacommons.org/licenses/odbl/1.0/). The Open Database License (ODbL) is a license agreement intended to allow users to freely share, modify, and use this Dataset while maintaining this same freedom for others, provided that the original source and author(s) are credited.Kelly A. Shepherd, Brendan J. Lepschi, Eden A. Johnson, Andrew G. Gardner, Emily B. Sessa and Rachel S. Jabaily

Supplementary material 6 Goodenia Clade C cpDNA (trnL-F, matK) phylogeny Data type PDF File Explanation note Phylogeny of concatenated chloroplast loci trnL-F and matK for Goodenia Clade C https://binary.pensoft.net/file/429368This dataset is made available under the Open Database License (http://opendatacommons.org/licenses/odbl/1.0/). The Open Database License (ODbL) is a license agreement intended to allow users to freely share, modify, and use this Dataset while maintaining this same freedom for others, provided that the original source and author(s) are credited.Kelly A. Shepherd, Brendan J. Lepschi, Eden A. Johnson, Andrew G. Gardner, Emily B. Sessa and Rachel S. Jabaily

Supplementary material 7 Goodenia Clade C nrITS phylogeny Data type PDF File Explanation note Phylogeny of nrITS for Goodenia Clade C https://binary.pensoft.net/file/429369This dataset is made available under the Open Database License (http://opendatacommons.org/licenses/odbl/1.0/). The Open Database License (ODbL) is a license agreement intended to allow users to freely share, modify, and use this Dataset while maintaining this same freedom for others, provided that the original source and author(s) are credited.Kelly A. Shepherd, Brendan J. Lepschi, Eden A. Johnson, Andrew G. Gardner, Emily B. Sessa and Rachel S. Jabaily

Supplementary material 8 Goodenia Clade A cpDNA (trnL-F, matK) alignment Data type Nexus formatted alignment Explanation note Alignment of concatenated chloroplast loci trnL-F and matK for Goodenia Clade A https://binary.pensoft.net/file/429370This dataset is made available under the Open Database License (http://opendatacommons.org/licenses/odbl/1.0/). The Open Database License (ODbL) is a license agreement intended to allow users to freely share, modify, and use this Dataset while maintaining this same freedom for others, provided that the original source and author(s) are credited.Kelly A. Shepherd, Brendan J. Lepschi, Eden A. Johnson, Andrew G. Gardner, Emily B. Sessa and Rachel S. Jabaily

Supplementary material 9 Goodenia Clade A nrITS alignment Data type Nexus formatted alignment https://binary.pensoft.net/file/429371This dataset is made available under the Open Database License (http://opendatacommons.org/licenses/odbl/1.0/). The Open Database License (ODbL) is a license agreement intended to allow users to freely share, modify, and use this Dataset while maintaining this same freedom for others, provided that the original source and author(s) are credited.Kelly A. Shepherd, Brendan J. Lepschi, Eden A. Johnson, Andrew G. Gardner, Emily B. Sessa and Rachel S. Jabaily

Supplementary material 10 Goodenia Clade B cpDNA (trnL-F, matK) alignment Data type Nexus formatted alignment Explanation note Alignment of concatenated chloroplast loci trnL-F and matK for Goodenia Clade B https://binary.pensoft.net/file/429372This dataset is made available under the Open Database License (http://opendatacommons.org/licenses/odbl/1.0/). The Open Database License (ODbL) is a license agreement intended to allow users to freely share, modify, and use this Dataset while maintaining this same freedom for others, provided that the original source and author(s) are credited.Kelly A. Shepherd, Brendan J. Lepschi, Eden A. Johnson, Andrew G. Gardner, Emily B. Sessa and Rachel S. Jabaily

Supplementary material 11 Goodenia Clade B nrITS alignment Data type Nexus formatted alignment https://binary.pensoft.net/file/429373This dataset is made available under the Open Database License (http://opendatacommons.org/licenses/odbl/1.0/). The Open Database License (ODbL) is a license agreement intended to allow users to freely share, modify, and use this Dataset while maintaining this same freedom for others, provided that the original source and author(s) are credited.Kelly A. Shepherd, Brendan J. Lepschi, Eden A. Johnson, Andrew G. Gardner, Emily B. Sessa and Rachel S. Jabaily

Supplementary material 12 Goodenia Clade C cpDNA (trnL-F, matK) alignment Data type Nexus formatted alignment Explanation note Alignment of concatenated chloroplast loci trnL-F and matK for Goodenia Clade C https://binary.pensoft.net/file/429374This dataset is made available under the Open Database License (http://opendatacommons.org/licenses/odbl/1.0/). The Open Database License (ODbL) is a license agreement intended to allow users to freely share, modify, and use this Dataset while maintaining this same freedom for others, provided that the original source and author(s) are credited.Kelly A. Shepherd, Brendan J. Lepschi, Eden A. Johnson, Andrew G. Gardner, Emily B. Sessa and Rachel S. Jabaily
