# Supplementary figures and images for: The concluding chapter: recircumscription of Goodenia (Goodeniaceae) to include four allied genera with an updated infrageneric classification
Source: PhytoKeys. 2020 Jul 7;152:27–104. doi: 10.3897/phytokeys.152.49604 (PMC7360637; doi:10.3897/phytokeys.152.49604)

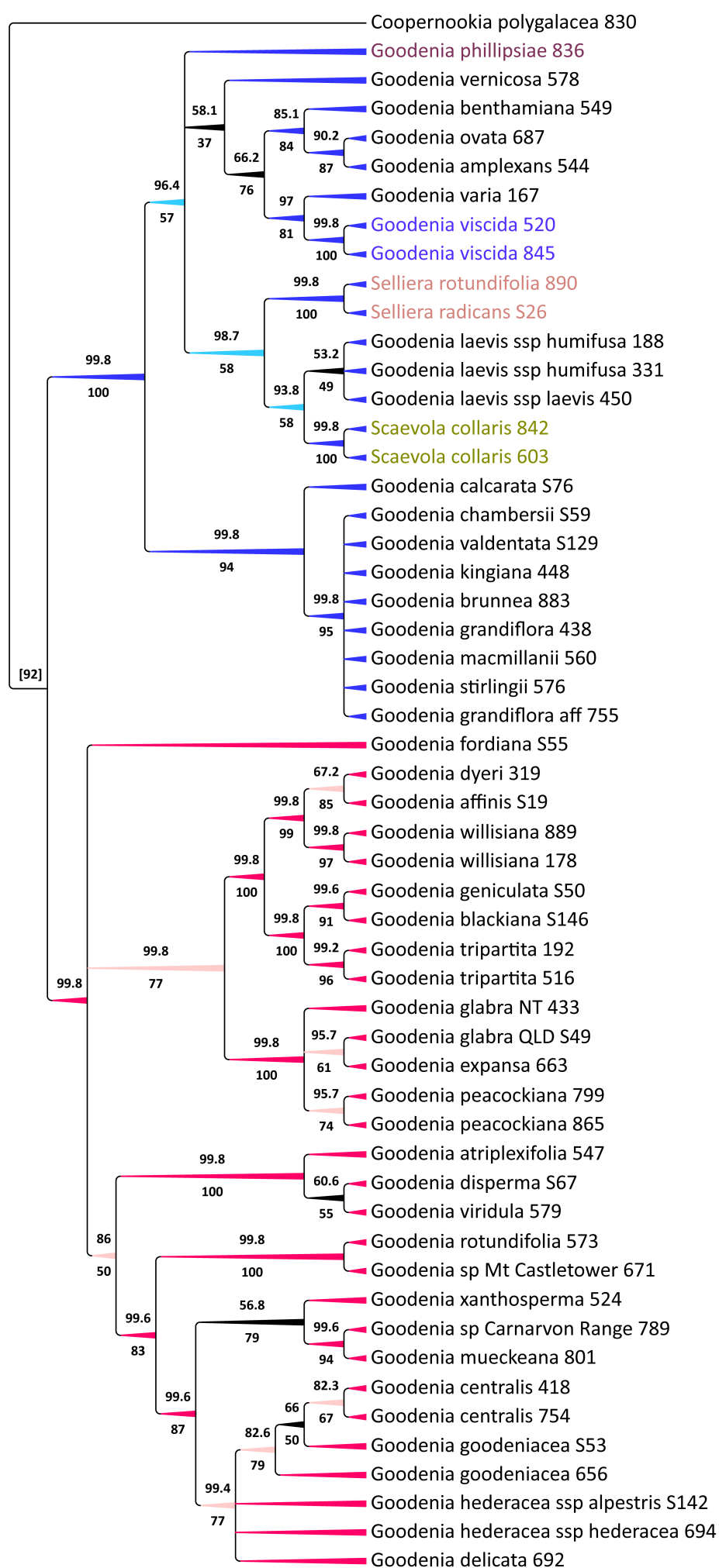

Goodenia I

Goodenia II

subsect. *Ebracteolatae*  
subgen. *Monochila*  
*Selliera*  
*Scaevola*

0.0

Supplement: Supplementary material 1 — Summary of GenBank accession numbers, Taxon names, Project numbers, Herbarium Accession numbers, voucher collectors and collection numbers, phylogenetic position and taxonomy and classification according to Shepherd et al. [file phytokeys-152-027-s001.pdf]

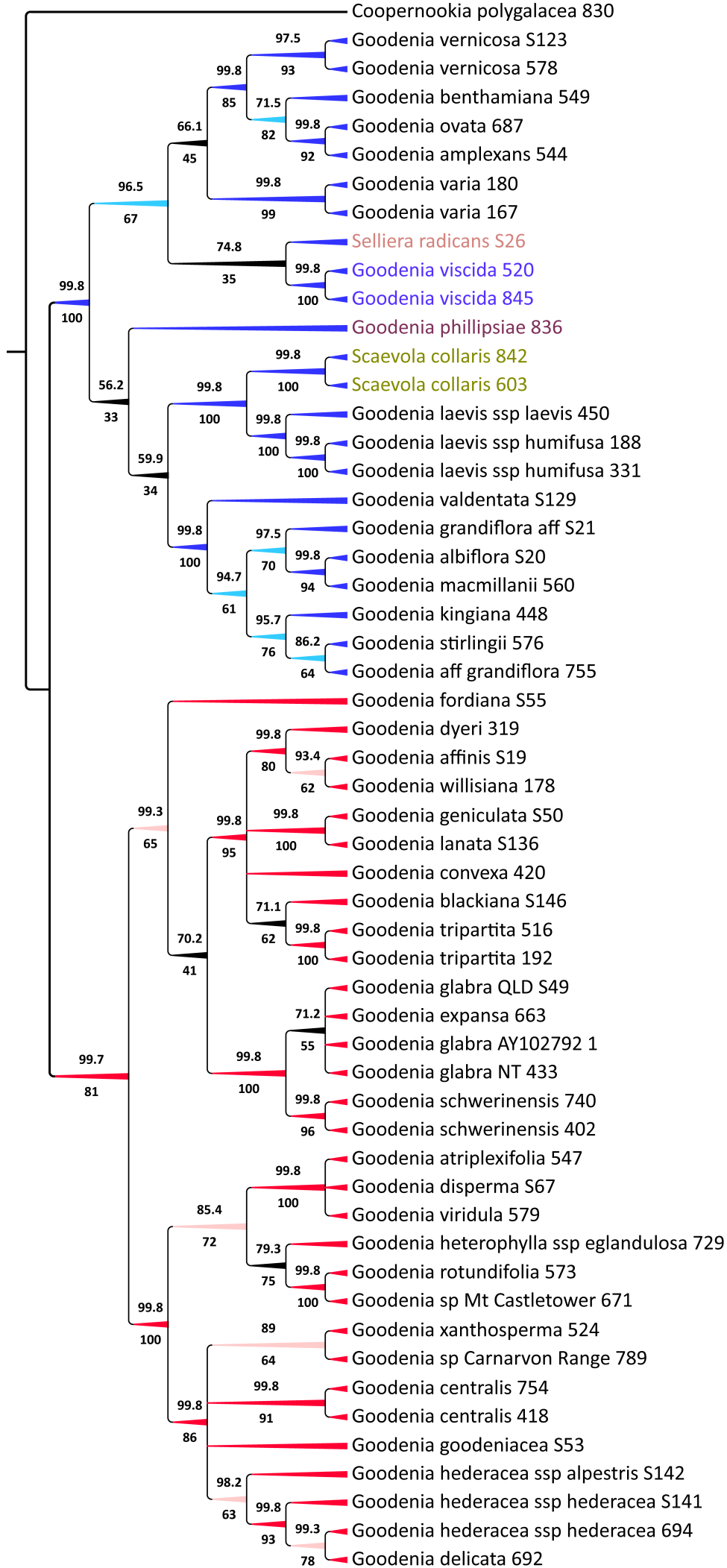

Goodenia I

Goodenia II

subject. Ebracteolatae  
subgen. Monochila  
Selliera  
Scaevola

Supplement: Supplementary material 2 — Goodenia Clade A cpDNA (trnL-F, matK) phylogeny [file phytokeys-152-027-s002.pdf]

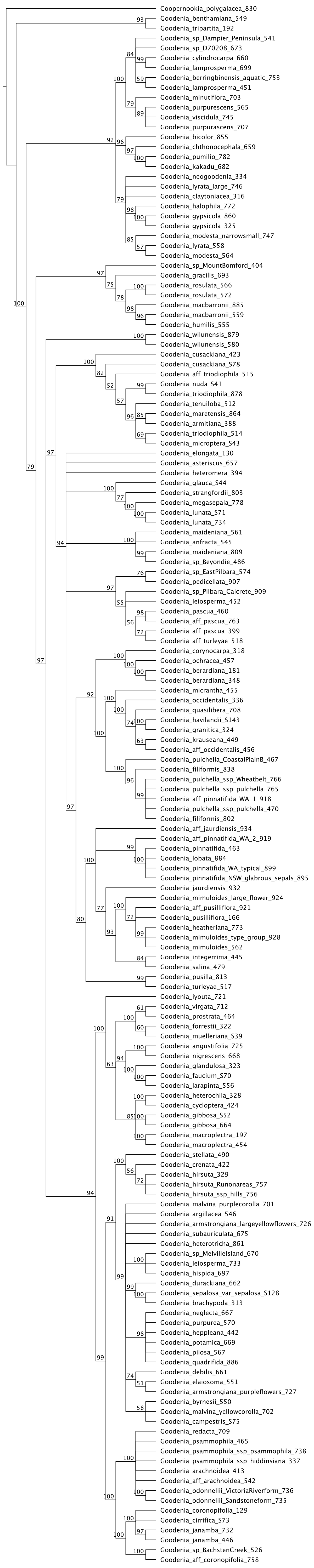

Supplement: Supplementary material 3 — Goodenia Clade A nrITS phylogeny [file phytokeys-152-027-s003.pdf]

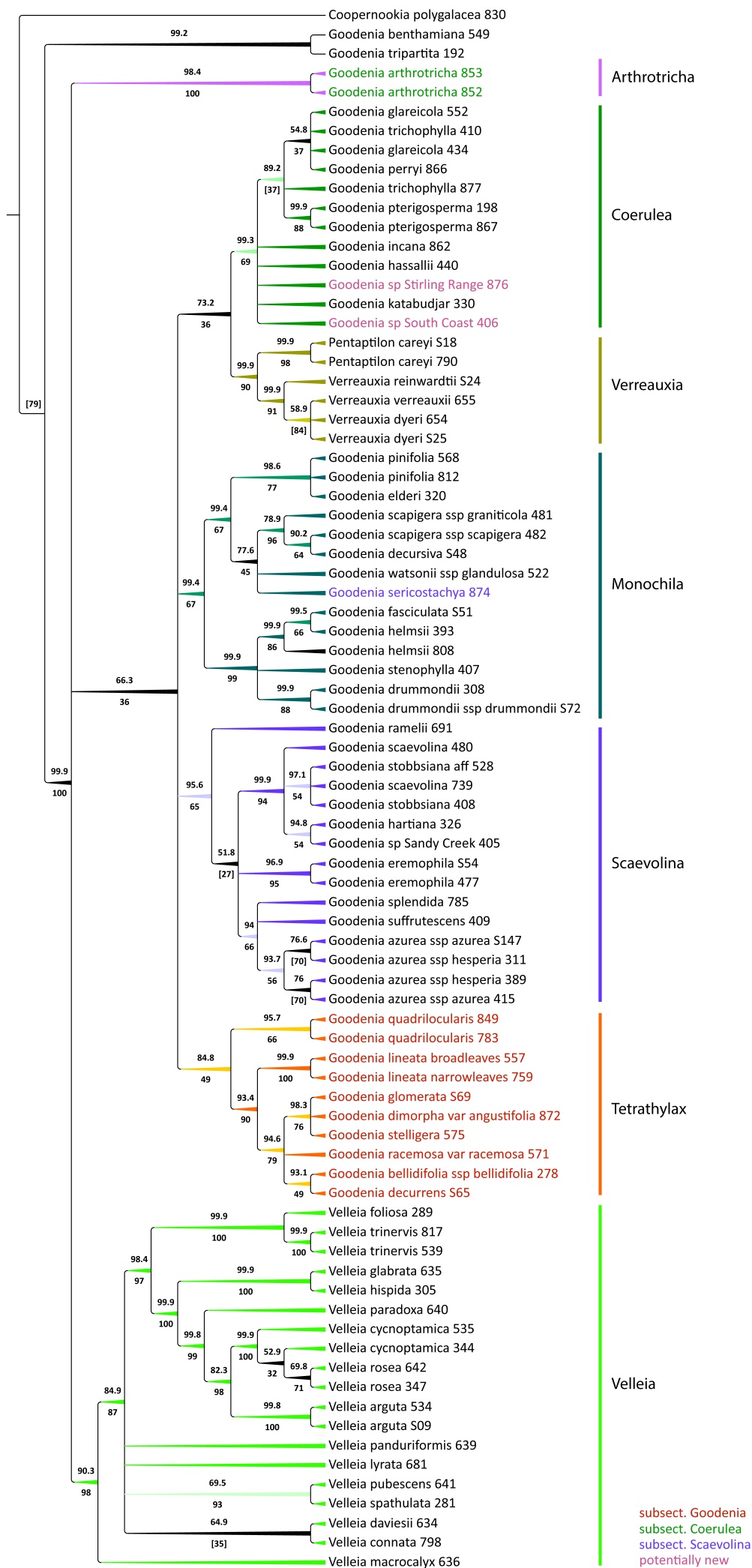

Supplement: Supplementary material 5 — Goodenia Clade B nrITS phylogeny [file phytokeys-152-027-s005.pdf]

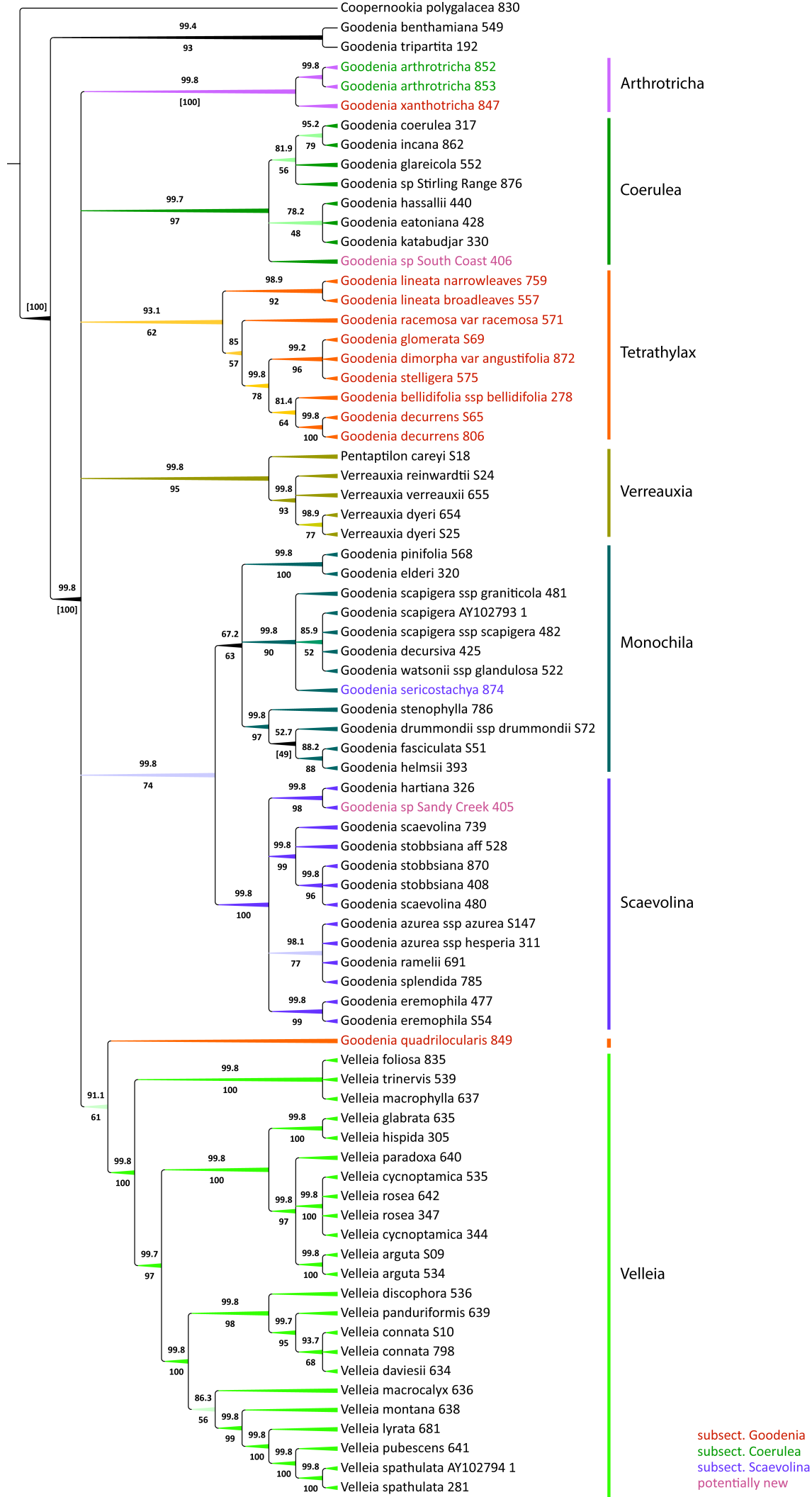

Supplement: Supplementary material 6 — Goodenia Clade C cpDNA (trnL-F, matK) phylogeny [file phytokeys-152-027-s006.pdf]
