# Supplementary material for: The concluding chapter: recircumscription of Goodenia (Goodeniaceae) to include four allied genera with an updated infrageneric classification
Source: PhytoKeys. 2020 Jul 7;152:27–104. doi: 10.3897/phytokeys.152.49604 (PMC7360637; doi:10.3897/phytokeys.152.49604)
Supplement: Supplementary material 4 — Goodenia Clade B cpDNA (trnL-F, matK) phylogeny [file phytokeys-152-027-s004.pdf]

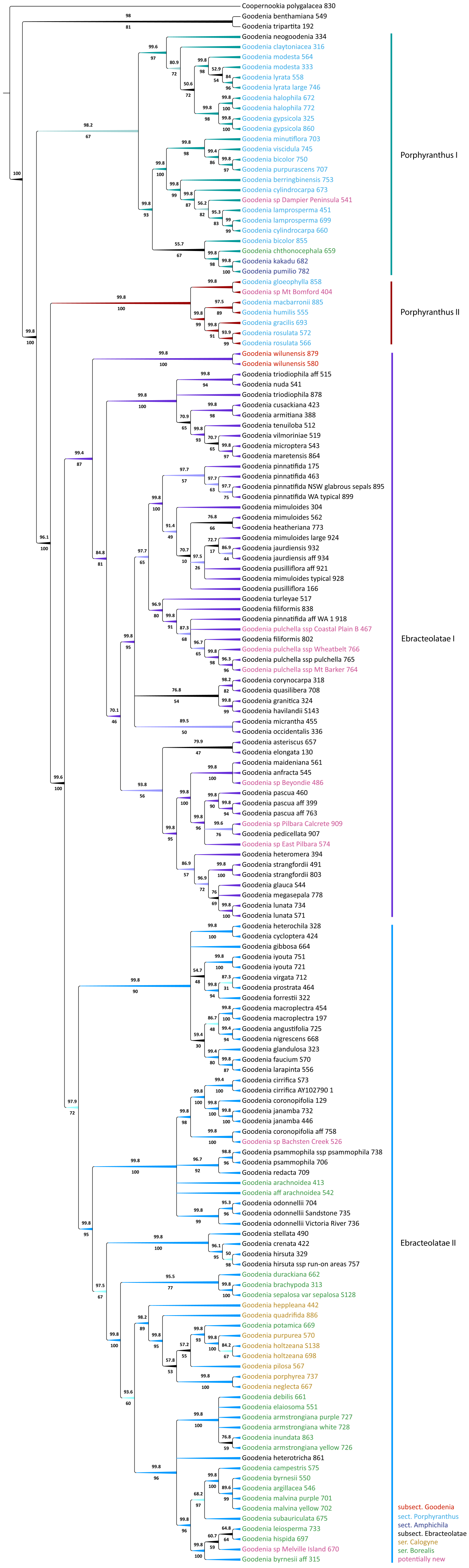

Coopernookia polygalacea 830
Goodenia benthamiana 549
Goodenia tripartita 192
Goodenia neogoodenia 334
Goodenia claytoniacea 316
Goodenia modesta 564
Goodenia modesta 333
Goodenia lyrata 558
Goodenia lyrata large 746
Goodenia halophila 672
Goodenia halophila 772
Goodenia gypsicola 325
Goodenia gypsicola 860
Goodenia minutiflora 703
Goodenia viscidula 745
Goodenia bicolor 750
Goodenia purpurascens 707
Goodenia berringbinensis 753
Goodenia cylindrocarpa 673
Goodenia sp Dampier Peninsula 541
Goodenia lamprosperma 451
Goodenia lamprosperma 699
Goodenia cylindrocarpa 660
Goodenia bicolor 855
Goodenia chthonocephala 659
Goodenia kakadu 682
Goodenia pumilio 782
Porphyranthus I
Goodenia gloeophylla 858
Goodenia sp Mt Bomford 404
Goodenia macbarronii 885
Goodenia humilis 555
Goodenia gracilis 693
Goodenia rosulata 572
Goodenia rosulata 566
Porphyranthus II
Goodenia wilunensis 879
Goodenia wilunensis 580
Goodenia triodiophila aff 515
Goodenia nuda S41
Goodenia triodiophila 878
Goodenia cusackiana 423
Goodenia armitiana 388
Goodenia tenuiloba 512
Goodenia vilmoriniae 519
Goodenia microptera S43
Goodenia maretensis 864
Goodenia pinnatifida 175
Goodenia pinnatifida 463
Goodenia pinnatifida NSW glabrous sepals 895
Goodenia pinnatifida WA typical 899
Goodenia mimuloides 304
Goodenia mimuloides 562
Goodenia heatheriana 773
Goodenia mimuloides large 924
Goodenia jaurdiensis 932
Goodenia jaurdiensis aff 934
Goodenia pusilliflora aff 921
Goodenia mimuloides typical 928
Goodenia pusilliflora 166
Goodenia turleyae 517
Goodenia filiformis 838
Goodenia pinnatifida aff WA 1 918
Goodenia pulchella ssp Coastal Plain B 467
Goodenia filiformis 802
Goodenia pulchella ssp Wheatbelt 766
Goodenia pulchella ssp pulchella 765
Goodenia pulchella ssp Mt Barker 764
Goodenia corynocarpa 318
Goodenia quasilibera 708
Goodenia granitica 324
Goodenia havilandii S143
Goodenia micrantha 455
Goodenia occidentalis 336
Goodenia asteriscus 657
Goodenia elongata 130
Goodenia maideniana 561
Goodenia anfracta 545
Goodenia sp Beyondie 486
Goodenia pascua 460
Goodenia pascua aff 399
Goodenia pascua aff 763
Goodenia sp Pilbara Calcrete 909
Goodenia pedicellata 907
Goodenia sp East Pilbara 574
Goodenia heteromera 394
Goodenia strangfordii 491
Goodenia strangfordii 803
Goodenia glauca S44
Goodenia megasepala 778
Goodenia lunata 734
Goodenia lunata S71
Ebracteolatae I
Goodenia heterochila 328
Goodenia cycloptera 424
Goodenia gibbosa 664
Goodenia iyouta 751
Goodenia iyouta 721
Goodenia virgata 712
Goodenia prostrata 464
Goodenia forrestii 322
Goodenia macroplectra 454
Goodenia macroplectra 197
Goodenia angustifolia 725
Goodenia nigrescens 668
Goodenia glandulosa 323
Goodenia faucium S70
Goodenia larapinta 556
Goodenia cirrifica S73
Goodenia cirrifica AY102790 1
Goodenia coronopifolia 129
Goodenia janamba 732
Goodenia janamba 446
Goodenia coronopifolia aff 758
Goodenia sp Bachsten Creek 526
Goodenia psammophila ssp psammophila 738
Goodenia psammophila 706
Goodenia redacta 709
Goodenia arachnoidea 413
Goodenia aff arachnoidea 542
Goodenia odonnellii 704
Goodenia odonnellii Sandstone 735
Goodenia odonnellii Victoria River 736
Goodenia stellata 490
Goodenia crenata 422
Goodenia hirsuta 329
Goodenia hirsuta ssp run-on areas 757
Goodenia durackiana 662
Goodenia brachypoda 313
Goodenia sepalosa var sepalosa S128
Goodenia heppleana 442
Goodenia quadrifida 886
Goodenia potamica 669
Goodenia purpurea 570
Goodenia holtzeana S138
Goodenia holtzeana 698
Goodenia pilosa 567
Goodenia porphyrea 737
Goodenia neglecta 667
Goodenia debilis 661
Goodenia elaiosoma 551
Goodenia armstrongiana purple 727
Goodenia armstrongiana white 728
Goodenia inundata 863
Goodenia armstrongiana yellow 726
Goodenia heterotricha 861
Goodenia campestris 575
Goodenia byrnesii 550
Goodenia argillacea 546
Goodenia malvina purple 701
Goodenia malvina yellow 702
Goodenia subauriculata 675
Goodenia leiosperma 733
Goodenia hispida 697
Goodenia sp Melville Island 670
Goodenia byrnesii aff 315
Ebracteolatae II
subsect. Goodenia
sect. Porphyranthus
sect. Amphichila
subsect. Ebracteolatae
ser. Calogyne
ser. Borealis
potentially new
